# Maintenance of haematopoietic stem cells by tyrosine-unphosphorylated STAT5 and JAK inhibition

**DOI:** 10.1182/bloodadvances.2024014046

**Published:** 2025-01-28

**Authors:** Matthew J Williams, Xiaonan Wang, Hugo P Bastos, Gabriela Grondys-Kotarba, Qin Wu, Shucheng Jin, Carys Johnson, Nicole Mende, Emily Calderbank, Michelle Wantoch, Hyun Jung Park, Giovanna Mantica, Rebecca Hannah, Nicola K Wilson, Dean C Pask, Tina L Hamilton, Sarah J Kinston, Ryan Asby, Rachel Sneade, Joanna Baxter, Peter Campbell, George S Vassiliou, Elisa Laurenti, Juan Li, Berthold Göttgens, Anthony R Green

**Affiliations:** 1 Wellcome–MRC Cambridge Stem Cell Institute, Jeffrey Cheah Biomedical Centre, University of Cambridge, Cambridge, United Kingdom; 2 Department of Haematology, University of Cambridge, Cambridge, United Kingdom; 3 School of Public Health, Shanghai Jiaotong University School of Medicine, China; 4 Cambridge Institute for Medical Research, Keith Peters Building, Cambridge, United Kingdom; 5 Wellcome Sanger Institute, Wellcome Trust Genome Campus, Hinxton, United Kingdom

## Abstract

Adult haematopoietic stem cells (HSCs) are responsible for the lifelong production of blood and immune cells, a process regulated by extracellular cues including cytokines. Many cytokines signal through the conserved JAK/STAT pathway, in which tyrosine-phosphorylated STATs (pSTATs) function as transcription factors. STAT5 is a pivotal downstream mediator of several cytokines known to regulate haematopoiesis but its function in the HSC compartment remains poorly understood. Here, we show that STAT5-deficient HSCs exhibit an unusual phenotype: reduced multi-lineage repopulation and self-renewal, combined with reduced exit from quiescence and increased differentiation. This was driven not only by loss of canonical pSTAT5 signalling, but also by loss of distinct transcriptional functions mediated by STAT5 lacking canonical tyrosine phosphorylation (uSTAT5). Consistent with this concept, expression of an unphosphorylatable STAT5 mutant constrained wild-type HSC differentiation, promoted their maintenance and upregulated transcriptional programs associated with quiescence and stemness. The JAK1/2 inhibitor, ruxolitinib, which increased the uSTAT5:pSTAT5 ratio, had similar effects on murine HSC function: it constrained HSC differentiation and proliferation, promoted HSC maintenance and upregulated transcriptional programs associated with stemness. Ruxolitinib also enhanced serial replating of normal human HSPCs, CALR-mutant murine HSCs and HSPCs obtained from patients with myelofibrosis. Our results therefore reveal a previously unrecognized interplay between pSTAT5 and uSTAT5 in the control of HSC function and highlight JAK inhibition as a potential strategy for enhancing HSC function during *ex vivo* culture. Increased levels of uSTAT5 may also contribute to the failure of JAK inhibitors to eradicate myeloproliferative neoplasms.

## Introduction

Hematopoietic stem cells (HSCs) are a highly quiescent population of cells responsible for continued production of mature blood cells throughout life.^1,2^ Their ability to respond to environmental signals is important for maintenance of homeostasis and for HSCs to respond to a variety of stresses.^3–6^

The JAK-STAT pathway regulates multiple developmental and adult stem cell populations^7–9^ and is dysregulated in a variety of haematological malignancies and other cancers.^10,11^ The signal transducer and activator of transcription 5 (STAT5) is an essential downstream mediator of cytokine signalling at multiple stages of haematopoiesis.^12–16^ In eutherian mammals, two closely related STAT5 isoforms,^17^ STAT5A and STAT5B, display distinct and redundant functions in different cell types.^18–21^ Mice lacking both genes, or the N-terminal domains of both genes, develop severe anaemia and leukopenia,^22–26^ associated with reduced survival and proliferation of erythroblasts.^15,16^ Conversely, high levels of STAT5 activity in haematopoietic stem and progenitor cells (HSPCs) drives erythroid differentiation.^27,28^

STAT5A and STAT5B contain critical regulatory tyrosine residues (Y694 and Y699 respectively) that are essential for activation of canonical pSTAT5 target genes.^29,30^ These residues are phosphorylated by Janus-kinases (JAKs)^31^ activated in response to multiple cytokines^3–6^ including IL-3^32^ and thrombopoietin (THPO).^33^ Tyrosine-phosphorylated STAT5 (pSTAT5) accumulates in the nucleus, binds to DNA and regulates transcription of target genes.^34^ STAT5 phosphorylation is transient as pSTAT5 rapidly promotes the expression of negative regulators of JAK-STAT signalling, including suppressors of cytokine signalling (SOCS), tyrosine phosphatases, and protein inhibitors of STATs.^35,36^

Elevated STAT5 phosphorylation is observed in many haematological malignancies^37,38^ and solid tumours.^39,40^ Activation of the JAK-STAT pathway is especially common in the myeloproliferative neoplasms (MPNs), >90% of which contain driver mutations which activate JAK-STAT signalling.^41–46^ JAK inhibitors are used to treat MPN patients with advanced disease^47^ but, although they can result in symptomatic improvement, they rarely reduce allele burden,^48–50^ suggesting that they fail to eradicate malignant HSCs.

Loss of both STAT5 genes results in reduced numbers of immunophenotypically-defined HSCs,^26,51,52^ as well as defective repopulation by foetal liver and adult bone marrow (BM).^26,53,54^ STAT5B is dominant in multipotent HPC7 cells^55^ and STAT5B-deficient, but not STAT5A-deficient, BM showed functional defects in serial transplants.^52^ However several aspects of STAT5 function in HSPCs remain unclear or have been the subject of conflicting reports: both increased^26,51,52^ and reduced cycling^56^ have been observed in HSPCs after STAT5 loss, while STAT5 phosphorylation is associated with increased proliferation.^57^ Moreover both STAT5 knockdown^55^ and constitutively active STAT5A overexpression^27,28^ have been reported to increase HSPC differentiation. Insight into at least some of these apparent paradoxes came from the demonstration that STAT5 lacking phosphorylation of its critical tyrosine (uSTAT5) is present in the nucleus of HSPCs and represses megakaryocytic differentiation by restricting access of megakaryocytic transcription factors to target genes.^55^ Cytokine-mediated phosphorylation of STAT5 therefore triggers two distinct transcriptional consequences: activation of a canonical pSTAT5-driven program that regulates proliferation and apoptosis; and loss of a uSTAT5 program that restrains megakaryocytic differentiation.

Given our limited understanding of the function of STAT5 in HSCs and the complete lack of information about the role of uSTAT5 in primitive HSCs, we explored these issues using genetically modified mice.

## Methods

### Mice

The wild-type C57BL/6 (CD45.2), C57BL/6.SJL (CD45.1) and F1 (CD45.1/CD45.2) mice, and CALR:del mutant mice^58^ in this study were used at 10-32 weeks of age. STAT5^fl/fl^mice^25^ were kindly gifted from Lothar Hennighausen and were crossed with Mx1Cre mice^59^ to generate STAT5^fl/fl^ with Cre (STAT5^fl/fl^Cre^+^) or without Cre (STAT5^fl/fl^Cre^-^). STAT5 deletion was induced with repeated injection with Polyinosinic:polycytidylic acid (Poly:IC). All mice were kept in pathogen free conditions and all procedures were performed according to UK Home Office regulations.

### Smart-seq2 and 10x Genomics single-cell RNA analysis (scRNAseq)

Single ESLAM HSCs were FACS sorted from bone marrow mononuclear cells (BMMNCs) and processed using Smart-seq2 (accession number: GSE223366). Lineage^-^c-Kit^+^ (LK) cells were sorted from BMMNCs and processed using 10xChromium (10xGenomics, Pleasanton, CA; GSE223632). Sorted ESLAM HSCs were transduced with lentivirus containing EV, STAT5B-WT or STAT5B-YF. After a 5-day culture, GFP^+^DAPI^-^ cells were processed using 10xChromium (10xGenomics, Pleasanton, CA; GSE223680). Sorted ESLAM HSCs were cultured for 5 days with ruxolitinib or DMSO, which were then processed using 10xChromium (10xGenomics, Pleasanton, CA; GSE260462). All data were deposited in the National Center for Biotechnology Information (NCBI) Gene Expression Omnibus (GEO).

An IRB/RC approved protocol for human samples.

## Results

### STAT5 loss results in defective HSC function

Previous reports showed that STAT5^-/-^ foetal liver and adult BM cells displayed reduced repopulation in transplantation assays^26,54^ but it was unclear if this was a consequence of reduced HSC number or whether STAT5^-/-^ HSCs are also functionally impaired. We therefore crossed mice carrying a floxed *Stat5a/5b* allele^25^ with Mx1Cre mice and used Poly:IC to delete both *Stat5a* and *Stat5b* loci with ~90% efficiency in haematopoietic cells (Supplemental Figure 1A-C).

**Figure 1:**
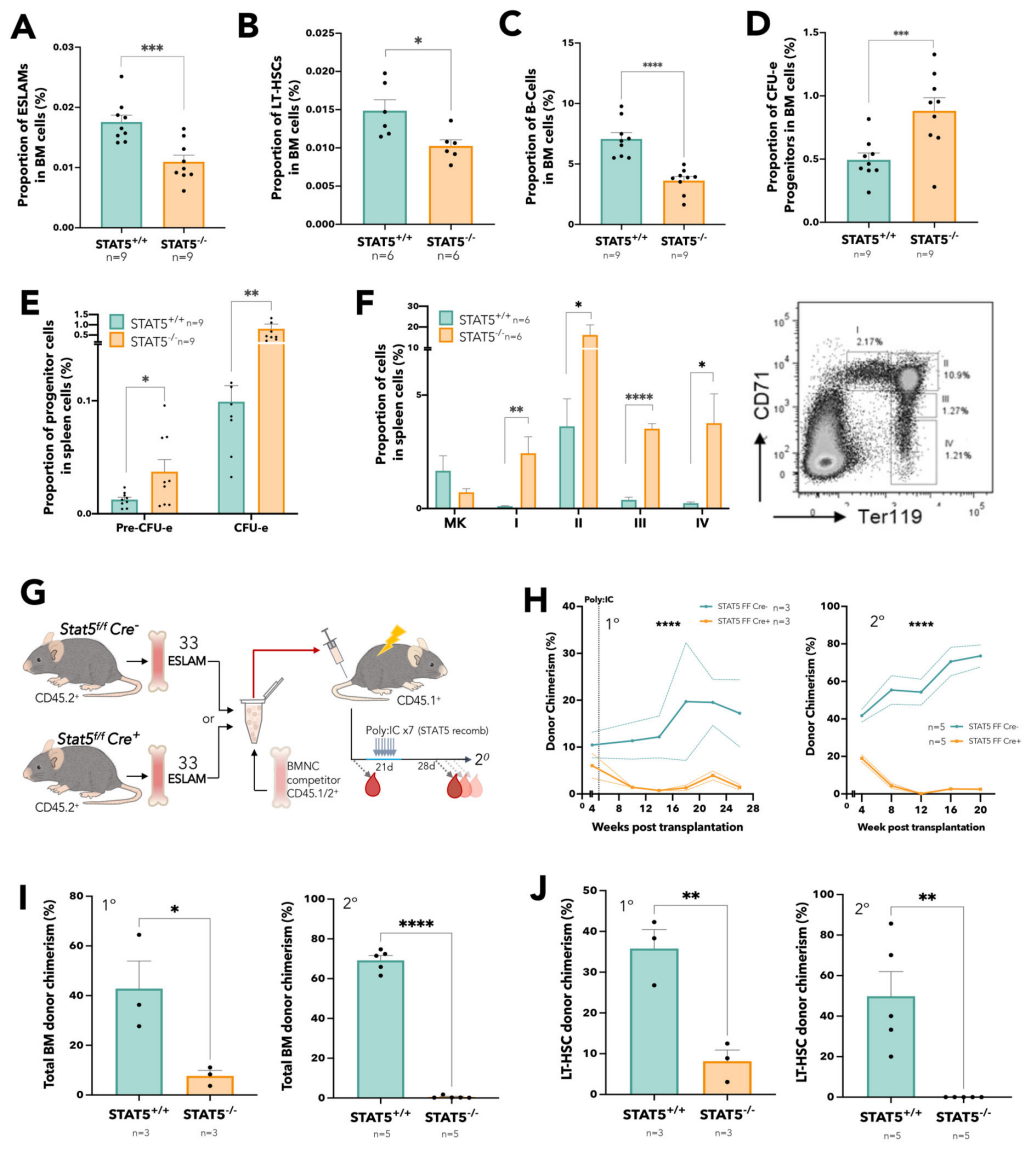
STAT5 loss results in defective HSC function (A) Bar plot showing the frequency of ESLAM HSCs (CD45^+^CD150^+^CD48^-^EPCR^+^) in bone marrow mono-nuclear cells (BMMNCs) from WT and STAT5-deficientmice (mean ± SEM) (B) Bar plot showing the frequency of LT-HSCs (Lin^-^Sca1^-^c-Kit^+^CD150^+^CD48^-^CD34^-^Flk2^-^) in BMMNCs (mean ± SEM). (C) Bar plot showing the frequency of B-cells (B220^+^) in BMMNCs (mean ± SEM). (D) Bar plot showing the frequency of CFU-e progenitors (Lin^-^Sca1^-^cKit^+^CD41^-^CD16/32^-^CD105^+^CD150^-^) in BMMNCs (mean ± SEM). (E) Bar plots showing the frequency of CFU-e and pre-CFU-e (Lin^-^Sca1^-^cKit^+^CD41^-^CD16/32^-^CD105^+^CD150^+^) cells in spleen mono-nuclear cells (mean± SEM). (F) Bar plots (left) showing the frequency of megakaryocyte (CD41^+^CD42^+^) and erythroid precursor cells (I, CD71^hi^Ter119^mid^; II, CD71^hi^Ter119^hi^; III, CD71^mid^Ter119^hi^; IV, CD71^low^Ter119^hi^) in spleen mono-nuclear cells (mean± SEM) with a representative flow cytometry plot (right) showing the gating of different stages of erythroid precursor cells in terminal differentiation. (G) Schematic diagram showing 33 FACS purified BM ESLAM HSCs were transplanted into irradiated recipient mice with 5x10^5^ competitor BMMNCs. STAT5 were deleted in Cre^+^ donor cells after transplantation by repeated injection (x7) with poly:IC in recipients. Blood was taken before and after STAT5 deletion and was followed for 5 months post deletion before serial transplantation of 3x10^6^ primary recipient BMMNCs. (H) Connected line graphs showing donor chimerism in peripheral blood mono-nuclear cells at each time point in primary and secondary recipients (mean ± SEM). Dotted line indicates initiation of Poly:IC injections. Asterisks indicate significant differences by ANOVA column factor (****, p<0.0001). (I) Bar plots showing total BMMNC donor chimerism in primary and secondary recipients (mean ± SEM). (J) Bar plots showing LT-HSC donor chimerism in primary and secondary recipients (mean ± SEM). Asterisks indicate significant differences by Student’s t test (****, p<0.0001; ***, p<0.001; **, p<0.01; *, p<0.05) unless otherwise indicated.

Consistent with previous reports,^25,26^ STAT5 deletion resulted in anaemia, leukopenia and reduced BM cellularity (Supplemental Figures 1D, 1E). In STAT5-deficient BM the frequencies of immunophenotypic primitive HSCs (both ESLAM; Lin^-^CD150^+^CD45^+^CD48^-^EPCR^+^, and LT-HSC; Lin^-^Sca1^+^cKit^+^CD150^+^CD48^-^CD34^-^Flk2^-^; Figure 1A-B) and B-cells (Figure 1C) were reduced and the proportion of erythroid progenitors (CFU-e; Lin^-^Sca1^-^cKit^+^CD41^-^CD16/32^-^CD105^+^CD150^-^) was increased (Figure 1D), but other mature and progenitor cell types were unaltered (Supplemental Figure 1F-I). In the spleen, STAT5 deletion reduced B-cell frequency (Supplemental Figure 1J) and increased frequencies of erythroid progenitors (CFU-e, PreCFU-e; Lin^-^Sca1^-^cKit^+^CD41^-^CD16/32^-^CD105^+^CD150^+^) and all stages of erythroblast differentiation (Figure 1E-F).

Droplet-based (10XGenomics) scRNAseq was performed to assess the haematopoietic stem/progenitor cell (HSPC) landscape. BM LK (Lin^-^cKit^+^) cells from pairs of STAT5^-/-^ and wildtype (WT) control mice were projected onto a previously published LK dataset^60^ and then a phenotypically-defined HSPC dataset,^61^ and cell types were annotated based on their nearest neighbours. Cells within the LT-HSC, ST-HSC, MPP, myeloid, early- and mid-erythroid clusters were relatively reduced in STAT5^-/-^ mice, while the abundance of cells within late-erythroid and lymphoid clusters were relatively increased (Supplemental Figure 1K). These results confirm and extend previous reports and show that STAT5 deficiency causes wide-spread alterations of haematopoietic progenitors including reduced numbers of HSCs.

In competitive transplantation experiments using highly purified ESLAM HSCs (Figure 1G and Supplemental Figure 1L) STAT5-deficient HSCs displayed significantly reduced multilineage repopulation in blood (Figure 1H, Supplemental Figure 1M-O) and BM (Figure 1I, Supplemental Figure 1P) of primary recipients. There was almost no repopulation of blood or BM in secondary recipients. Few or no STAT5-deficient LT-HSCs (Lin^-^Sca1^+^cKit^+^CD150^+^CD48^-^CD34^-^Flk2^-^CD45.1^-^CD45.2^+^) were observed in the BM of primary or secondary recipients respectively (Figure 1J and Supplemental Figure 1Q). These data demonstrate that STAT5-deficient HSCs are not merely reduced in number but are also functionally impaired and display markedly reduced multi-lineage repopulation and self-renewal.

### STAT5-deficient HSCs display reduced cell cycle entry, increased differentiation, and reduced generation of lineage-negative progeny

To explore the molecular basis for HSC dysfunction, plate-based scRNAseq was performed on WT and STAT5-deficient ESLAM HSCs (Supplemental Figures 2A-C) and identified 308 differentially expressed genes (adj. p<0.01, LogFC>±0.5, Supplemental Figure 2D and Supplemental Table 1), including canonical STAT5 targets (eg *Cish, Socs2* and *Bcl6*; Supplemental Figure 2E).

**Figure 2:**
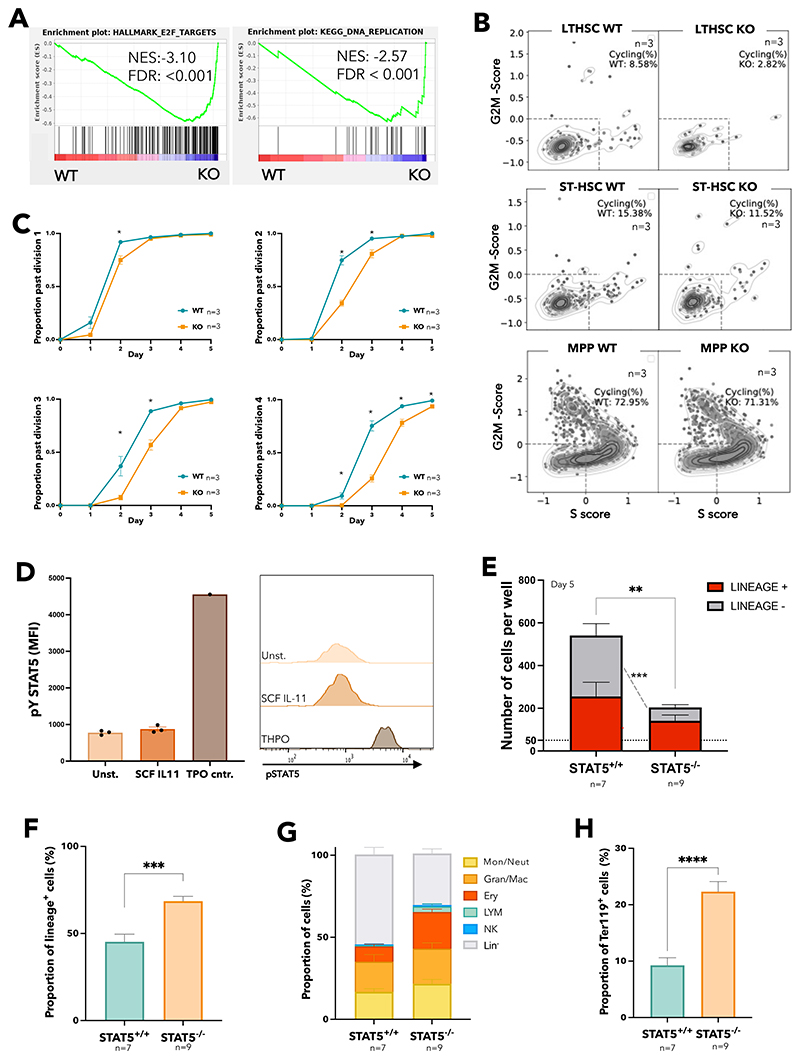
STAT5-deficient HSCs display reduced cell cycle entry, increased differentiation, and reduced retention of lineage-negative progeny. (A) Gene set enrichment analysis (GSEA) plots showing depleted cell cycle related signatures in STAT5-deficient ESLAM (CD45^+^CD150^+^CD48^-^EPCR^+^) HSCs. ScRNAseq analysis using Smartseq platform was performed on FACS isolated ESLAM HSCs from STAT5^f/f^ Cre- or STAT5^f/f^ Cre+ bone marrow, 132 STAT5-deficient and 132 WT HSCs passed quality control and were used for downstream analysis. Normalised enrichment scores (NES) and false discovery rate (FDR) are indicated. (B) Plots showing cell cycle scores of transcriptionally defined LT-HSCs, ST-HSCs and MPPs that were isolated from scRNAseq datasets of WT and STAT5-deficient BM LK cells (STAT5 WT, n=3; STAT5KO, n=3). (C) Line graphs showing proportion of ESLAM HSCs that past first, second, third and fourth divisions at given timepoints (y axis) in single cell *in vitro* analysis (mean ± SEM). Results from 3 biological replicates across 3 experiments. (D) Bar plots (left) showing the mean fluorescent intensity (MFI) of pSTAT5 antibody staining of ESLAM HSCs by intracellular flow-cytometry analysis in unstimulated, maintenance culture conditions^62^ (SCF/IL-11), or TPO (200ng/mL) positive control conditions (mean ± SEM). Right; representative histogram of the intracellular flow-cytometry analysis showing the intensity of pSTAT5 staining in each condition. Results from three biological replicates. (E) Bar plot showing the number of cells in each well at day 5, from an initial culture of 50 ESLAM HSCs in SCF/IL11 maintenance condition. The number of cells expressing mature lineage markers (Ter119^+^, Ly6g^+^, CD11b^+^, NK1.1^+^, B220^+^, CD19^+^ or CD3e^+^) are shaded in red; the number of lineage negative cells are shaded in grey (mean ± SEM). Results from 9-7 biological replicates across 4 experiments. (F) Bar plot showing the proportion of cells expressing mature lineage markers after 5 days in culture originating from 50 ESLAM HSCs (mean ± SEM). (G) Bar plot showing the proportion of cells expressing specific mature lineage markers for monocytes and granulocytes (Ly6g^+^), granulocytes and macrophages (CD11b^+^), erythroid (Ter119^+^), lymphocytes (CD3e^+^/CD19^+^/B220^+^) and natural killer cells (NK1.1^+^) after 5 days in culture originating from 50 ESLAM HSCs (mean ± SEM). (H) Bar plot showing the frequency of Ter119^+^ cells after 5 days in culture originating from 50 ESLAM HSCs (mean ± SEM). Asterisks indicate significant differences by Student’s t test (****, p<0.0001; ***, p<0.001; **, p<0.01; *, p<0.05).

Gene set enrichment analysis (GSEA) identified 12 signatures enriched in STAT5-deficient ESLAM HSCs (FDR<0.25; Supplemental Table 2), including Wnt, Hedgehog and Kras pathways, together with 35 signatures that were depleted (FDR<0.25; Supplemental Table 2), including JAK-STAT signalling, DNA repair and unfolded protein response. The most significantly depleted gene sets were cell cycle related signatures including E2F targets and DNA replication (Figure 2A and Supplemental Figure 2F). Consistent with this observation, analysis of our separate 10X LK cell datasets showed that, compared to WT controls, far fewer STAT5-deficient LT-HSCs were in cycle (8.58 vs 2.82%, Figure 2B). A less pronounced reduction in cell cycling was seen in STAT5-deficient ST-HSCs and MPPs.

Ki-67/DAPI staining showed that, compared to WT mice, STAT5-deficient mice had increased proportions of ESLAM HSCs in G_0_ and reduced proportions in G_1_, although this did not reach statistical significance (Supplemental Figure 2G-2I). However, it is challenging to detect increases in dormancy in populations that are already highly quiescent and Ki-67/DAPI analysis represents a snapshot, which may not capture subtle but relevant changes in quiescence maintenance. We therefore measured the division kinetics of single HSCs (as previously described^62^). STAT5-deficient ESLAM HSCs were indeed slower to enter their first and subsequent divisions (Figure 2C) indicating transient cell cycle arrest or compounded delays in cell cycle entry, thus demonstrating that STAT5 is required for normal HSC cell cycle progression.

The functional consequences of STAT5 deficiency described above reflect the combined effect of losing both pSTAT5 and uSTAT5. To identify those consequences attributable to loss of uSTAT5, experimental conditions that precluded STAT5 phosphorylation were required. We suspected SCF and IL-11 media (previously described to maintain HSCs^62^) would not activate STAT5 phosphorylation in HSCs. Indeed, pSTAT5 levels in ESLAM HSCs cultured in SCF/IL-11 were not significantly higher than cytokine-starved conditions (Figure 2D). After 5 days in this culture condition, STAT5-deficient ESLAM HSCs produced fewer cells overall, with markedly fewer lineage-negative cells (Figure 2E) and an increase in the proportion of lineage-positive cells (Figure 2F). The proportion of each lineage increased (Figure 2G, Supplemental Figure 2J-M), with the erythroid lineage (Ter119^+^) reaching statistical significance (Figure 2H). Similar results were obtained with ESLAM HSCs cultured for 4 or 6 days (Supplemental Figure 2N) with no difference in the frequency of apoptotic cells (Supplemental Figure 2O). These data indicate that loss of uSTAT5 is responsible for increased HSC differentiation and reduced generation of lineage-negative cells.

Together our results therefore demonstrate that STAT5 loss results in an unusual HSC phenotype consisting of reduced cell cycle progression and yet increased differentiation.

### Unphosphorylated STAT5 constrains HSC differentiation and upregulates transcriptional programs associated with HSC maintenance

**Figure 3:**
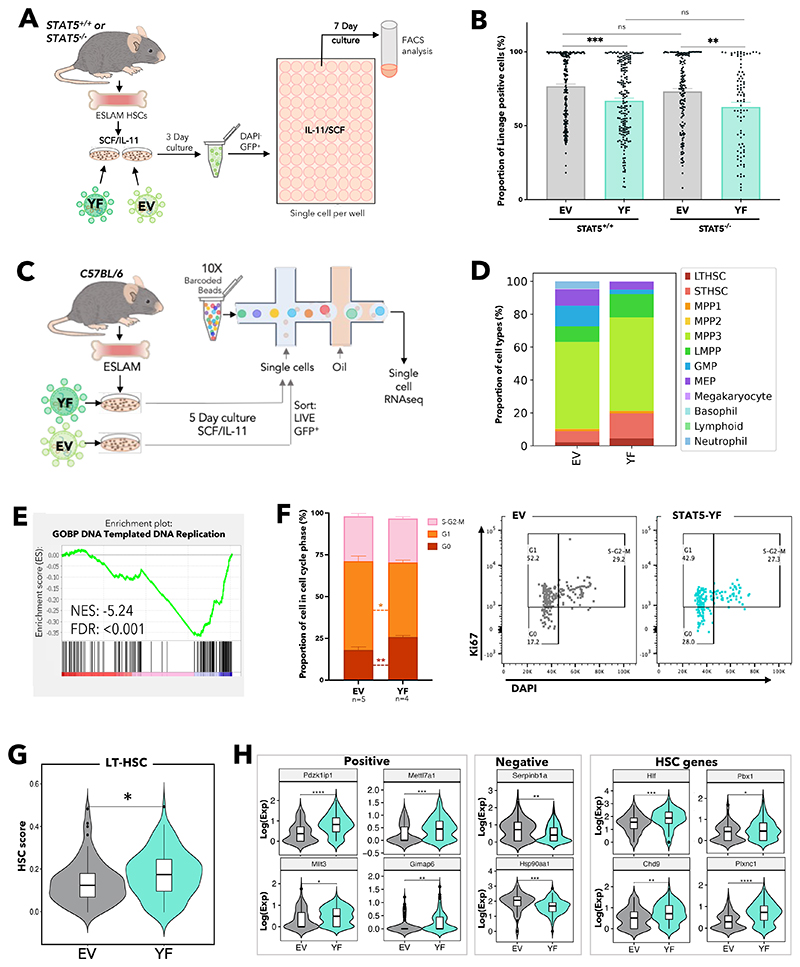
Unphosphorylated STAT5 constrains HSC differentiation and upregulates transcriptional programs associated with HSC maintenance. (A) Schematic diagram showing the experimental outline of the ex vivo functional analysis of STAT5-deficient or WT ESLAM (CD45^+^CD150^+^CD48^-^EPCR^+^) HSCs that were transduced with lentivirus containing STAT5B-Y699F (YF) or EV in maintenance cultures.^62^ After three days of transduction, GFP^+^ living cells were FACS sorted into single cell assays. (B) Bar plots showing the proportion of cells expressing mature lineage markers (Ter119^+^/Ly6g^+^/CD11b^+^/B220^+^/CD3e^+^). Each dot represents a single clone and bars represent mean lineage positive marker frequency (± SEM). Asterisks indicate significant differences by Student’s t test (***, p<0.001; **, p<0.01). Results from 6 to 5 independent biological replicates across 5 experiments in STAT5^+/+^ settings and 4 to 3 independent biological replicates across 3 experiments in STAT5^-/-^ settings. (C) Schematic diagram showing the outline of scRNAseq of WT ESLAM HSCs that were transduced with lentivirus containing STAT5B-Y699F or EV in maintenance cultures^62^ and were allowed to expand for 5 days. GFP^+^ living cells were then sorted for 10X Genomics scRNAseq. (D) Bar plots showing the proportion of annotated cell types in GFP^+^ HSC derived cultures after 5-days in SCF/IL-11 cultures; single cells were projected onto a previously published scRNAseq dataset of LK HSPC cells^60^ and then a phenotypically-defined HSPC dataset,^61^ and cell types were annotated based on their nearest neighbours to ascribe cell identity and cell type annotation. Results from 2 independent biological replicates in 2 experiments. (E) Gene set enrichment plot showing STAT5-YF infected transcriptionally defined LT-HSCs (n=83) are depleted in a DNA replication gene signature compared to EV infected LT-HSCs (n=53). Normalised enrichment score (NES; -5.24), and false discovery rate (FDR; <0.001) are indicated. (F) Left; bar plots showing the cell cycle phase frequency of ESLAM HSC derived cultures infected with either EV (n=5) or STAT5-YF (n=4) lentivirus after 5 days in maintenance culture media.^62^ Cell cycle status was derived from on Ki67/DAPI staining (Right). G_0_ represents quiescent cells that are Ki67^low^DAPI^low^; G_1_ represents cells in early growth phase, which are Ki67^high^DAPI^low^; S-G2-M represents cells in DNA synthesis, late growth, and mitosis stages of active cell cycling and are Ki67^high^DAPI^high^. (G) Violin plot showing the geometric mean distribution of HSC scores in LT-HSCs expressing STAT5B-YF or EV. The HSC score was calculated using the HSC score tool that identifies potential mouse bone marrow HSCs from scRNA-Seq data.^63^ This tool considers the expression of genes that are either positively or negatively corelated with HSC long-term repopulating capacity.^64^ (H) Violin plots showing significantly differentially expressed genes that are positively associated with functional long-term repopulating HSCs (*Pdzk1ip1, Mettl7a1, Mllt3, Gimap1*), negatively associated with functional long-term repopulating HSCs (*Serpinb1a, Hsp90aa1*), or genes with reported functions in maintaining HSCs (*Hlf, Chd9, Pbx1, Plxnc1*). All data is combined from two independent experiments. Asterisks indicate significant differences by Student t test (****, p<0.0001; ***, p<0.001; **, p <0.01; *, p <0.05).

To further explore the role of uSTAT5 in HSCs we utilised a lentiviral expression approach. STAT5B is the dominant form of STAT5 protein in multipotent HPC7 cells^55^ and long-term repopulating HSCs.^52^ STAT5B-Y699F (STAT5-YF), which prevents phosphorylation at this critical residue, was introduced into STAT5^+/+^ or STAT5^-/-^ ESLAM HSCs along with empty-vector (EV) controls (Figure 3A). STAT5-YF and EV constructs showed comparable expansion and survival in STAT5^+/+^ and STAT5^-/-^ HSC-derived clones (Supplemental Figure 3A-B), but STAT5-YF expression resulted in reduced differentiation in STAT5^+/+^ or STAT5^-/-^ clones (Figure 3B). These observations accord with our studies of STAT5^-/-^ HSCs, which indicated that loss of uSTAT5 enhances their differentiation (see above). Thus, both knock-out and over-expression approaches indicate that uSTAT5 constrains HSC differentiation. STAT5-YF expression increased total STAT5 levels 2-3-fold in LSKs (Supplemental Figure 3C-D) and so our results indicate that the functional consequences of STAT5-YF reflect a 2-3-fold increase in uSTAT5, given that the vast majority of potentially phosphorylatable STAT5 (ie can be phosphorylated by THPO) remains unphosphorylated in SCF/IL-11^62^ media (Supplemental Figure 3E).

The transcriptional consequences of STAT5-YF expression in ESLAM HSCs were explored using 10X Genomics scRNAseq (Figure 3C). Since STAT5^-/-^ and STAT5^+/+^ HSCs responded similarly to STAT5-YF overexpression and STAT5-deficient HSCs are less abundant, STAT5^+/+^ HSCs were used for this analysis. *Stat5b* transcripts increased 2-fold in STAT5-YF infected cells (Supplemental Figure 3F) consistent with protein levels (Supplemental Figure 3B). Infected cells were projected onto a previously published scRNAseq dataset of LK cells^60^ and then a phenotypically-defined HSPC dataset,^61^ and cell types were annotated based on their nearest neighbours. Compared to control EV cultures, STAT5-YF cultures contained fewer differentiated cell types (eg GMPs, MEPs, and neutrophils) but more early stem/progenitor cells (LT-HSCs and ST-HSCs; Figure 3D and Supplemental Figure 3G). These results accord well with our functional evidence that STAT5-YF constrains differentiation.

Within transcriptionally defined LT-HSCs, STAT5-YF expression was associated with upregulation/downregulation of 321/120 genes respectively (Supplemental Table 3), representing both direct and indirect consequences of STAT5-YF expression. Expression levels of target genes for pSTAT5 (*Pim1, Ccnd1, Mcl1*, and *Sod2*) were unaffected by STAT5-YF expression (Supplemental Figure 3H) and GSEA failed to identify enrichments or depletions in canonical STAT5-target gene sets (data not shown), indicating that STAT5-YF is not exerting a dominant negative effect. Consistent with this concept, the vast majority of phosphorylatable STAT5 remains unphosphorylated in HSCs cultured in SCF/IL11 (Figure 2D and Supplemental Figure 3E), although our results cannot completely exclude the existence of very low levels of pSTAT5 below our detection levels.

In HPC7 cells we have previously shown that uSTAT5 repressed several megakaryocytic genes (*Mpl, Vwf, Gp9, F2r*) and that it competed with ERG in regulating *Mpl* and *F2r*. We therefore explored whether similar effects could be found in highly purified HSCs. Expression levels of *Mpl, Vwf, Gp9* and *F2r* were not increased in STAT5^-/-^ HSCs or reduced in STAT5-YF-expressing HSCs (Supplementary Figure 3I), which likely reflect different transcriptional programmes within HPC-7 cells (similar to MEPs and derived from embryonic stem cells) and HSCs.

Cell cycle gene signatures were significantly depleted in STAT5-YF infected LT-HSCs (Figure 3E and Supplemental Figure 3J), more STAT5-YF LT-HSCs were in G_0_/G_1_ phases (Supplemental Figure 3K) and Ki67/DAPI analysis in STAT5^+/+^ ESLAM-derived cultures confirmed that STAT5-YF expression increased the frequency of HSCs in G_0_ (Figure 3F), collectively indicating that STAT5-YF expression is associated with increased HSC quiescence. STAT5-YF expressing LT-HSCs exhibited higher HSC scores compared to EV expressing HSCs (Figure 3G) using a previously described algorithm that identifies durable long-term repopulating HSCs^63^ and takes into account the expression of genes that correlate either positively or negatively with HSC function.^64^ STAT5-YF HSCs also exhibited higher HSC scores using two other published HSC signatures (Supplemental Figure 3L).^65,66^ Indeed, positively-associated HSC-score genes were upregulated in STAT5-YF LT-HSCs, while anti-correlated genes were downregulated, and other genes reported to promote HSC maintenance were also upregulated (Figure 3H).

Together our data therefore demonstrate that STAT5-YF restrains HSC differentiation, increases HSC quiescence and regulates transcriptional networks associated with increased HSC maintenance.

### Unphosphorylated STAT5 enhances HSPC clonogenicity *in vitro* and HSC maintenance *in vivo*

**Figure 4:**
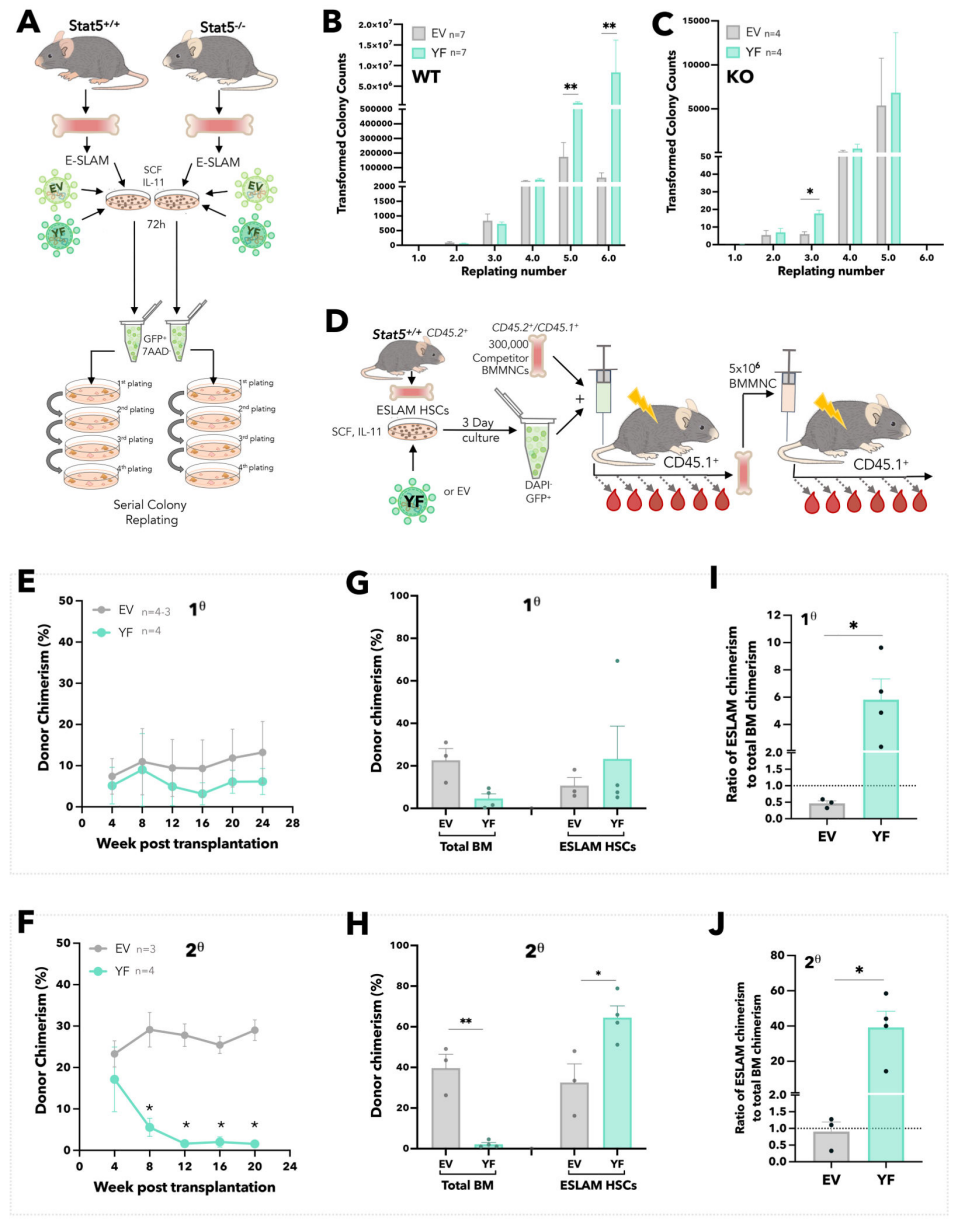
Unphosphorylated STAT5B enhances HSPC clonogenicity *in vitro* and HSC maintenance *in vivo*. (A) Schematic diagram showing the experimental outline of serial colony replating assays of STAT5-deficient or WT ESLAM (CD45^+^CD150^+^CD48^-^EPCR^+^) HSCs that were transduced with lentivirus containing STAT5B-Y699F (YF) or EV in SCF/IL-11 maintenance cultures. After three days of transduction, GFP^+^ living cells were sorted for serial colony replating assays. (B) Bar plots showing the transformed colony numbers derived from WT HSPCs transduced with YF or EV lentivirus. Transformed colony counts = ((colony number x dilution factor) ÷ starting number of HSCs)) (mean ± SEM). Results were from four independent experiments and 7 biological replicates. Asterisks indicate significant differences by Mann-Whitney t test (**, p<0.01). (C) Bar plots showing the transformed colony numbers of STAT5-deficient HSPCs transduced with YF or EV lentivirus. Transformed colony counts = ((colony number x dilution factor) ÷ starting number of HSCs)) (mean ± SEM). Results were from three independent experiments and 4 biological replicates. Asterisks indicate significant differences by Mann-Whitney t test (*, p<0.05). (D) Schematic diagram showing outline of the *in vivo* functional analysis of WT ESLAM HSCs that were transduced with lentivirus containing STAT5B-Y699F (YF) or EV. FACS sorted WT ESLAM HSCs (CD45.2^+^) were infected with lentivirus and cultured for 3 days in maintenance cultures, then equal number of GFP^+^ cells were FACS sorted (112 GFP^+^ cells/recipient) and injected into irradiated recipients (CD45.1^+^) with 3x10^5^ competitor BMMNCs (CD45.1^+^/CD45.2^+^). Donor chimerism was monitored every 28 days for over 6 months. Secondary transplantation was then performed using 5x10^6^ BMMNCs from primary recipients. BMMNCs from one primary recipient were transplanted into up to 2 recipients. (E) Connected line graph showing donor chimerism in primary recipients (mean ± SEM); experiment described in 4D. Chimerism was derived as the ratios of donor/(donor+competitor). (F) Connected line graph showing donor chimerism in secondary recipients (mean ± SEM); experiment described in 4D. Chimerism was derived as the ratios of donor/(donor+competitor). (G) Bar plots showing donor chimerism in total BMMNCs and ESLAM HSCs in BM of the primary transplant recipients (mean ± SEM). (H) Bar plots showing donor chimerism in total BMMNCs and ESLAM HSCs in BM of the secondary transplant recipients (mean ± SEM). (I) Bar plots showing the ratio of ESLAM HSC donor chimerism to total BMMNC chimerism in primary recipient BM (mean ± SEM; dotted line indicating 1:1 ratio). (J) Bar plots showing the ratio of ESLAM HSC donor chimerism to total BMMNC chimerism in secondary recipient BM (mean ± SEM; dotted line indicating 1:1 ratio). Chimerism was derived as the ratios of donor/(donor+competitor). Asterisks indicate significant differences by Student’s t test (**, p<0.01; *, p<0.05).

We next explored the effect of STAT5-YF expression on HSC function (Figure 4A). In serial colony replating assays STAT5^+/+^ HSCs expressing STAT5-YF displayed enhanced colony generation in 4 independent experiments (Figure 4B and Supplemental Figure 4A) demonstrating that uSTAT5 is sufficient to enhance generation of clonogenic progeny by WT HSCs. Introduction of STAT5-YF had no effect on the replating of STAT5^-/-^ HSCs, but these cells produced far fewer colonies for a shorter duration than WT cells (Figure 4C and Supplemental Figure 4B) indicating a requirement for pSTAT5 in the replating assay, likely through its role in driving proliferation.^55^ Indeed, STAT5 phosphorylation was readily detectable in HSCs cultured in the replating assay media which contained IL-3 and IL-6 (Supplemental Figure 4C).

In competitive transplantation experiments (Figure 4D), compared to control EV-infected HSCs, those carrying STAT5-YF generated peripheral blood donor chimerism that was modestly reduced in primary recipients (Figure 4E) and dramatically reduced in secondary recipients (Figure 4F, Supplemental Figure 4D). Furthermore, compared to control EV-infected HSCs, HSCs infected with STAT5-YF gave rise to reduced total BM chimerism but increased ESLAM-HSC chimerism in primary recipients (Figure 4G) an observation that was even more striking in secondary recipients (Figure 4H). Within individual primary recipients, the ratio of HSC chimerism to total BM chimerism was substantially higher for mice that had received STAT5-YF HSCs compared to those that had received EV HSCs (Figure 4I). This pattern was even more striking in secondary transplant recipients (Figure 4J).

Together our results therefore indicate that STAT5-YF expression enhances the clonogenicity of HSCs *ex vivo* and increases HSC chimerism while restricting their repopulating capacity *in vivo*.

### Ruxolitinib enhances HSPC clonogenicity and maintains transplantable HSCs

**Figure 5:**
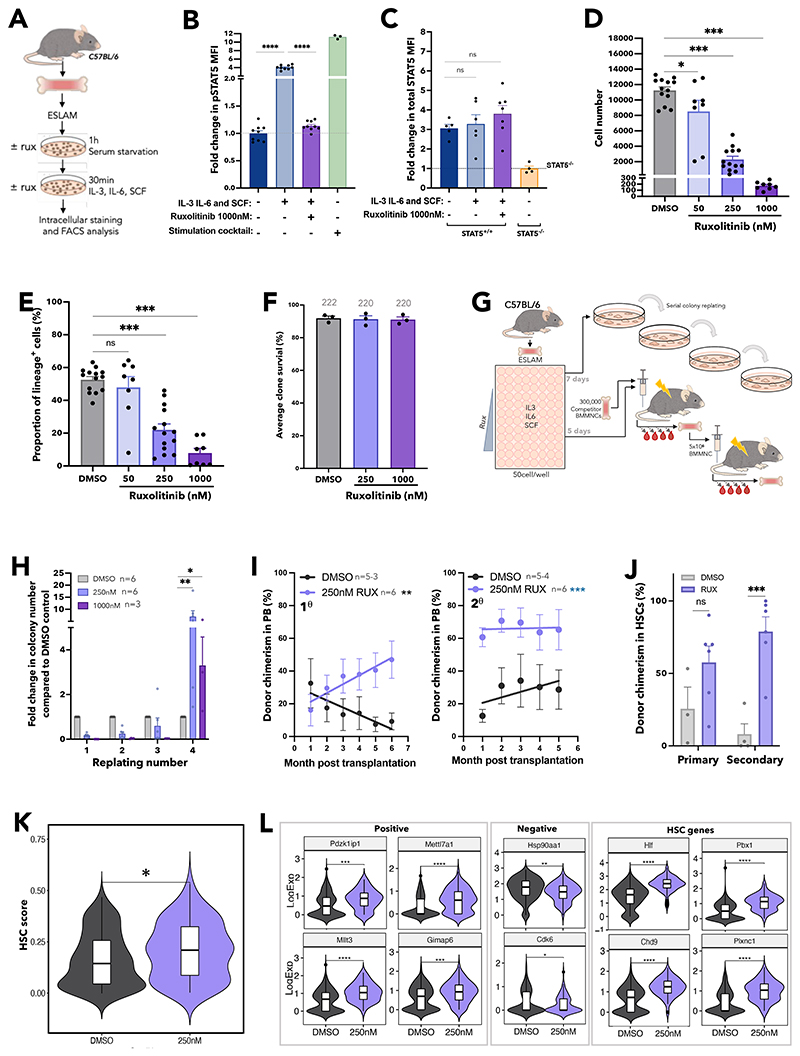
Ruxolitinib enhances HSPC clonogenicity and maintains transplantable HSCs. (A) Schematic diagram showing intracellular flow cytometric analysis of STAT5 proteins in ruxolitinib (RUX) treated WT ESLAM HSCs (CD45^+^CD150^+^CD48^-^EPCR^+^). WT ESLAM HSCs were sorted into serum starved media and starved for 1h before a 30-minute stimulation with complete medium containing IL-3, IL-6 and SCF in the presence or absence of RUX^90^, a stimulation cocktail containing THPO, Flt3-L and IFNα for positive control was included. Cells were then fixed and stained for intracellular flow cytometry. (B) Bar plots showing the mean fluorescent intensity (MFI) of pSTAT5 antibody staining in ESLAM HSCs described in ‘A’ normalised to the unstimulated condition which is indicated with dotted line (mean ± SEM). Each dot represents the mean fluorescent intensity of ESLAMs from a single mouse. Results from 3 independent experiments. (C) Bar plots showing the mean fluorescent intensity of total-STAT5 (tSTAT5) antibody staining in ESLAM HSCs, described in ‘A’ normalised to STAT5-deficient HSPCs which is indicated with dotted line (mean ± SEM). Results from 3 independent experiments. (D) Bar plots showing cell number per well in HSC derived cultures at each dose of ruxolitinib or vehicle after 7 days (mean ± SEM). 50 ESLAMs were seeded per well in 96-well plates in IL-3/IL-6/SCF^90^ cultures and were treated with DMSO or indicated doses of ruxolitinib. Results from 6 independent experiments. (E) Bar plot showing the proportion of cells expressing lineage positive markers (Ter119^+^/Ly6g^+^/CD11b^+^/B220^+^/CD3e^+^) after 7 days in culture at different concentrations (nM) of ruxolitinib (mean ± SEM). 50 ESLAMs were seeded per well in 96-well plates in IL-3/IL-6/SCF^90^ cultures and were treated with indicated doses of ruxolitinib. Results from 6 independent experiments. (F) Bar plots showing the clone survival rate of single HSCs after 5 days in culture. Single ESLAM HSCs were sorted per well and treated with vehicle or ruxolitinib, Clone survival rate was the proportion of wells that contained cells at day 5. Each dot represents the frequency of surviving clones from each of three independent experiments; bars show the mean ± SEM. (G) Schematic diagram showing serial colony replating assays and *in vivo* functional analysis for ESLAM HSCs treated with RUX or vehicle. 50 WT ESLAM HSCs were sorted per well into complete media^90^ with scaled doses of ruxolitinib or vehicle. Cells were harvested after 7 days and plated into serial colony replating assays. ESLAM HSC (CD45.2^+^) derived cells after 5 days in culture were harvested and transplanted into lethally irradiated recipient mice (CD45.1^+^) with 3x10^5^ fresh BMMNCs from competitor mice (CD45.1^+^/CD45.2^+^). Blood was analysed every 28 days for 6 months. Secondary transplants were then set up by transplanting 3x10^6^ bone marrow cells from the primary transplants recipients. (H) Bar plots showing the number of colonies produced by HSC-derived cultures treated with vehicle or ruxolitinib (250nM or 1000nM) for 7 days, normalised to the number of colonies produced by vehicle treated cultures at each week of replating. Results are shown as mean ± SEM and were from five independent experiments, three of which included 1000nM. Asterisks indicate significant differences by Mann-Whitney t test (**, p<0.01; *, p<0.05). (I) Scatter dot plot with linear regression line of best fit showing the peripheral blood donor chimerism in primary (left) and secondary (right) recipients transplanted with 5-day *ex vivo* cultured HSCs with ruxolitinib or vehicle. 50 ESLAMs from WT mice were seeded per well in IL-3/IL-6/SCF culture conditions and given DMSO or 250nM of ruxolitinib for 5 days before the cells were harvested and pooled for each condition and an equivalent of 10 starting ESLAMs was transplanted per recipient with 3x10^5^ competitor bone marrow cells. Each dot indicates mean donor chimerism and are shown as mean ± SEM. Black asterisks indicate significant differences in the slopes of linear regression modelling comparing chimerism of ruxolitinib treated donor cell to DMSO treated donor cell chimerism in the primary recipients (**, p<0.01). Blue asterisks indicate significant differences in y-intercepts of linear regressions modelling comparing chimerisms of ruxolitinib treated donor cell compared to DMSO treated donor cell in secondary transplants (***, p<0.001). (J) Bar plots showing the donor chimerism within the ESLAM HSC compartment at the end of primary and secondary recipients of 5-day *ex vivo* cultured HSCs with ruxolitinib or vehicle. Data are shown as mean ± SEM. (K) Violin plot showing the geometric mean distribution of HSC scores in LT-HSCs from 10x scRNAseq dataset of the cells treated with ruxolitinib or DMSO. The scores were calculated using the HSCscore tool that identifies potential mouse bone marrow HSCs from scRNA-Seq data.^63^ This tool considers the expression of genes that are either positively or negatively correlated with HSC long-term repopulating capacity.^64^ (L) Violin plots showing significantly differentially expressed genes that are positively associated with functional long-term repopulating HSCs (*Pdzk1ip1, Mettl7a1, Mllt3, Gimap6*), negatively associated with functional long-term repopulating HSCs (*Hsp90aa1, Cdk6*), or genes with reported functions in maintaining HSCs (*Hlf, Pbx1, Chd9, Plxnc1*). All data is combined from two independent experiments. Asterisks indicate significant differences by Student’s t test (****, p<0.0001; ***, p<0.001; **, p<0.01; *, p<0.05) unless otherwise indicated.

JAK inhibitors, such as ruxolitinib, are predicted to increase the ratio of uSTAT5 to pSTAT5. Indeed, ruxolitinib treatment of cells with activated JAK/STAT signalling (driven by mutant JAK2 or mutant CALR), resulted in a dramatic reduction in pSTAT5 without a fall in total STAT5 protein levels (Supplemental Figure 5A). In ESLAM HSCs levels of pSTAT5 (but not pSTAT1 or pSTAT3), were induced 4-fold in a ruxolitinib-sensitive manner when exposed to SCF, IL3 and IL6 (Figure 5A, 5B and Supplemental Figure 5B-5C). Total STAT5 protein levels were unchanged (Figure 5C) and pSTAT5 target genes such as *Cish* and *Pim1* were downregulated in HSCs exposed to ruxolitinib (Supplemental Figure 5D).

Ruxolitinib reduced, in a dose-dependent manner, the progeny generated by ESLAM HSCs (Figure 5D, Supplemental Figure 5E-5F) and the proportion of lineage-positive cells (Figure 5E, Supplemental Figure 5G-5H). Two other JAK inhibitors, fedratinib and momelotinib, similarly reduced the expansion and differentiation of ESLAM HSCs in culture (Supplemental Figure 5I-5J). Treatment with ruxolitinib was not accompanied by reduced HSPC viability; even single ESLAM HSCs cultured with high doses of ruxolitinib (eg 1000nM, well above the therapeutic range) showed no difference in the proportion of wells containing one or more viable cells at 5 days (Figure 5F). Moreover, treatment of lineage-depleted BM cells with ruxolitinib over-night resulted in apoptosis of mature cell types but had little effect on LK cells suggesting that ruxolitinib does not affect survival of early HSPCs (Supplemental Figure 5K).

To investigate the effect of ruxolitinib on HSC function, serial colony replating assays and competitive transplants were performed (Figure 5G). Compared to vehicle-treated HSCs, those exposed to ruxolitinib formed significantly more colonies in the final week of replating assays (Figure 5H, Supplemental Figure 5L-5M) indicating that ruxolitinib increased maintenance of clonogenic HSPCs in precultures. In two independent competitive repopulation experiments, vehicle-treated control cells gave rise to donor peripheral blood chimerism that gradually fell over the 5-month study period (Figure 5I, Supplemental Figure 5N) as previously reported for cultured HSC donors.^67^ In marked contrast ruxolitinib-treated HSCs gave rise to levels of donor peripheral blood chimerism that were initially lower than controls and then were maintained or increased. In secondary recipients, donor HSCs originally treated with ruxolitinib displayed significantly higher peripheral blood chimerism (Figure 5I, Supplemental Figure 5O). Moreover, primary recipient mice that had received HSCs precultured with ruxolitinib displayed increased ESLAM-HSC chimerism, an effect that was even more marked in secondary recipients (Figure 5J).

Single cell RNAseq was used to explore the transcriptional consequences of ruxolitinib (Supplemental Figure 5P). Ruxolitinib-treated ESLAM HSC-derived cultures exhibited reduced expression of canonical pSTAT5 target genes (Supplemental Figure 5Q) and contained more transcriptionally defined LT-HSCs and ST-HSCs (Supplemental Figure 5R). Compared to control LT-HSCs, ruxolitinib treated LT-HSCs were depleted in cell-cycle gene signatures (Supplemental Figure 5S) and possessed a greater frequency of cells in G_0_/G_1_ (Supplemental Figure 5T). These data were confirmed by Ki67/DAPI analysis at 18 hour and 5-day timepoints (Supplemental Figure 5U-V), collectively showing that ruxolitinib promotes HSC quiescence *ex vivo*.

Consistent with their increased quiescence, ruxolitinib-treated LT-HSCs showed increased HSC-fitness scores (Figure 5K, Supplemental Figure 5W) using the 3 different published scoring methods^63,65,66^ that had also demonstrated increased HSC scores for STAT5-YF treated LT-HSCs (Figure 3G, Supplemental Figure 3J). Ruxolitinib-treated LT-HSCs also showed increased scores for a signature derived by comparing STAT5-YF expressing LT-HSCs to EV-transduced controls (Supplemental Figure 5X). Furthermore, ruxolitinib increased the expression of positively-associated HSC-score genes, reduced the expression of negatively-associated HSC-score genes and increased the expression of multiple other genes associated with HSC maintenance (Figure 5L) in a manner similar to STAT5-YF expression (Figure 3H). Several of these genes (eg *Pdzk1ip1, Gimap6, Hlf, Plxnc1* and *Chd9*) had previously been identified by ChIP studies^55^ as direct targets of uSTAT5 (Supplemental Figure 5Y).

Together our data demonstrate that ruxolitinib pre-treatment reduced HSC differentiation, increased HSC quiescence and enhanced the maintenance of transplantable HSCs during *ex vivo* culture. Moreover, the transcriptional consequences of ruxolitinib closely paralleled those observed for STAT5-YF expressing HSCs (Figure 3G-H) indicating that the effects of ruxolitinib are mediated, at least in part, by uSTAT5.

### Ruxolitinib maintains murine and human myeloproliferative neoplasm HSPCs

Ruxolitinib alleviates symptoms, reduces splenomegaly and modestly extends overall survival in a subset of MPN patients with advanced disease.^47–49^ However it has little or no effect on allele burden and disease progression^48,49^ suggesting that ruxolitinib does not eradicate malignant HSCs. This has been attributed to ruxolitinib having a narrow therapeutic window as a consequence of dose limiting toxicity.^68,69^ However our data raise the possibility that JAK inhibitors might also inherently promote maintenance of mutant HSCs by increasing levels of uSTAT5.

**Figure 6:**
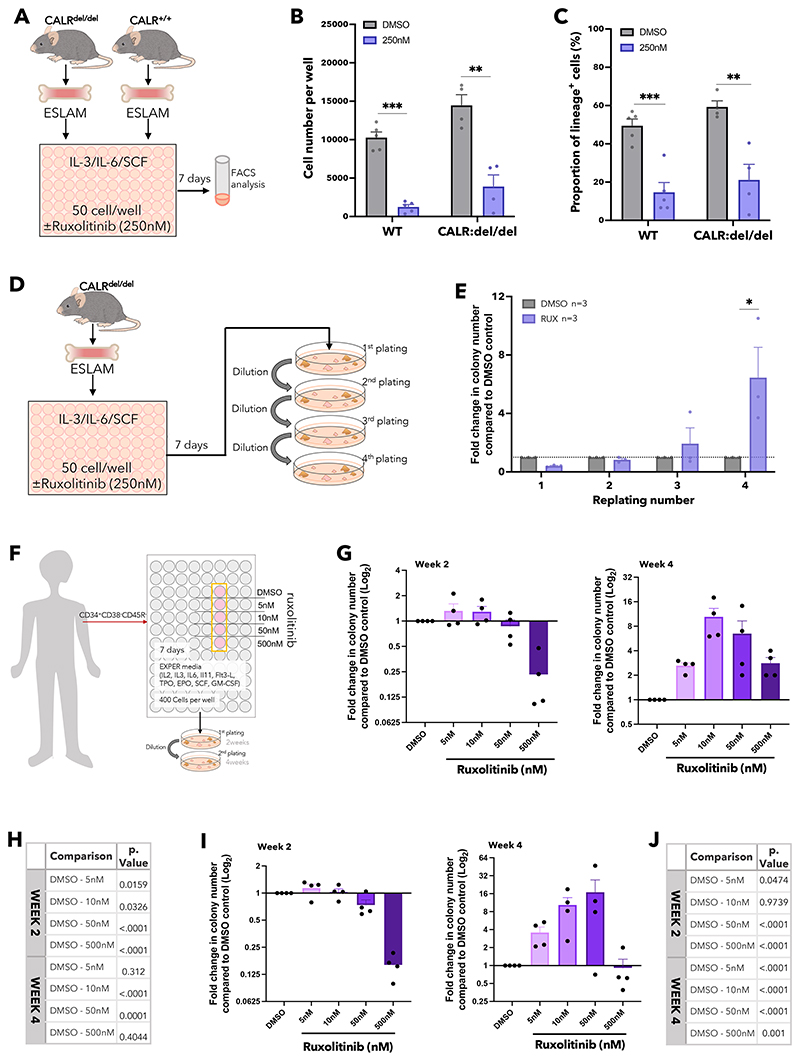
Ruxolitinib maintains murine and human myeloproliferative neoplasm HSPCs. (A) Schematic diagram showing *in vitro* functional assays of murine ESLAM HSCs (CD45^+^CD150^+^CD48^-^EPCR^+^) treated with RUX or DMSO. ESLAM HSCs were FACS isolated from CALRdel/del (n=4) mutant mice and were then cultured for 7 days in IL-3/IL-6/SCF media^90^ with DMSO or 250nM of ruxolitinib before analysis by flow-cytometry. (B) Bar plots showing cell number per well in HSC derived cultures treated with vehicle or 250nM of ruxolitinib after 7 days (mean ± SEM). (C) Bar plots showing the proportion of cells expressing lineage positive markers (Ter119^+^/Ly6g^+^/CD11b^+^/B220^+^/CD3e^+^) after 7 days in culture with DMSO or 250nM of ruxolitinib (mean ± SEM). Asterisks indicate significant differences by Student’s t test (***, p<0.001; **, p<0.01; *, p<0.05). (D) Schematic diagram showing serial replating assays investigating the effect of Rux on ESLAM HSCs isolated from WT and CALRdel/del mutant mice. Sorted ESLAM HSCs were cultured for 7 days in IL-3/IL-6/SCF media^90^ with DMSO or 250nM of ruxolitinib and then subjected to serial colony replating assays. (E) Bar plots showing the fold change in number of colonies produced by HSC-derived cultures treated with vehicle or 250nM ruxolitinib for 7 days, normalised to the number of colonies produced by vehicle treated cultures at each week of replating. Results are from 2 independent experiments and are shown as mean ± SEM. Asterisks indicate significant differences by Mann-Whitney t test (*, p<0.05) (F) Schematic diagram showing HSCs (MPP1-LTHSCs; CD34^+^CD38^-^CD45RA^-^) cells were sorted from healthy human platelet apheresis donor cone samples, or myelofibrosis patient peripheral blood, into 96 well plates (400 cells/well) and cultured in high cytokine, serum-free medium (EXPER cytokine media)^71^ with scaled doses of ruxolitinib or vehicle control (DMSO). After 7 days the HSC derived cultures were plated in serial colony replating assays in methylcellulose. Healthy donors were all male and between 48-69 years of age. Myelofibrosis patient donors; three patients carried a JAK2 V617F mutation and were all male between the ages of 65-70, one donor carried a CALR-52bp deletion mutation and was a 70y/o female at the time of sample collection. (G) Bar plots showing the fold change in the number of colonies produced by HSPCs that were isolated from healthy donors and cultured for 7 days in the presence of ruxolitinib, normalised to the number of colonies produced by HSPCs cultured for 7 days with DMSO. Data shown as Log_2_(fold-change) from DMSO. Left showing fold change in colony numbers in the first round of colony formation (2 weeks in methylcellulose). Right showing fold change in colony numbers in the second round of colony formation (4 weeks in methylcellulose). Data from 4 healthy donors; each dot represents the mean fold change between technical replicates of a single donor. (H) Table showing the significance values (p.value) from estimated marginal (EM) means statistics derived from comparisons between DMSO and ruxolitinib conditions using a generalised mixed linear model applied to the raw colony counts used to generate 5G. (I) Bar plots showing the fold change in number of colonies produced by HSPCs that were isolated from patients with myelofibrosis and cultured for 7 days in the presence of ruxolitinib, normalised to the number of colonies produced by HSPCs cultured for 7 days with DMSO. Data shown as Log_2_(fold-change) from DMSO. Left showing fold change in colony numbers in the first round of colony formation (2 weeks in methylcellulose). Right showing fold change in colony numbers in the second round of colony formation (4 weeks in methylcellulose). Each dot represents the average fold-change from each of 4 patients with myelofibrosis and bars represent mean ± SEM. (J) Table showing the significance values (p.value) from EM means statistics derived from comparisons between DMSO and ruxolitinib conditions DMSO and ruxolitinib conditions using a generalised mixed linear model statistic applied to colony counts used to generate 5I.

We therefore studied the effect of ruxolitinib on CALR-mutant HSCs derived from a knock-in mouse model^58^ carrying a CALR-52bp deletion mutation commonly observed in human MPN patients^43^ and known to activate JAK/STAT signalling^70^ (Figure 6A). Ruxolitinib reduced the number of progeny generated by CALR-mutant ESLAM HSCs and also the proportion of lineage-positive cells (Figure 6B, 6C). Ruxolitinib pre-treatment also enhanced the replating capacity of cells derived from CALR-mutant ESLAM HSCs (Figure 6D, 6E, Supplementary Figure 6A, 6B) demonstrating that ruxolitinib maintains clonogenic HSPCs.

To investigate whether ruxolitinib maintained human HSCs in *ex vivo* cultures, CD34^+^CD38^-^CD45RA^-^ HSPCs were purified from apheresis cones derived from 4 platelet donors, grown in cytokine rich, serum-free culture conditions^71^ with or without ruxolitinib and their progeny assessed in serial colony replating assays (Figure 6F). These human cell cultures did not contain albumin, which binds ruxolitinib, necessitating the use of lower ruxolitinib doses as previously described.^72^ After 2 weeks, ruxolitinib did not increase colony formation and even reduced colony output at the highest dose (500nM), but by 4 weeks it increased colony formation in all individuals at all doses tested, with 10nM and 50nM (similar to concentrations obtained in patients *in vivo*^73^ after accounting for albumin) showing the greatest benefit (Figure 6G-6H, Supplementary Figure 6C-6E and Supplemental Table 5).

Ruxolitinib had a similar effect on HSPCs (CD34^+^CD38^-^CD45RA^-^) derived from the peripheral blood of 4 patients with myelofibrosis with high WBC counts, none of whom had previously received ruxolitinib or interferon. Three patients were positive for the JAK2V617F mutation and one patient had a CALR-deletion mutation. After 2 weeks, ruxolitinib had little effect on colony output except at the highest dose, but at 4 weeks it substantially increased colony output in all 4 patients, with 10nM and 50nM concentrations showing the greatest benefit (Figure 6I-J, Supplemental Figure 6F-6H and Supplemental Table 6).

Together, these data demonstrate that ruxolitinib maintained cultured murine myeloproliferative HSCs, human normal HSPCs and human myeloproliferative HSPCs.

## Discussion

Our results demonstrate that STAT5 loss is accompanied not only by reduced HSC numbers but also a substantial HSC impairment associated with reduced cell cycle entry and increased differentiation. Prompted by this unusual phenotype, we show that uSTAT5 promotes maintenance and constrains differentiation and proliferation of HSCs. Ruxolitinib, a JAK1/2 inhibitor in wide clinical use, increases uSTAT5 levels and enhances maintenance of WT and myeloproliferative HSCs from both mice and humans.

An intimate relationship between proliferation and differentiation has long been recognised in studies of HSC biology. Many genetic (e.g. ablation of CDKi^74–76^ or MEK1^77^) or environmental manipulations (e.g. infections or inflammation^78–81^) that induce HSC proliferation and functional exhaustion are associated with increased differentiation.^78–81^ In contrast, many of those that produce increased HSC quiescence are accompanied by reduced differentiation (e.g. Neo1 downregulation^82^ or Atad3a deletion^83^). However, we show here that highly purified STAT5-deficient HSCs display transcriptional evidence of reduced cell cycling together with functional evidence of reduced cell cycle entry, and yet are more prone to differentiation. Bunting and colleagues have previously reported that STAT5-deficient LSK or CD34^-^LSK HSCs displayed increased cell cycling.^26,51^ However, the frequencies of quiescent cells in their WT control populations were lower than those observed in ESLAM HSCs here (84% vs 91%), suggesting that cell populations gated for cell cycle analysis in the previous reports contained a higher frequency of more proliferative progenitors (presumably ST-HSC/MPP). The decreased frequency of primitive HSCs in STAT5^-/-^ mice likely resulted in a higher fraction of more proliferative ST-HSC/MPPs, thus increasing proliferation scores for populations containing such cells.

Our demonstration that STAT5 not only induces HSC proliferation but also represses HSC differentiation was reminiscent of previous results, which showed that uSTAT5 and pSTAT5 have separate transcriptional roles in megakaryocytic differentiation of multipotent HPC7 cells.^55^ We therefore explored the possibility that the functional consequences of STAT5 loss in HSCs might represent a compound phenotype involving loss of both uSTAT5 and pSTAT5 transcriptional programs. Two aspects of our studies are of particular note.

First, our results indicate that uSTAT5 constrains HSC differentiation (as shown by both knock-out and lentiviral expression approaches), HSC proliferation and also enhances HSC maintenance as assessed by serial replating and transplantation of STAT5-YF expressing cells. In the latter studies, STAT5-YF increased donor chimerism within the HSC compartment in both 1^0^ and 2^0^ recipients but reduced donor chimerism within whole bone marrow, indicating that STAT5-YF expressing HSCs are retained in the HSC compartment and are less likely to differentiate. Second, these functional changes reflected altered HSC transcriptional programs including signatures of reduced differentiation, increased quiescence and increased stemness as assessed by several different scoring systems.

Our results highlight the need to take the signalling environment into account when interpreting the consequences of manipulating a STAT. Thus, using culture conditions that preclude significant STAT5 phosphorylation, the consequences of up or down-regulating STAT5 can be attributed to an effect on uSTAT5. However, we cannot completely exclude potential confounding effects of low levels of endogenous pSTAT5 when assessing the effect of STAT5-YF expression in wild-type HSCs. Our results also underline the challenges inherent in disentangling the different biological effects of uSTAT5 and pSTAT5. For example, a requirement for pSTAT5 in replating assays precluded analysis of the ability of STAT5-YF to rescue STAT5-null HSCs. Tools that specifically deplete uSTAT5 are currently lacking but would greatly aid dissection of the distinct physiological roles of uSTAT5 and pSTAT5.

It is interesting to consider our results in the light of data that HSCs can be expanded using culture conditions that include high TPO concentrations (100ng/ml).^84^ This observation contrasts with other reports showing that low TPO^85^ and low cytokine environments^86^ better maintain HSC function, and that injection of TPO or a TPO mimetic reduces HSC numbers and HSC function *in vivo*.^87^ Together these data indicate that the effect of TPO is complex and may be concentration and/or context dependent. TPO-driven HSC expansion may require other features of the “Wilkinson” expansion cultures (eg presence of PVA, absence of albumin, hypoxic incubation^84^).

Our results also have therapeutic implications. First, they raise the possibility that ruxolitinib could be a useful strategy to enhance *ex vivo* maintenance of HSCs for gene therapies. Consistent with this concept, human HSPCs cultured in gene therapy conditions display a rapid upregulation of JAK/STAT signalling, and its inhibition improved their long-term repopulation.^88^ Second in patients with an MPN,^88,89^ JAK inhibitors have little if any effect on the level of the mutant clone.^50^ A protective effect of ruxolitinib on mutant HSPCs, may contribute to the limited efficacy of JAK inhibitors. Moreover, an accumulation of mutant HSCs poised to differentiate may also contribute to the JAK-inhibitor discontinuation syndrome, characterised by a rapid life-threatening MPN resurgence after JAK-inhibitor withdrawal.^89^ The cytokine environments of endogenous HSCs in their various niches remain poorly understood and so further studies will be needed to explore the HSC effects of ruxolitinib *in vivo*. However, our results raise the possibility that targeting uSTAT5 or total STAT5 activity may represent attractive therapeutic approaches for myeloid malignancies associated with JAK activation.

## Supplementary Material

Supplemental Materials

## Key Points

Tyrosine-unphosphorylated STAT5 maintains HSCs.

JAK inhibition promotes unphosphorylated STAT5 activity and maintains mouse and human, normal and MPN HSPCs.
