# Maintenance of hematopoietic stem cells by tyrosine-unphosphorylated STAT5 and JAK inhibition^∗^^†^

**DOI:** 10.1182/bloodadvances.2024014046

**Published:** 2024-10-10

**Authors:** Matthew J. Williams, Xiaonan Wang, Hugo P. Bastos, Gabriela Grondys-Kotarba, Qin Wu, Shucheng Jin, Carys Johnson, Nicole Mende, Emily Calderbank, Michelle Wantoch, Hyun Jung Park, Giovanna Mantica, Rebecca Hannah, Nicola K. Wilson, Dean C. Pask, Tina L. Hamilton, Sarah J. Kinston, Ryan Asby, Rachel Sneade, E. Joanna Baxter, Peter Campbell, George S. Vassiliou, Elisa Laurenti, Juan Li, Berthold Göttgens, Anthony R. Green

**Affiliations:** 1 Department of Haematology, Wellcome–Medical Research Council Cambridge Stem Cell Institute, Jeffrey Cheah Biomedical Centre, University of Cambridge, Cambridge, United Kingdom; 2 Department of Haematology, University of Cambridge, Cambridge, United Kingdom; 3 Department of Public Health, School of Public Health, Shanghai Jiaotong University School of Medicine, Shanghai, China; 4 Flow Facility, Cambridge Institute for Medical Research, Cambridge, United Kingdom; 5 Department of Cancer Genomics, Wellcome Sanger Institute, Wellcome Trust Genome Campus, Hinxton, United Kingdom

## Abstract

Adult hematopoietic stem cells (HSCs) are responsible for the lifelong production of blood and immune cells, a process regulated by extracellular cues, including cytokines. Many cytokines signal through the conserved Janus kinase (JAK)/signal transducer and activator of transcription (STAT) pathway in which tyrosine-phosphorylated STATs (pSTATs) function as transcription factors. STAT5 is a pivotal downstream mediator of several cytokines known to regulate hematopoiesis, but its function in the HSC compartment remains poorly understood. In this study, we show that STAT5-deficient HSCs exhibit an unusual phenotype, including reduced multilineage repopulation and self-renewal, combined with reduced exit from quiescence and increased differentiation. This was driven not only by the loss of canonical pSTAT5 signaling, but also by the loss of distinct transcriptional functions mediated by STAT5 that lack canonical tyrosine phosphorylation (uSTAT5). Consistent with this concept, expression of an unphosphorylatable STAT5 mutant constrained wild-type HSC differentiation, promoted their maintenance, and upregulated transcriptional programs associated with quiescence and stemness. The JAK1/2 inhibitor, ruxolitinib, which increased the uSTAT5:pSTAT5 ratio, had similar effects on murine HSC function; it constrained HSC differentiation and proliferation, promoted HSC maintenance, and upregulated transcriptional programs associated with stemness. Ruxolitinib also enhanced serial replating of normal human hematopoietic stem and progenitor cells (HSPCs), calreticulin-mutant murine HSCs, and HSPCs obtained from patients with myelofibrosis. Our results therefore reveal a previously unrecognized interplay between pSTAT5 and uSTAT5 in the control of HSC function and highlight JAK inhibition as a potential strategy for enhancing HSC function during ex vivo culture. Increased levels of uSTAT5 may also contribute to the failure of JAK inhibitors to eradicate myeloproliferative neoplasms.

## Key Points

• Tyrosine-unphosphorylated STAT5 maintains HSCs.
• JAK inhibition promotes unphosphorylated STAT5 activity and maintains normal and myeloproliferative neoplasm HSPCs in mice and humans.

## Visual Abstract

**Figure:**
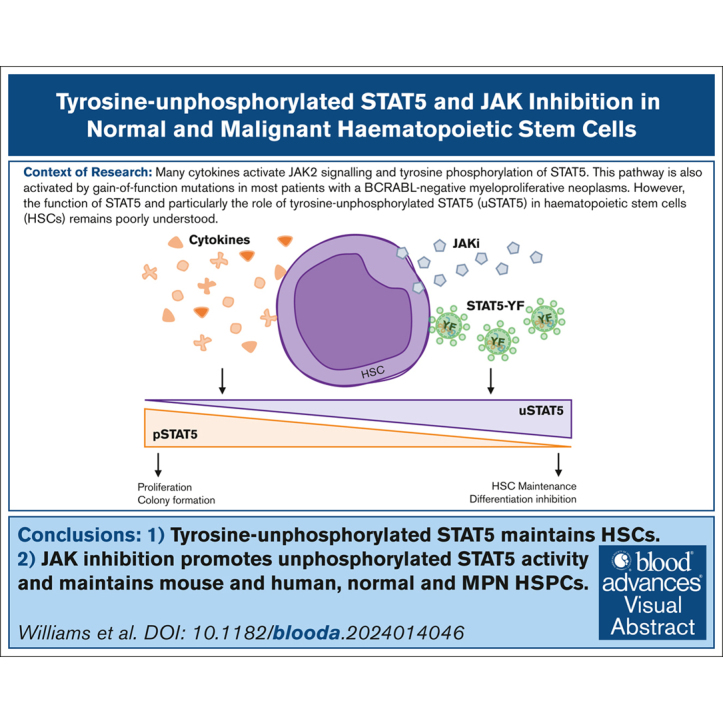

## Introduction

Hematopoietic stem cells (HSCs) are a highly quiescent population of cells responsible for continued production of mature blood cells throughout life.^1^^,^^2^ Their ability to respond to environmental signals is important for the maintenance of homeostasis and for HSCs to respond to a variety of stresses.^3^, ^4^, ^5^, ^6^

The Janus kinase (JAK)–signal transducer and activator of transcription (STAT) pathway regulates multiple developmental and adult stem cell populations^7^, ^8^, ^9^ and is dysregulated in a variety of hematologic malignancies and other cancers.^10^^,^^11^ The STAT5 is an essential downstream mediator of cytokine signaling at multiple stages of hematopoiesis.^12^, ^13^, ^14^, ^15^, ^16^ In eutherian mammals, 2 closely related STAT5 isoforms,^17^ STAT5A and STAT5B, display distinct and redundant functions in different cell types.^18^, ^19^, ^20^, ^21^ Mice that lack both genes, or the N-terminal domains of both genes, develop severe anemia and leukopenia^22^, ^23^, ^24^, ^25^, ^26^ that are associated with reduced survival and proliferation of erythroblasts.^15^^,^^16^ Conversely, high levels of STAT5 activity in hematopoietic stem and progenitor cells (HSPCs) drive erythroid differentiation.^27^^,^^28^

STAT5A and STAT5B contain critical regulatory tyrosine residues (Y694 and Y699) that are essential for the activation of canonical tyrosine-phosphorylated STAT5 (pSTAT5) target genes.^29^^,^^30^ These residues are phosphorylated by (JAKs^31^ that are activated in response to multiple cytokines,^3^, ^4^, ^5^, ^6^ including interleukin-3 (IL-3)^32^ and thrombopoietin.^33^ pSTAT5 accumulates in the nucleus, binds to DNA, and regulates the transcription of target genes.^34^ STAT5 phosphorylation is transient because pSTAT5 rapidly promotes the expression of negative regulators of JAK-STAT signaling, including suppressors of cytokine signaling, tyrosine phosphatases, and protein inhibitors of STATs.^35^^,^^36^

Elevated STAT5 phosphorylation is observed in many hematologic malignancies^37^^,^^38^ and solid tumors.^39^^,^^40^ Activation of the JAK-STAT pathway is especially common in the myeloproliferative neoplasms (MPNs), >90% of which contain driver mutations that activate JAK-STAT signaling.^41^, ^42^, ^43^, ^44^, ^45^, ^46^ JAK inhibitors are used to treat patients with MPN with advanced disease,^47^ but although these can lead to symptomatic improvement, they rarely reduce the allele burden,^48^, ^49^, ^50^ suggesting that they fail to eradicate malignant HSCs.

Loss of both STAT5 genes lead to a reduction in the number of immunophenotypically-defined HSCs^26^^,^^51^^,^^52^ and defective repopulation by fetal liver and adult bone marrow (BM).^26^^,^^53^^,^^54^ STAT5B is dominant in multipotent HPC7 cells^55^ and STAT5B deficient, but not STAT5A deficient, BM showed functional defects in serial transplants.^52^ However, several aspects of STAT5 function in HSPCs remain unclear or have been the subject of conflicting reports. Both an increase^26^^,^^51^^,^^52^ and a reduction in cycling^56^ have been observed in HSPCs after STAT5 loss, whereas STAT5 phosphorylation is associated with increased proliferation.^57^ Moreover, both STAT5 knockdown^55^ and constitutively active STAT5A overexpression^27^^,^^28^ have been reported to increase HSPC differentiation. Insight into at least some of these apparent paradoxes came from the demonstration that STAT5 that lacks phosphorylation of its critical tyrosine (uSTAT5) is present in the nucleus of HSPCs and represses megakaryocytic differentiation by restricting access of the megakaryocytic transcription factors to target genes.^55^ Cytokine-mediated phosphorylation of STAT5 therefore triggers 2 distinct transcriptional consequences, namely activation of a canonical pSTAT5–driven program that regulates proliferation and apoptosis and loss of a uSTAT5 program that restrains megakaryocytic differentiation.

Given our limited understanding of the function of STAT5 in HSCs and the complete lack of information about the role of uSTAT5 in primitive HSCs, we explored these issues using genetically modified mice.

## Methods

### Mice

The wild-type (WT) C57BL/6 (CD45.2), C57BL/6.SJL (CD45.1), and F1 (CD45.1/CD45.2) mice, and calreticulin (CALR):del mutant mice^58^ in this study were used at 10 to 32 weeks of age. STAT5^fl/fl^mice^25^ were kindly gifted by Lothar Hennighausen and were crossed with Mx1Cre mice^59^ to generate STAT5^fl/fl^ with Cre (STAT5^fl/fl^Cre^+^) or without Cre (STAT5^fl/fl^Cre^−^). STAT5 deletion was induced by repeated injections with polyinosinic:polycytidylic acid (Poly:IC). All mice were kept in pathogen-free conditions, and all procedures were performed according to the UK Home Office regulations.

### Smart-seq2 and 10x Genomics single-cell RNA sequencing (scRNAseq) analysis

Single Lin^−^CD150^+^CD45^+^CD48^−^EPCR^+^ (ESLAM) HSCs were sorted from bone marrow mononuclear cells (BMMNCs) using fluorescence-activated cell sorting and processed using Smart-seq2 (accession number: GSE223366). Lineage^−^cKit^+^ (LK) cells were sorted from BMMNCs and processed using 10x Chromium (10x Genomics, Pleasanton, CA; GSE223632). Sorted ESLAM HSCs were transduced with lentivirus containing empty vector (EV), STAT5B-WT, or STAT5B-Y699F (YF). After a 5-day culture, green fluorescent protein–positive (GFP^+^) DAPI^−^ (4′,6-diamidino-2-phenylindole–negative) cells were processed using 10x Chromium (10x Genomics; GSE223680). Sorted ESLAM HSCs were cultured for 5 days with ruxolitinib or dimethyl sulfoxide (DMSO), which were then processed using 10x Chromium (10x Genomics; GSE260462). All data were deposited in the National Center for Biotechnology Information Gene Expression Omnibus.

An institutional review board/research ethics committee approved the protocol for human samples.

## Results

### STAT5 loss leads to defective HSC function

Previous reports showed that STAT5^−/−^ fetal liver and adult BM cells displayed reduced repopulation in transplantation assays,^26^^,^^54^ but it was unclear if this was a consequence of reduced HSC number or whether STAT5^−/−^ HSCs are also functionally impaired. We therefore crossed mice that carried a floxed *Stat5a/5b* allele^25^ with Mx1Cre mice and used Poly:IC to delete both *Stat5a* and *Stat5b* loci with ∼90% efficiency in hematopoietic cells (supplemental Figure 1A-C).

**Figure 1.**
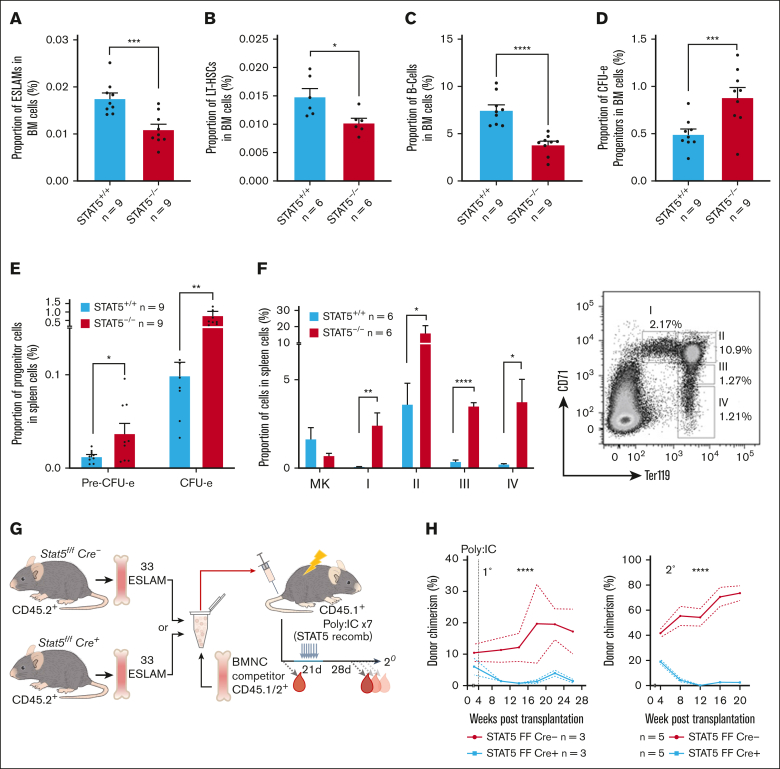

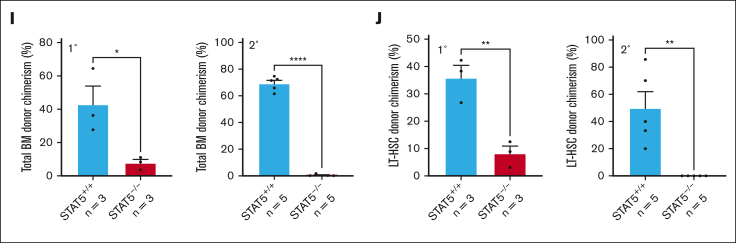
**STAT5 loss leads to defective HSC function.** (A) Bar plot showing the frequency of ESLAM HSCs (CD45^+^CD150^+^CD48^−^EPCR^+^) in BMMNCs from WT and STAT5-deficient mice (mean ± standard error of the mean [SEM]). (B) Bar plot showing the frequency of LT-HSCs (Lin^−^Sca1^−^cKit^+^CD150^+^CD48^−^CD34^−^Flk2^−^) in BMMNCs (mean ± SEM). (C) Bar plot showing the frequency of B cells (B220^+^) in BMMNCs (mean ± SEM). (D) Bar plot showing the frequency of CFU-e progenitors (Lin^−^Sca1^−^cKit^+^CD41^−^CD16/32^−^CD105^+^CD150^−^) in BMMNCs (mean ± SEM). (E) Bar plots showing the frequency of CFU-e and pre–CFU-e (Lin^−^Sca1^−^cKit^+^CD41^−^CD16/32^−^CD105^+^CD150^+^) cells in spleen mononuclear cells (mean ± SEM). (F) Bar plots (left) showing the frequency of megakaryocyte (CD41^+^CD42^+^) and erythroid precursor cells (I, CD71^hi^Ter119^mid^; II, CD71^hi^Ter119^hi^; III, CD71^mid^Ter119^hi^; IV, CD71^low^Ter119^hi^) in spleen mononuclear cells (mean ± SEM) with a representative flow-cytometry plot (right) showing the gating of different stages of erythroid precursor cells in terminal differentiation. (G) Schematic diagram showing that 33 fluorescence-activated cell sorting–purified BM ESLAM HSCs were transplanted into irradiated recipient mice with 5 × 10^5^ competitor BMMNCs. STAT5 was deleted in Cre^+^ donor cells after transplantation by repeated injection (×7) with Poly:IC in recipients. Blood was taken before and after STAT5 deletion and was followed for 5 months after deletion before serial transplantation of 3 × 10^6^ primary recipient BMMNCs. (H) Connected line graphs showing donor chimerism in peripheral blood mononuclear cells at each time point in primary and secondary recipients (mean ± SEM). The dotted line indicates initiation of the Poly:IC injections. The asterisks indicate significant differences by analysis of variance column factor (∗∗∗∗*P* < .0001). (I) Bar plots showing the total BMMNC donor chimerism in primary and secondary recipients (mean ± SEM). (J) Bar plots showing LT-HSC donor chimerism in primary and secondary recipients (mean ± SEM). The asterisks indicate significant differences by Student *t* test (∗∗∗∗*P* < .0001; ∗∗∗*P* < .001; ∗∗*P* < .01; ∗*P* < .05) unless otherwise indicated.

Consistent with previous reports,^25^^,^^26^ a STAT5 deletion led to anemia, leukopenia, and reduced BM cellularity (supplemental Figure 1D-E). In STAT5-deficient BM, the frequencies of immunophenotypic primitive HSCs (both ESLAM; Lin^−^CD150^+^CD45^+^CD48^−^EPCR^+^, and long-term HSC [LT-HSC]; Lin^−^Sca1^+^cKit^+^CD150^+^CD48^−^CD34^−^Flk2^−^; Figure 1A-B) and B cells (Figure 1C) were reduced and the proportion of erythroid progenitors (colony forming unit-erythroid [CFU-e]; Lin^−^Sca1^−^cKit^+^CD41^−^CD16/32^−^CD105^+^CD150^−^) was increased (Figure 1D), but other mature and progenitor cell types were unaltered (supplemental Figure 1F-I). In the spleen, STAT5 deletion reduced the B-cell frequency (supplemental Figure 1J) and increased the frequencies of erythroid progenitors (CFU-e and PreCFU-e; Lin^−^Sca1^−^cKit^+^CD41^−^CD16/32^−^CD105^+^CD150^+^) and all stages of erythroblast differentiation (Figure 1E-F).

Droplet-based (10x Genomics) scRNAseq was performed to assess the HSPC landscape. BM LK (Lin^−^cKit^+^) cells from pairs of STAT5^−/−^ and WT control mice were projected onto a previously published LK data set^60^ and then onto a phenotypically-defined HSPC data set;^61^ cell types were annotated based on their nearest neighbors. Cells within the LT-HSC, short-term HSC (ST-HSC), multipotent progenitor (MPP), myeloid, and early- and miderythroid clusters were relatively reduced in STAT5^−/−^ mice, whereas the abundance of cells within late-erythroid and lymphoid clusters were relatively increased (supplemental Figure 1K). These results confirm and extend previous reports and show that a STAT5 deficiency causes widespread alterations in hematopoietic progenitors, including reduced numbers of HSCs.

In competitive transplantation experiments using highly purified ESLAM HSCs (Figure 1G; supplemental Figure 1L), STAT5-deficient HSCs displayed significantly reduced multilineage repopulation in the blood (Figure 1H; supplemental Figure 1M-O) and BM (Figure 1I; supplemental Figure 1P) of primary recipients. There was almost no repopulation of blood or BM in secondary recipients. Few or no STAT5-deficient LT-HSCs (Lin^−^Sca1^+^cKit^+^CD150^+^CD48^−^CD34^−^Flk2^−^CD45.1^−^CD45.2^+^) were observed in the BM of primary or secondary recipients (Figure 1J; supplemental Figure 1Q). These data demonstrate that STAT5-deficient HSCs are not merely reduced in number but are also functionally impaired and display markedly reduced multilineage repopulation and self-renewal.

### STAT5-deficient HSCs display reduced cell cycle entry, increased differentiation, and reduced generation of lineage-negative progeny

To explore the molecular basis for HSC dysfunction, plate-based scRNAseq was performed on WT and STAT5-deficient ESLAM HSCs (supplemental Figure 2A-C) and led to the identification of 308 differentially expressed genes (adjusted *P* < .01; log fold change greater than ±0.5; supplemental Figure 2D; supplemental Table 1), including canonical STAT5 targets (eg, *Cish*, *Socs2* and *Bcl6*; supplemental Figure 2E).

**Figure 2.**
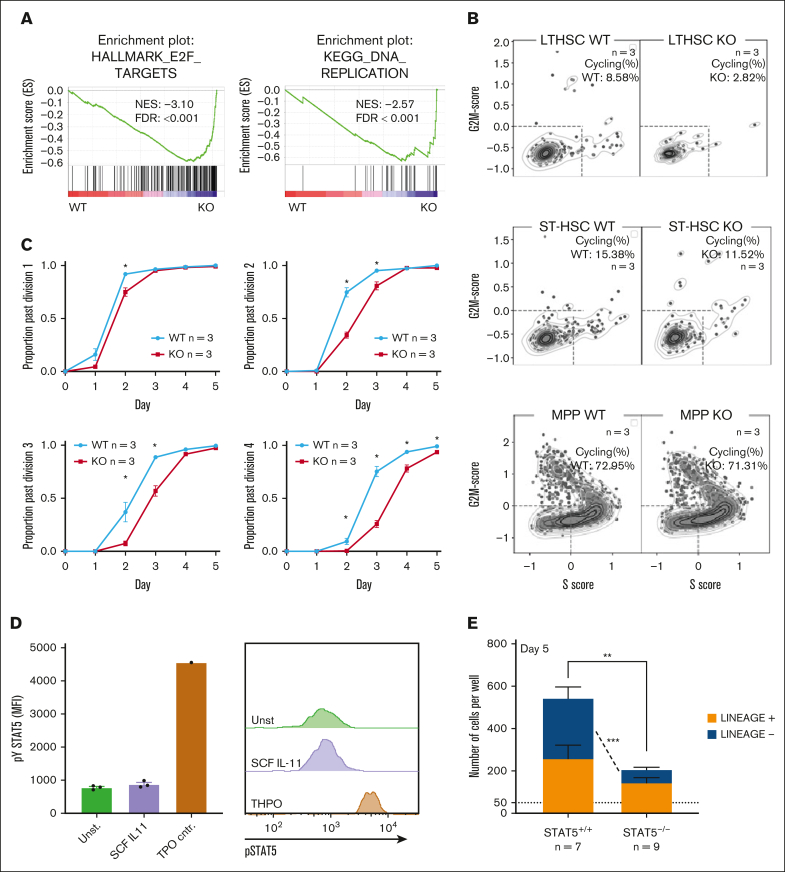

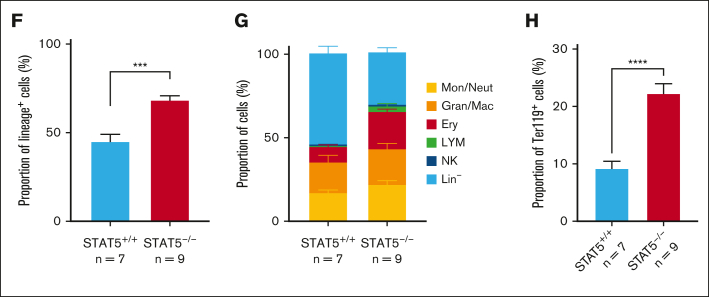
**STAT5-deficient HSCs display reduced cell cycle entry, increased differentiation, and reduced retention of lineage-negative progeny.** (A) Gene set enrichment analysis (GSEA) plots showing depleted cell cycle–related signatures in STAT5-deficient ESLAM (CD45^+^CD150^+^CD48^−^EPCR^+^) HSCs. scRNAseq analysis using the Smart-seq platform was performed on FACS-isolated ESLAM HSCs from STAT5^f/f^ Cre- or STAT5^f/f^ Cre^+^ BM; 132 STAT5-deficient and 132 WT HSCs passed quality control and were used for downstream analysis. The normalized enrichment scores (NES) and false discovery rate (FDR) are indicated. (B) Plots showing the cell cycle scores of transcriptionally defined LT-HSCs, ST-HSCs, and MPPs that were isolated from scRNAseq data sets of WT and STAT5-deficient BM LK cells (STAT5 WT, n = 3; STAT5KO, n = 3). (C) Line graphs showing the proportion of ESLAM HSCs that past first, second, third, and fourth divisions at given timepoints (y-axis) in the single cell in vitro analysis (mean ± SEM). The results are from 3 biological replicates across 3 experiments. (D) Bar plots (left) showing the mean fluorescent intensity (MFI) of pSTAT5 antibody staining of ESLAM HSCs by intracellular flow-cytometry analysis in unstimulated maintenance culture conditions^62^ (SCF/IL-11) or TPO (200 ng/mL) positive control conditions (mean ± SEM). Right; representative histogram of the intracellular flow-cytometry analysis showing the intensity of pSTAT5 staining in each condition. The results are from 3 biological replicates. (E) Bar plot showing the number of cells in each well at day 5 from an initial culture of 50 ESLAM HSCs in SCF/IL11 maintenance conditions. The number of cells that expressed mature lineage markers (Ter119^+^, Ly6g^+^, CD11b^+^, NK1.1^+^, B220^+^, CD19^+^, or CD3e^+^) and the number of lineage-negative cells are shaded in different colours (mean ± SEM). The results are from 9 to 7 biological replicates across 4 experiments. (F) Bar plot showing the proportion of cells that expressed mature lineage markers after 5 days in culture that originated from 50 ESLAM HSCs (mean ± SEM). (G) Bar plot showing the proportion of cells that expressed specific mature lineage markers for monocytes (Mons) and granulocytes (Grans; Ly6g^+^), Grans and macrophages (MacsCD11b^+^), erythroid (Ery; Ter119^+^), lymphocytes (LYMs; CD3e^+^/CD19^+^/B220^+^), and natural killer cells (NK; NK1.1^+^) after 5 days in culture that originated from 50 ESLAM HSCs (mean ± SEM). (H) Bar plot showing the frequency of Ter119^+^ cells after 5 days in culture that originated from 50 ESLAM HSCs (mean ± SEM). Asterisks indicate significant differences as determined by Student *t* test (∗∗∗∗*P* < .0001; ∗∗∗*P* < .001; ∗∗*P* < .01; ∗*P* < .05). KO, knockout.

Gene set enrichment analysis identified 12 signatures that were enriched in STAT5-deficient ESLAM HSCs (false discovery rate <0.25; supplemental Table 2), including the Wnt, Hedgehog and Kras pathways, and 35 signatures that were depleted (false discovery rate <0.25; supplemental Table 2), including JAK-STAT signaling, DNA repair, and the unfolded protein response. The most significantly depleted gene sets were cell cycle–related signatures, including E2F targets and DNA replication (Figure 2A; supplemental Figure 2F). Consistent with this observation, analysis of our separate 10X LK cell data sets showed that, when compared with WT controls, far fewer STAT5-deficient LT-HSCs were in cycle (8.58% vs 2.82%; Figure 2B). A less pronounced reduction in cell cycling was seen in STAT5-deficient ST-HSCs and MPPs.

Ki-67/DAPI staining showed that, when compared with WT mice, STAT5-deficient mice had increased proportions of ESLAM HSCs in G_0_ and reduced proportions in G_1_, although this did not reach statistical significance (supplemental Figure 2G-I). However, it is challenging to detect increases in dormancy in populations that are already highly quiescent, and the Ki-67/DAPI analysis represents a snapshot, which may not capture subtle but relevant changes in quiescence maintenance. We therefore measured the division kinetics of single HSCs (as previously described^62^). STAT5-deficient ESLAM HSCs were indeed slower to enter their first and subsequent divisions (Figure 2C), indicating transient cell cycle arrest or compounded delays in cell cycle entry, thus demonstrating that STAT5 is required for normal HSC cell cycle progression.

The functional consequences of a STAT5 deficiency described above reflect the combined effect of losing both pSTAT5 and uSTAT5. To identify the consequences attributable to a loss of uSTAT5, experimental conditions that precluded STAT5 phosphorylation were required. We suspected that stem-cell factor (SCF) and IL-11 media (previously described to maintain HSCs^62^) would not activate STAT5 phosphorylation in HSCs. Indeed, the pSTAT5 levels in ESLAM HSCs cultured in SCF/IL-11 were not significantly higher than those in cytokine-starved conditions (Figure 2D). After 5 days in this culture condition, STAT5-deficient ESLAM HSCs produced fewer cells overall with markedly fewer lineage-negative cells (Figure 2E) and an increase in the proportion of lineage-positive cells (Figure 2F). The proportion of each lineage increased (Figure 2G; supplemental Figure 2J-M) with the erythroid lineage (Ter119^+^) reaching statistical significance (Figure 2H). Similar results were obtained with ESLAM HSCs cultured for 4 or 6 days (supplemental Figure 2N) with no difference in the frequency of apoptotic cells (supplemental Figure 2O). These data indicate that loss of uSTAT5 is responsible for increased HSC differentiation and reduced generation of lineage-negative cells.

Together, these results therefore demonstrate that STAT5 loss leads to an unusual HSC phenotype with reduced cell cycle progression but yet increased differentiation.

### Unphosphorylated STAT5 constrains HSC differentiation and upregulates transcriptional programs associated with HSC maintenance

**Figure 3.**
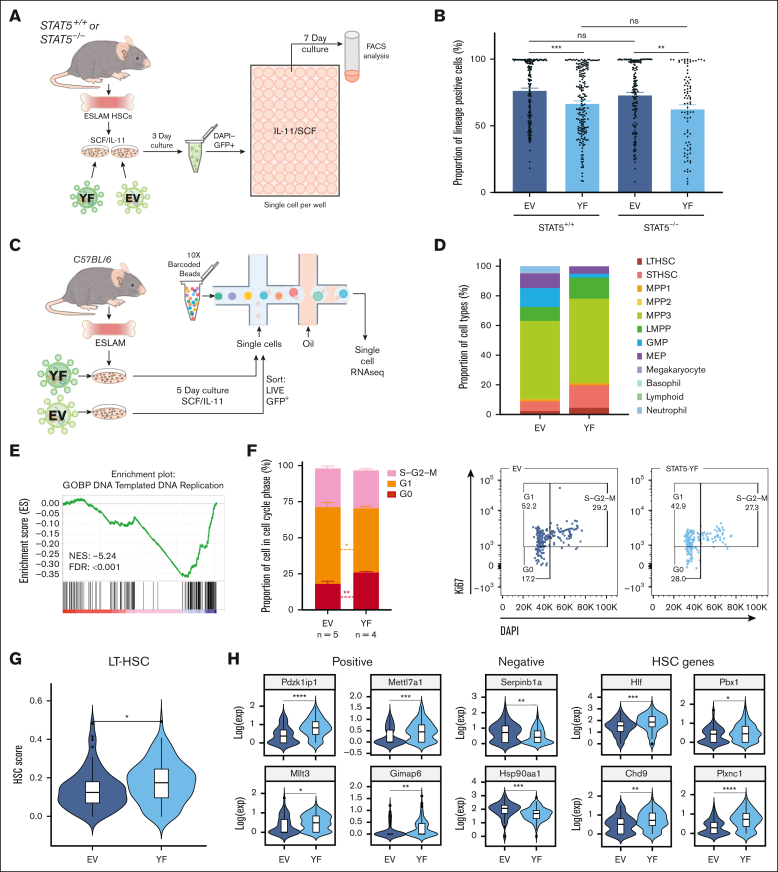
**Unphosphorylated STAT5 constrains HSC differentiation and upregulates transcriptional programs associated with HSC maintenance.** (A) Schematic diagram showing the experimental outline of the ex vivo functional analysis of STAT5-deficient or WT ESLAM (CD45^+^CD150^+^CD48^−^EPCR^+^) HSCs that were transduced with lentivirus containing STAT5B-YF or EV in maintenance cultures.^62^ After 3 days of transduction, GFP^+^ living cells were FACS sorted into single-cell assays. (B) Bar plots showing the proportion of cells expressing mature lineage markers (Ter119^+^/Ly6g^+^/CD11b^+^/B220^+^/CD3e^+^). Each dot represents a single clone and bars represent the mean lineage-positive marker frequency (±SEM). Asterisks indicate significant differences as determined by Student *t* tests (∗∗∗*P* < .001; ∗∗*P* < .01). The results are from 6 to 5 independent biological replicates across 5 experiments in STAT5^+/+^ settings and 4 to 3 independent biological replicates across 3 experiments in STAT5^−/−^ settings. (C) Schematic diagram showing the outline of the scRNAseq analysis of WT ESLAM HSCs that were transduced with lentivirus containing STAT5B-YF or EV in maintenance cultures^62^ and that were allowed to expand for 5 days. GFP^+^ living cells were then sorted for 10X Genomics scRNAseq. (D) Bar plots showing the proportion of annotated cell types in GFP^+^ HSC-derived cultures after 5 days in SCF/IL-11 cultures; single cells were projected onto a previously published scRNAseq data set of LK HSPC cells^60^ and then onto a phenotypically-defined HSPC data set,^61^ and cell types were annotated based on their nearest neighbors to ascribe cell identity and cell type annotation. The results are from 2 independent biological replicates in 2 experiments. (E) Gene set enrichment plot showing that STAT5-YF–infected, transcriptionally defined LT-HSCs (n = 83) are depleted in a DNA replication gene signature when compared with EV-infected LT-HSCs (n = 53). The NES (−5.24) and FDR (<0.001) are indicated. (F) Left: bar plots showing the cell cycle phase frequency of ESLAM HSC-derived cultures infected with either EV (n = 5) or STAT5-YF (n = 4) lentivirus after 5 days in maintenance culture media.^62^ The cell cycle status was derived from Ki67/DAPI staining (right). G_0_ represents quiescent cells that are Ki67^low^DAPI^low^; G_1_ represents cells in the early growth phase, which are Ki67^high^DAPI^low^; S-G2-M represents cells in DNA synthesis, late growth, and mitosis stages of active cell cycling and are Ki67^high^DAPI^high^. (G) Violin plot showing the geometric mean distribution of HSC scores in LT-HSCs expressing STAT5B-YF or EV. The HSC score was calculated using the HSC score tool that identifies potential mouse BM HSCs from scRNAseq data.^63^ This tool considers the expression of genes that are either positively or negatively corelated with HSC long-term repopulating capacity.^64^ (H) Violin plots showing significantly differentially expressed genes that are positively associated with functional long-term repopulating HSCs (*Pdzk1ip1*, *Mettl7a1*, *Mllt3*, and *Gimap1*), negatively associated with functional long-term repopulating HSCs (*Serpinb1a* and *Hsp90aa1*), or genes with reported functions in maintaining HSCs (*Hlf*, *Chd9*, *Pbx1*, and *Plxnc1*). All data were combined from 2 independent experiments. Asterisks indicate significant differences as determined by Student *t* tests (∗∗∗∗*P* < .0001; ∗∗∗*P* < .001; ∗∗*P* < .01; ∗*P* < .05). GMP, granulocyte-macrophage progenitor; LMPP, lymphoid-myeloid progenitor; MEP, Meg/Ery progenitors; ns, not significant.

To further explore the role of uSTAT5 in HSCs, we used a lentiviral expression approach. STAT5B is the dominant form of STAT5 protein in multipotent HPC7 cells^55^ and long-term repopulating HSCs.^52^ STAT5-YF, which prevents phosphorylation at the critical tyrosine residue, was introduced into STAT5^+/+^ or STAT5^−/−^ ESLAM HSCs, along with EV controls (Figure 3A). STAT5-YF and EV constructs showed comparable expansion and survival in STAT5^+/+^ and STAT5^−/−^ HSC-derived clones (supplemental Figure 3A-B), but STAT5-YF expression led to reduced differentiation in the STAT5^+/+^ and STAT5^−/−^ clones (Figure 3B). These observations are in accord with our studies of STAT5^−/−^ HSCs, which indicated that loss of uSTAT5 enhances their differentiation (see above). Thus, both the knockout and overexpression approaches indicate that uSTAT5 constrains HSC differentiation. STAT5-YF expression increased the total STAT5 levels two- to threefold in Lin^−^Sca1^−^cKit^+^ (LSKs; supplemental Figure 3C-D), and so our results indicate that the functional consequence of STAT5-YF is a two- to threefold increase in uSTAT5, because the vast majority of potentially phosphorylatable STAT5 (ie, can be phosphorylated by thrombopoietin) remains unphosphorylated in SCF/IL-11^62^ media (supplemental Figure 3E).

The transcriptional consequences of STAT5-YF expression in ESLAM HSCs were explored using 10x Genomics scRNAseq (Figure 3C). Because STAT5^−/−^ and STAT5^+/+^ HSCs responded similarly to STAT5-YF overexpression and because STAT5-deficient HSCs are less abundant, STAT5^+/+^ HSCs were used for this analysis. *Stat5b* transcripts increased twofold in STAT5-YF–infected cells (supplemental Figure 3F), consistent with the protein levels (supplemental Figure 3B). Infected cells were projected onto a previously published scRNAseq data set of LK cells^60^ and then a phenotypically-defined HSPC data set,^61^ and cell types were annotated based on their nearest neighbors. When compared with control EV cultures, STAT5-YF cultures contained fewer differentiated cell types (eg, granulocyte-macrophage progenitors, Meg/Ery progenitors, and neutrophils) but more early stem/progenitor cells (LT-HSCs and ST-HSCs; Figure 3D; supplemental Figure 3G). These results align well with our functional evidence that STAT5-YF constrains differentiation.

Within transcriptionally defined LT-HSCs, STAT5-YF expression was associated with the upregulation of 321 genes and downregulation of 120 genes (supplemental Table 3), representing both direct and indirect consequences of STAT5-YF expression. The expression levels of pSTAT5 target genes (*Pim1, Ccnd1, Mcl1*, and *Sod2*) were unaffected by STAT5-YF expression (supplemental Figure 3H), and gene set enrichment analysis failed to identify enrichments or depletions in canonical STAT5 target gene sets (data not shown), indicating that STAT5-YF is not exerting a dominant negative effect. Consistent with this concept, the vast majority of phosphorylatable STAT5 remains unphosphorylated in HSCs cultured in SCF/IL11 (Figure 2D; supplemental Figure 3E), however, our results cannot completely exclude the existence of very low levels of pSTAT5 below our detection levels.

In HPC7 cells, we have previously shown that uSTAT5 repressed several megakaryocytic genes (*Mpl, Vwf, Gp9,* and *F2r*) and that it competed with ERG in regulating *Mpl* and *F2r*. We therefore explored whether similar effects could be found in highly purified HSCs. The expression levels of *Mpl, Vwf, Gp9*, and *F2r* were not increased in STAT5^−/−^ HSCs or reduced in STAT5-YF–expressing HSCs (supplementary Figure 3I), which likely reflect different transcriptional programs within HPC-7 cells (similar to Meg/Ery progenitors and derived from embryonic stem cells) and HSCs.

Cell cycle gene signatures were significantly depleted in STAT5-YF–infected LT-HSCs (Figure 3E; supplemental Figure 3J). More STAT5-YF LT-HSCs were in the G_0_/G_1_ phases (supplemental Figure 3K), and Ki67/DAPI analysis in STAT5^+/+^ ESLAM-derived cultures confirmed that STAT5-YF expression increased the frequency of HSCs in G_0_ (Figure 3F), collectively indicating that STAT5-YF expression is associated with increased HSC quiescence. STAT5-YF–expressing LT-HSCs exhibited higher HSC scores than EV expressing HSCs (Figure 3G) when a previously described algorithm was used, which identifies durable, long-term repopulating HSCs^63^ and takes into account the expression of genes that correlate either positively or negatively with HSC function.^64^ STAT5-YF HSCs also exhibited higher HSC scores using 2 other published HSC signatures (supplemental Figure 3L).^65^^,^^66^ Indeed, positively-associated HSC score genes were upregulated in STAT5-YF LT-HSCs, whereas anticorrelated genes were downregulated, and other genes reported to promote HSC maintenance were also upregulated (Figure 3H).

Together, our data therefore demonstrate that STAT5-YF restrains HSC differentiation, increases HSC quiescence, and regulates transcriptional networks associated with increased HSC maintenance.

### Unphosphorylated STAT5 enhances HSPC clonogenicity in vitro and HSC maintenance in vivo

**Figure 4.**
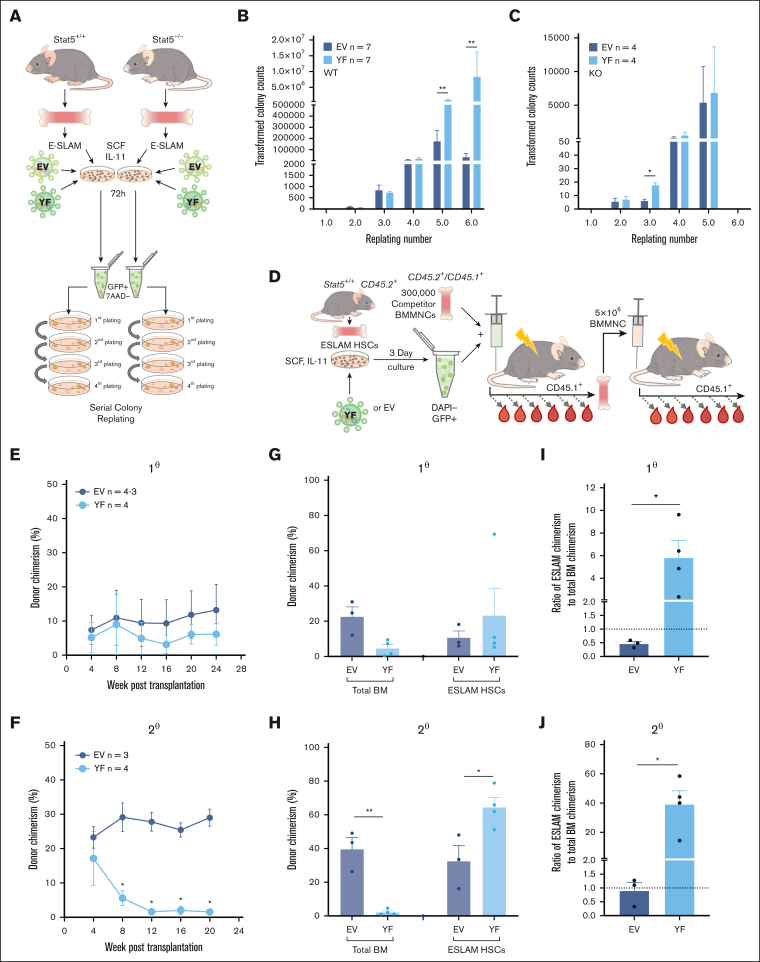
**Unphosphorylated STAT5B enhances HSPC clonogenicity in vitro and HSC maintenance in vivo.** (A) Schematic diagram showing the experimental outline of the serial colony replating assays of STAT5-deficient or WT ESLAM (CD45^+^CD150^+^CD48^−^EPCR^+^) HSCs that were transduced with lentivirus containing STAT5B-YF or EV in SCF/IL-11 maintenance cultures. After 3 days of transduction, GFP^+^ living cells were sorted for serial colony replating assays. (B) Bar plots showing the transformed colony numbers derived from WT HSPCs transduced with YF or EV lentivirus. Transformed colony counts = ((colony number × dilution factor) ÷ starting number of HSCs) (mean ± SEM). The results were from 4 independent experiments and 7 biological replicates. Asterisks indicate the significant differences as determined by Mann-Whitney *U* tests (∗∗*P* < .01). (C) Bar plots showing the transformed colony numbers of STAT5-deficient HSPCs transduced with YF or EV lentivirus. Transformed colony counts = ((colony number × dilution factor) ÷ starting number of HSCs) (mean ± SEM). The results were from 3 independent experiments and 4 biological replicates. Asterisks indicate significant differences as determined by Mann-Whitney *U* tests (∗*P* < .05). (D) Schematic diagram showing the outline of the in vivo functional analysis of WT ESLAM HSCs that were transduced with lentivirus containing STAT5B-YF or EV. FACS-sorted WT ESLAM HSCs (CD45.2^+^) were infected with lentivirus and cultured for 3 days in maintenance cultures and then an equal number of GFP^+^ cells were FACS sorted (112 GFP^+^ cells per recipient) and injected into irradiated recipients (CD45.1^+^) with 3 × 10^5^ competitor BMMNCs (CD45.1^+^/CD45.2^+^). Donor chimerism was monitored every 28 days for more than 6 months. Secondary transplantation was then performed using 5 × 10^6^ BMMNCs from primary recipients. BMMNCs from 1 primary recipient were transplanted into up to 2 recipients. (E) Connected line graph showing donor chimerism in primary recipients (mean ± SEM) (experiment described in panel D). Chimerism was derived as the ratio of donor: (donor + competitor). (F) Connected line graph showing donor chimerism in secondary recipients (mean ± SEM) (experiment described in panel D). Chimerism was derived as the ratio of donor/(donor + competitor). (G) Bar plots showing donor chimerism in total BMMNCs and ESLAM HSCs in the BM of the primary transplant recipients (mean ± SEM). (H) Bar plots showing donor chimerism in total BMMNCs and ESLAM HSCs in the BM of the secondary transplant recipients (mean ± SEM). (I) Bar plots showing the ratio of ESLAM HSC donor chimerism to total BMMNC chimerism in primary recipient BM (mean ± SEM; dotted line indicating 1:1 ratio). (J) Bar plots showing the ratio of ESLAM HSC donor chimerism to total BMMNC chimerism in secondary recipient BM (mean ± SEM; dotted line indicating 1:1 ratio). Chimerism was derived as the ratio of donor/(donor + competitor). Asterisks indicate significant differences as determined by Student *t* tests (∗∗*P* < .01; ∗*P* < .05).

We subsequently explored the effect of STAT5-YF expression on HSC function (Figure 4A). In serial colony replating assays, STAT5^+/+^ HSCs expressing STAT5-YF displayed enhanced colony generation in 4 independent experiments (Figure 4B; supplemental Figure 4A), demonstrating that uSTAT5 is sufficient to enhance the generation of clonogenic progeny by WT HSCs. The introduction of STAT5-YF had no effect on the replating of STAT5^−/−^ HSCs, but these cells produced far fewer colonies for a shorter duration than WT cells (Figure 4C; supplemental Figure 4B), indicating a requirement for pSTAT5 in the replating assay, likely through its role in driving proliferation.^55^ Indeed, STAT5 phosphorylation was readily detectable in HSCs cultured in the replating assay media, which contained IL-3 and IL-6 (supplemental Figure 4C).

In competitive transplantation experiments (Figure 4D), when compared with control EV-infected HSCs, those that carried STAT5-YF generated peripheral blood donor chimerism that was modestly reduced in primary recipients (Figure 4E) and dramatically reduced in secondary recipients (Figure 4F; supplemental Figure 4D). Furthermore, when compared with control EV-infected HSCs, HSCs infected with STAT5-YF gave rise to a reduction in the total BM chimerism but increased ESLAM-HSC chimerism in primary recipients (Figure 4G), an observation that was even more striking in secondary recipients (Figure 4H). Within individual primary recipients, the ratio of HSC chimerism to total BM chimerism was substantially higher for mice that received STAT5-YF HSCs than for those that had received EV HSCs (Figure 4I). This pattern was even more striking in secondary transplant recipients (Figure 4J).

Together, our results therefore indicate that STAT5-YF expression enhances the clonogenicity of HSCs ex vivo and increases HSC chimerism while restricting their repopulating capacity in vivo.

### Ruxolitinib enhances HSPC clonogenicity and maintains transplantable HSCs

**Figure 5.**
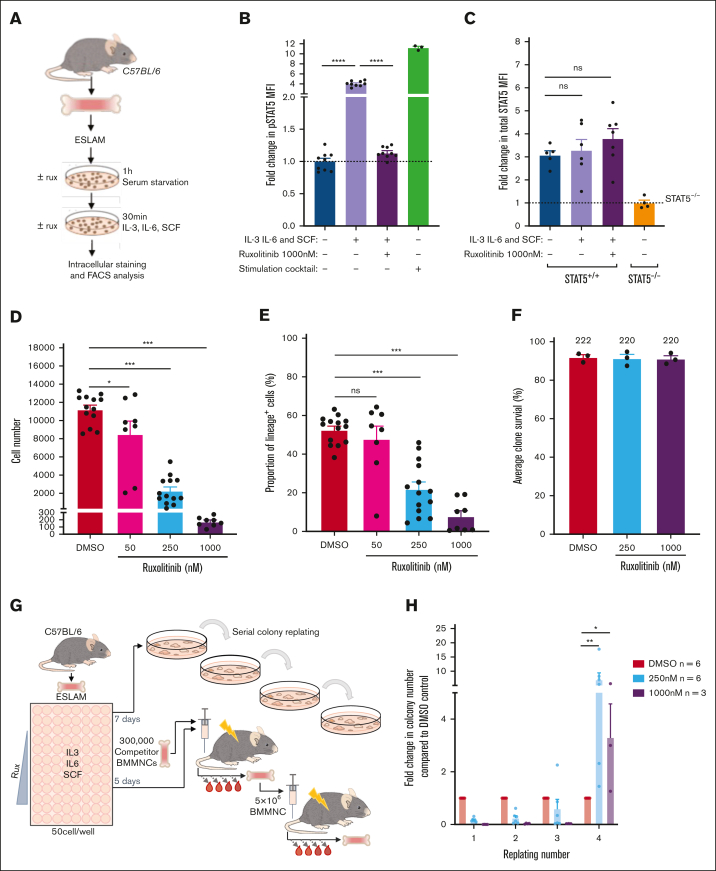

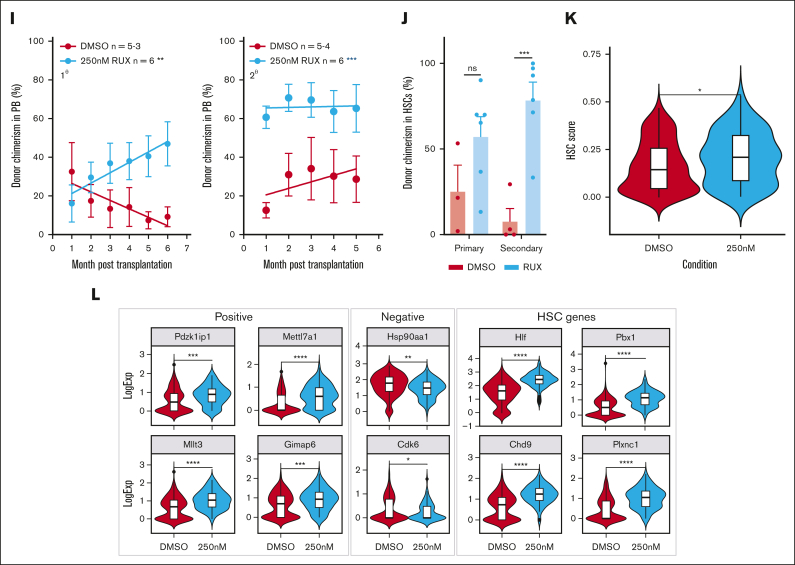
**Ruxolitinib (RUX) enhances HSPC clonogenicity and maintains transplantable HSCs.** (A) Schematic diagram showing intracellular flow cytometric analysis of STAT5 proteins in RUX-treated WT ESLAM HSCs (CD45^+^CD150^+^CD48^−^EPCR^+^). WT ESLAM HSCs were sorted into serum–starved media and starved for 1 hour before a 30-minute stimulation with complete medium containing IL-3, IL-6, and SCF in the presence or absence of RUX,^67^ and a stimulation cocktail containing thrombopoietin (THPO), Flt3-L, and interferon alfa for positive control was included. Cells were then fixed and stained for intracellular flow cytometry. (B) Bar plots showing the MFI of pSTAT5 antibody staining in ESLAM HSCs, described in panel A, normalized to the unstimulated condition, which is indicated with the dotted line (mean ± SEM). Each dot represents the MFI of ESLAMs from a single mouse. The results are from 3 independent experiments. (C) Bar plots showing the MFI of total-STAT5 (tSTAT5) antibody staining in ESLAM HSCs, described in panel A, normalized to STAT5-deficient HSPCs, which is indicated with the dotted line (mean ± SEM). The results are from 3 independent experiments. (D) Bar plots showing cell number per well in HSC-derived cultures at each dose of RUX or vehicle after 7 days (mean ± SEM). A total of 50 ESLAMs were seeded per well in 96-well plates in IL-3/IL-6/SCF^67^ cultures and were treated with DMSO or the indicated doses of RUX. The results are from 6 independent experiments. (E) Bar plot showing the proportion of cells that expressed lineage-positive markers (Ter119^+^/Ly6g^+^/CD11b^+^/B220^+^/CD3e^+^) after 7 days in culture at different concentrations (nM) of RUX (mean ± SEM). A total of 50 ESLAMs were seeded per well in 96-well plates in IL-3/IL-6/SCF^67^ cultures and were treated with the indicated doses of RX. The results are from 6 independent experiments. (F) Bar plots showing the clone survival rate of single HSCs after 5 days in culture. Single ESLAM HSCs were sorted per well and treated with vehicle or RUX. Clone survival rate was the proportion of wells that contained cells at day 5. Each dot represents the frequency of surviving clones from each of 3 independent experiments; the bars show the mean ± SEM. (G) Schematic diagram showing the serial colony replating assays and in vivo functional analysis for ESLAM HSCs treated with RUX or vehicle. A total of 50 WT ESLAM HSCs were sorted per well into complete media^67^ with scaled doses of RUX or vehicle. Cells were harvested after 7 days and plated into serial colony replating assays. ESLAM HSC (CD45.2^+^)–derived cells after 5 days in culture were harvested and transplanted into lethally irradiated recipient mice (CD45.1^+^) with 3 × 10^5^ fresh BMMNCs from competitor mice (CD45.1^+^/CD45.2^+^). Blood was analyzed every 28 days for 6 months. Secondary transplants were then set up by transplanting 3 × 10^6^ BM cells from the primary transplant recipients. (H) Bar plots showing the number of colonies produced by HSC-derived cultures treated with vehicle or RUX (250 or 1000 nM) for 7 days, normalized to the number of colonies produced by vehicle-treated cultures at each week of replating. The results are shown as mean ± SEM and were from 5 independent experiments, 3 of which included 1000 nM. Asterisks indicate significant differences as determined by Mann-Whitney *U* tests (∗∗*P* < .01; ∗*P* < .05). (I) Scatter dot plot with linear regression line of best fit showing the peripheral blood donor chimerism in primary (left) and secondary (right) recipients transplanted with 5-day ex vivo cultured HSCs with RUX or vehicle. A total of 50 ESLAMs from WT mice were seeded per well in IL-3/IL-6/SCF culture conditions and given DMSO or 250 nM of RUX for 5 days before the cells were harvested and pooled for each condition, and an equivalent of 10 starting ESLAMs was transplanted per recipient with 3 × 10^5^ competitor BM cells. Each dot indicates the mean donor chimerism and are shown as mean ± SEM. Black asterisks indicate significant differences in the slopes of the linear regression modeling that compared chimerism of RUX-treated donor cell with DMSO-treated donor cell chimerism in the primary recipients (∗∗*P* < .01). Blue asterisks indicate significant differences in the y-intercepts of linear regressions modeling that compared chimerisms of RUX-treated donor cells with DMSO-treated donor cells in secondary transplants (∗∗∗*P* < .001). (J) Bar plots showing the donor chimerism within the ESLAM HSC compartment at the end of primary and secondary recipients of 5-day ex vivo cultured HSCs with RUX or vehicle. The data are shown as the mean ± SEM. (K) Violin plot showing the geometric mean distribution of HSC scores in LT-HSCs from the 10x scRNAseq data set of the cells treated with RUX or DMSO. The scores were calculated using the HSC score tool that identifies potential mouse BM HSCs from scRNAseq data.^63^ This tool considers the expression of genes that are either positively or negatively correlated with HSC long-term repopulating capacity.^64^ (L) Violin plots showing significantly differentially expressed genes that are positively associated with functional long-term repopulating HSCs (*Pdzk1ip1*, *Mettl7a1*, *Mllt3*, and *Gimap6*), negatively associated with functional long-term repopulating HSCs (*Hsp90aa1* and *Cdk6*), or genes with reported functions in maintaining HSCs (*Hlf*, *Pbx1*, *Chd9*, and *Plxnc1*). All data were combined from 2 independent experiments. Asterisks indicate significant differences as determined by Student *t* tests (∗∗∗∗*P* < .0001; ∗∗∗*P* < .001; ∗∗*P* < .01; ∗*P* < .05) unless otherwise indicated. ns, not significant.

JAK inhibitors, such as ruxolitinib, are predicted to increase the ratio of uSTAT5 to pSTAT5. Indeed, ruxolitinib treatment of cells with activated JAK/STAT signaling (driven by mutant JAK2 or mutant CALR), led to a dramatic reduction in pSTAT5 without a fall in total STAT5 protein levels (supplemental Figure 5A). In ESLAM HSCs, the levels of pSTAT5 (but not pSTAT1 or pSTAT3) were induced fourfold in a ruxolitinib-sensitive manner when exposed to SCF, IL3, and IL6 (Figure 5A-B; supplemental Figure 5B-C). The total STAT5 protein levels were unchanged (Figure 5C), and pSTAT5 target genes such as *Cish* and *Pim1* were downregulated in HSCs exposed to ruxolitinib (supplemental Figure 5D).

Ruxolitinib reduced, in a dose-dependent manner, the progeny generated by ESLAM HSCs (Figure 5D; supplemental Figure 5E-F) and the proportion of lineage-positive cells (Figure 5E; supplemental Figure 5G-H). Two other JAK inhibitors, fedratinib and momelotinib, similarly reduced the expansion and differentiation of ESLAM HSCs in culture (supplemental Figure 5I-J). Treatment with ruxolitinib was not accompanied by a reduction in HSPC viability; even single ESLAM HSCs cultured with high doses of ruxolitinib (eg, 1000 nM, well above the therapeutic range) showed no difference in the proportion of wells containing 1 or more viable cells at 5 days (Figure 5F). Moreover, treatment of lineage-depleted BM cells with ruxolitinib overnight led to apoptosis of mature cell types, but it had little effect on LK cells, suggesting that ruxolitinib does not affect survival of early HSPCs (supplemental Figure 5K).

To investigate the effect of ruxolitinib on HSC function, serial colony replating assays and competitive transplants were performed (Figure 5G). When compared with vehicle-treated HSCs, those exposed to ruxolitinib formed significantly more colonies in the final week of replating assays (Figure 5H; supplemental Figure 5L-M), indicating that ruxolitinib increased the maintenance of clonogenic HSPCs in precultures. In 2 independent competitive repopulation experiments, vehicle-treated control cells gave rise to a donor peripheral blood chimerism that gradually fell over the 5-month study period (Figure 5I; supplemental Figure 5N) as previously reported for cultured HSC donors.^68^ In marked contrast, ruxolitinib-treated HSCs gave rise to a donor peripheral blood chimerism at levels that were initially lower than that of controls and then were maintained or increased. In secondary recipients, donor HSCs originally treated with ruxolitinib displayed significantly higher peripheral blood chimerism levels (Figure 5I; supplemental Figure 5O). Moreover, primary recipient mice that received HSCs precultured with ruxolitinib displayed increased ESLAM HSC chimerism levels, an effect that was even more marked in secondary recipients (Figure 5J).

scRNAseq was used to explore the transcriptional consequences of ruxolitinib (supplemental Figure 5P). Ruxolitinib-treated ESLAM HSC-derived cultures exhibited a reduction in the expression of canonical pSTAT5 target genes (supplemental Figure 5Q) and contained more transcriptionally defined LT-HSCs and ST-HSCs (supplemental Figure 5R). When compared with control LT-HSCs, ruxolitinib-treated LT-HSCs were depleted in cell cycle gene signatures (supplemental Figure 5S) and possessed a greater frequency of cells in G_0_/G_1_ (supplemental Figure 5T). These data were confirmed by Ki67/DAPI analysis at 18 hours and 5 days of treatment (supplemental Figure 5U-V), collectively showing that ruxolitinib promotes HSC quiescence ex vivo.

Consistent with their increased quiescence, ruxolitinib-treated LT-HSCs showed increased HSC fitness scores (Figure 5K; supplemental Figure 5W) when using the 3 different published scoring methods^63^^,^^65^^,^^66^ that also demonstrated increased HSC scores for STAT5-YF–treated LT-HSCs (Figure 3G; supplemental Figure 3J). Ruxolitinib-treated LT-HSCs also showed increased scores for a signature derived by comparing STAT5-YF–expressing LT-HSCs with EV-transduced controls (supplemental Figure 5X). Furthermore, ruxolitinib increased the expression of positively-associated HSC score genes, reduced the expression of negatively-associated HSC score genes, and increased the expression of multiple other genes associated with HSC maintenance (Figure 5L) in a manner similar to STAT5-YF expression (Figure 3H). Several of these genes (eg, *Pdzk1ip1, Gimap6, Hlf, Plxnc1*, and *Chd9*) had previously been identified by chromatin-immunoprecipitation studies^55^ as direct targets of uSTAT5 (supplemental Figure 5Y).

Together, our data demonstrate that ruxolitinib pretreatment reduced HSC differentiation, increased HSC quiescence, and enhanced the maintenance of transplantable HSCs during ex vivo culture. Moreover, the transcriptional consequences of ruxolitinib closely paralleled those observed for STAT5-YF–expressing HSCs (Figure 3G-H), indicating that the effects of ruxolitinib are mediated, at least in part, by uSTAT5.

### Ruxolitinib maintains murine and human MPN HSPCs

Ruxolitinib alleviates symptoms, reduces splenomegaly, and modestly extends the overall survival in a subset of patients with MPN with advanced disease.^47^, ^48^, ^49^ However, it has little or no effect on the allele burden and disease progression,^48^^,^^49^ suggesting that ruxolitinib does not eradicate malignant HSCs. This has been attributed to ruxolitinib having a narrow therapeutic window as a consequence of its dose-limiting toxicity.^69^^,^^70^ However, our data raise the possibility that JAK inhibitors might also inherently promote the maintenance of mutant HSCs by increasing the levels of uSTAT5.

**Figure 6.**
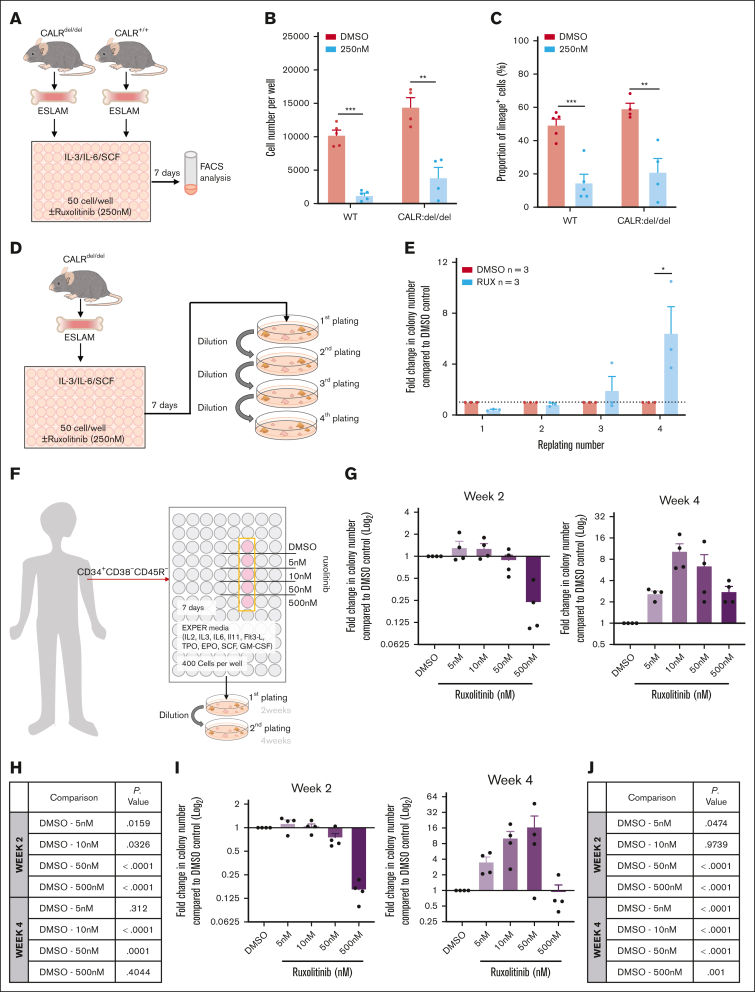
**RUX maintains murine and human MPN HSPCs.** (A) Schematic diagram showing the in vitro functional assays of murine ESLAM HSCs (CD45^+^CD150^+^CD48^−^EPCR^+^) treated with RUX or DMSO. ESLAM HSCs were FACS isolated from CALRdel/del (n = 4) mutant mice and were then cultured for 7 days in IL-3/IL-6/SCF media^67^ with DMSO or 250 nM of RUX before analysis by flow cytometry. (B) Bar plots showing cell number per well in HSC-derived cultures treated with vehicle or 250 nM RUX after 7 days (mean ± SEM). (C) Bar plots showing the proportion of cells that expressed lineage-positive markers (Ter119^+^/Ly6g^+^/CD11b^+^/B220^+^/CD3e^+^) after 7 days in culture with DMSO or 250 nM RUX (mean ± SEM). Asterisks indicate significant differences as determined by Student *t* tests (∗∗∗*P* < .001; ∗∗*P* < .01; ∗*P* < .05). (D) Schematic diagram showing the serial replating assays that investigated the effect of RUX on ESLAM HSCs isolated from WT and CALRdel/del mutant mice. Sorted ESLAM HSCs were cultured for 7 days in IL-3/IL-6/SCF media^67^ with DMSO or 250 nM RUX and then subjected to serial colony replating assays. (E) Bar plots showing the fold change in the number of colonies produced by HSC-derived cultures treated with vehicle or 250 nM RUX for 7 days, normalized to the number of colonies produced by vehicle-treated cultures at each week of replating. The results are from 2 independent experiments and are shown as mean ± SEM. Asterisks indicate significant differences as determined by Mann-Whitney *U* tests (∗*P* < .05) (F) Schematic diagram showing that HSCs (MPP1–LT-HSCs; CD34^+^CD38^−^CD45RA^−^) cells were sorted from healthy human platelet apheresis donor cone samples or from the peripheral blood of patients with myelofibrosis into 96-well plates (400 cells per well) and cultured in high-cytokine, serum-free medium (EXPER cytokine media)^72^ with scaled doses of RUX or vehicle control (DMSO). After 7 days, the HSC-derived cultures were plated in serial colony replating assays in methylcellulose. Healthy donors were all male and between 48 and 69 years of age. Among the donor patients with myelofibrosis, 3 patients carried a JAK2 V617F mutation and were all male between the ages of 65 to 70 years and 1 donor carried a CALR 52 bp deletion mutation and was a 70 year old female at the time of sample collection. (G) Bar plots showing the fold change in the number of colonies produced by HSPCs that were isolated from healthy donors and cultured for 7 days in the presence of RUX, normalized to the number of colonies that were produced by HSPCs after culturing for 7 days with DMSO. The data are shown as log_2_(fold change) from DMSO. Left showing fold change in colony numbers in the first round of colony formation (2 weeks in methylcellulose). Right showing fold change in colony numbers in the second round of colony formation (4 weeks in methylcellulose). From the data of the 4 healthy donors, each dot represents the mean fold change between technical replicates of a single donor. (H) Table showing the significance values (*P* value) from the estimated marginal (EM) means statistics derived from comparisons between DMSO and RUX conditions using a generalized mixed linear model applied to the raw colony counts used to generate Figure 5G. (I) Bar plots showing the fold change in number of colonies produced by HSPCs that were isolated from patients with myelofibrosis and cultured for 7 days in the presence of RUX, normalized to the number of colonies produced by HSPCs cultured for 7 days with DMSO. The data are shown as log_2_(fold change) from DMSO. Left showing the fold change in colony numbers in the first round of colony formation (2 weeks in methylcellulose). Right showing the fold change in colony numbers in the second round of colony formation (4 weeks in methylcellulose). Each dot represents the average fold change from each of 4 patients with myelofibrosis, and the bars represent the mean ± SEM. (J) Table showing the significance values (*P* value) from EM means statistics derived from comparisons between DMSO and RUX conditions using a generalized mixed linear model statistic applied to colony counts used to generate Figure 5I.

We therefore studied the effect of ruxolitinib on CALR-mutant HSCs derived from a knockin mouse model^58^ that carried a CALR-52 bp deletion mutation commonly observed in human patients with MPN^43^ and known to activate JAK/STAT signaling^71^ (Figure 6A). Ruxolitinib reduced the number of progeny cells generated by CALR-mutant ESLAM HSCs and also the proportion of lineage-positive cells (Figure 6B-C). Ruxolitinib pretreatment also enhanced the replating capacity of cells derived from CALR-mutant ESLAM HSCs (Figure 6D-E; supplemental Figure 6A-B), demonstrating that ruxolitinib maintains clonogenic HSPCs.

To investigate whether ruxolitinib maintained human HSCs in ex vivo cultures, CD34^+^CD38^−^CD45RA^−^ HSPCs were purified from apheresis cones derived from 4 platelet donors, grown in cytokine-rich, serum-free culture conditions^72^ with or without ruxolitinib, and their progeny cells were assessed in serial colony replating assays (Figure 6F). These human cell cultures did not contain albumin, which binds ruxolitinib, necessitating the use of lower ruxolitinib doses as previously described.^73^ After 2 weeks, ruxolitinib did not increase colony formation and even reduced colony output at the highest dose (500 nM), but by 4 weeks, it increased colony formation in all individuals at all doses tested with 10 nM and 50 nM (similar to concentrations obtained in patients in vivo^74^ after accounting for albumin) showing the greatest benefit (Figure 6G-H; supplemental Figure 6C-E; supplemental Table 5).

Ruxolitinib had a similar effect on HSPCs (CD34^+^CD38^−^CD45RA^−^) derived from the peripheral blood of 4 patients with myelofibrosis with high white blood cell counts, none of whom had previously received ruxolitinib or interferon. Three patients were positive for the JAK2V617F mutation, and 1 patient had a CALR deletion mutation. After 2 weeks, ruxolitinib had little effect on the colony output except at the highest dose, but at 4 weeks, it substantially increased the colony output in all 4 patients with 10 nM and 50 nM concentrations showing the greatest benefit (Figure 6I-J; supplemental Figure 6F-H; supplemental Table 6).

Together, these data demonstrate that ruxolitinib maintained cultured murine myeloproliferative HSCs, human normal HSPCs, and human myeloproliferative HSPCs.

## Discussion

Our results demonstrate that STAT5 loss is accompanied not only by reduced HSC numbers but also by a substantial impairment in HSC that was associated with reduced cell cycle entry and increased differentiation. Prompted by this unusual phenotype, we showed that uSTAT5 promotes maintenance and constrains differentiation and proliferation of HSCs. Ruxolitinib, a JAK1/2 inhibitor widely used clinically, increases uSTAT5 levels and enhances the maintenance of WT and myeloproliferative HSCs from both mice and humans.

An intimate relationship between proliferation and differentiation has long been recognized in studies of HSC biology. Many genetic (eg, ablation of CDKi^75^, ^76^, ^77^ or MEK1^78^) or environmental manipulations (eg, infections or inflammation) that induce HSC proliferation and functional exhaustion are associated with increased differentiation.^79^, ^80^, ^81^, ^82^ In contrast, many of those that produce increased HSC quiescence are accompanied by reduced differentiation (eg, Neo1 downregulation^83^ or Atad3a deletion^84^). However, we showed here that highly purified STAT5-deficient HSCs display transcriptional evidence of reduced cell cycling, together with functional evidence of reduced cell cycle entry, and yet are more prone to differentiation. Bunting and colleagues have previously reported that STAT5-deficient LSK or CD34^−^LSK HSCs displayed increased cell cycling.^26^^,^^51^ However, the frequencies of quiescent cells in their WT control populations were lower than those observed in the ESLAM HSCs here (84% vs 91%), suggesting that cell populations gated for cell cycle analysis in the previous reports contained a higher frequency of more proliferative progenitors (presumably ST-HSC/MPP). The decreased frequency of primitive HSCs in STAT5^−/−^ mice likely led to a higher fraction of more proliferative ST-HSC/MPPs, thus increasing the proliferation scores for populations containing such cells.

Our demonstration that STAT5 not only induces HSC proliferation but also represses HSC differentiation was reminiscent of previous results, which showed that uSTAT5 and pSTAT5 have separate transcriptional roles in megakaryocytic differentiation of multipotent HPC7 cells.^55^ We therefore explored the possibility that the functional consequences of STAT5 loss in HSCs might represent a compound phenotype that involves loss of both the uSTAT5 and pSTAT5 transcriptional programs. Two aspects of our studies are of particular note.

First, our results indicate that uSTAT5 constrains HSC differentiation (as shown by both knockout and lentiviral expression approaches) and HSC proliferation and also enhances HSC maintenance as assessed by serial replating and transplantation of STAT5-YF–expressing cells. In the latter studies, STAT5-YF increased donor chimerism within the HSC compartment in both primary and secondary recipients but reduced donor chimerism within whole BM, indicating that STAT5-YF–expressing HSCs are retained in the HSC compartment and are less likely to differentiate. Second, these functional changes reflected altered HSC transcriptional programs including signatures of reduced differentiation, increased quiescence, and increased stemness as assessed by several different scoring systems.

Our results highlight the need to take the signaling environment into account when interpreting the consequences of manipulating a STAT. Thus, using culture conditions that preclude significant STAT5 phosphorylation, the consequences of up- or downregulating STAT5 can be attributed to an effect on uSTAT5. However, we cannot completely exclude potential confounding effects of low levels of endogenous pSTAT5 when assessing the effect of STAT5-YF expression in WT HSCs. Our results also underline the challenges inherent to disentangling the different biologic effects of uSTAT5 and pSTAT5. For example, a requirement for pSTAT5 in replating assays precluded analysis of the ability of STAT5-YF to rescue STAT5-null HSCs. Tools that specifically deplete uSTAT5 are currently lacking but would greatly aid in the dissection of the distinct physiological roles of uSTAT5 and pSTAT5.

It is interesting to consider our results in the light of data that HSCs can be expanded using culture conditions that include high TPO concentrations (100 ng/mL).^85^ This observation is in contrast with other reports that showed that low TPO^86^ and low cytokine environments^87^ better maintain HSC function and that injection of TPO or a TPO mimetic reduces HSC numbers and HSC function in vivo.^88^ Together, these data indicate that the effect of TPO is complex and may be concentration and/or context dependent. TPO-driven HSC expansion may require other features of the Wilkinson expansion cultures (eg, presence of polyvinal alcohol, absence of albumin, hypoxic incubation^85^).

Our results also have therapeutic implications. First, they raise the possibility that ruxolitinib could be a useful strategy to enhance ex vivo maintenance of HSCs for gene therapies. Consistent with this concept, human HSPCs cultured in gene therapy conditions display a rapid upregulation of JAK/STAT signaling, and its inhibition improved their long-term repopulation.^89^ Second, in patients with an MPN,^89^^,^^90^ JAK inhibitors have little if any effect on the level of the mutant clone.^50^ A protective effect of ruxolitinib on mutant HSPCs may contribute to the limited efficacy of JAK inhibitors. Moreover, an accumulation of mutant HSCs poised to differentiate may also contribute to the JAK-inhibitor discontinuation syndrome, characterized by a rapid life-threatening MPN resurgence after JAK-inhibitor withdrawal.^90^ The cytokine environments of endogenous HSCs in their various niches remain poorly understood and so further studies will be needed to explore the HSC effects of ruxolitinib in vivo. However, our results raise the possibility that targeting uSTAT5 or total STAT5 activity may represent attractive therapeutic approaches for myeloid malignancies associated with JAK activation.

Conflict-of-interest disclosure: A.R.G. and J.L. report serving as consultants for Incyte. E.L. reports receiving research funds from GlaxoSmithKline and Commonwealth Serum Laboratories 10.13039/100008322Behring. The remaining authors declare no competing financial interests.

## Supplementary Material

Supplemental Tables 1-6

Supplemental Methods, Figures, Legends for Tables, and References
