# Supplementary material for: Maintenance of hematopoietic stem cells by tyrosine-unphosphorylated STAT5 and JAK inhibition
Source: Blood Adv. 2024 Oct 10;9(2):291–309. doi: 10.1182/bloodadvances.2024014046 (PMC7617191; doi:10.1182/bloodadvances.2024014046)
Supplement: Supplemental Methods, Figures, Legends for Tables, and References [file BLOODA_ADV-2024-014046-mmc2.pdf]

Supplemental data for Williams et al.

- Supplemental methods
- Supplemental Figures SF1-SF6
- Supplemental Tables ST1-ST6
- References

Supplemental Methods

Poly:IC treatment

Poly:IC (γ-irradiated sodium salt, Sigma) was prepared in PBS and administered intraperitoneally (IP) to $STAT5^{fl/fl}Cre^{+/-}$ or $STAT5^{fl/fl}Cre^{-/-}$ mice (16mg/kg) up to 7 times over 21 days. Peripheral blood (PB) was collected via tail vein into EDTA (Ethylenediaminetetraacetic acid) coated microvette tubes (Sarstedt) at least 4 weeks after the final IP injection. Blood mononuclear cell DNA was extracted at room temperature (RT) for 5 minutes with diluted NaOH. After pH neutralization, a genomic PCR was set up to test the level of STAT5 recombination in peripheral blood.

Bone marrow cell harvest:

Tibia, femur and iliac crest bones from both hind legs were crushed in a mortar with FACS buffer (Calcium free and Magnesium free PBS; Gibco) containing 2%FBS (Gibco) and 5mM EDTA) and filtered through a 70-um strainer to obtain single cell suspensions. Equal volume of ammonium chloride solution (STEMCELL Technologies) was mixed with cell suspension gently and incubated for 10 min at 4°C to lyse red blood cells. Cells were then spun at 360 xg for 5 min, and bone marrow mononuclear cells (BMMNCs) were washed in 40mL FACS buffer, and then used for analyses.

Flow cytometric analysis

Washed BMMNCs were resuspended in an appropriate volume for antibody staining. The BMMNC were incubated with the appropriate dilution of antibodies with fluorescent conjugates. Cells were washed in FACS buffer and then stained

with 4’,6-diamidino-2-phenylindole (DAPI, Thermo Fisher) or 7AAD (Biolegend) for dead cell exclusion in FACS buffer. Stained cells were then analysed on a LSR Fortessa flow cytometer (BD Biosciences) equipped with FACSDiva Software. Data were analyzed using FlowJo (Tree Star).

The frequency of HSCs, progenitors and lineage cells in bone marrow was analyzed by flow cytometry: T cells, CD3e^{+}; B cells, B220^{+}; pro- and erythroblasts, defined by different levels of CD71 and Ter119 as in the figure legend; Monocytes/neutrophils, Ly6G^{+}; Granulocytes/macrophages, Mac-1^{+}; MK, CD41^{+}CD42^{+}; prog, Lin^{-}Sca1^{-}cKit^{+}; megakaryocyte progenitors (MkP), Lin^{-}Sca1^{-}cKit^{+}CD150^{+}CD41^{+}; GMP, Lin^{-}Sca1^{-}cKit^{+}CD41^{-}CD16/32^{+}CD150^{-}; PreCFU-e, Lin^{-}Sca1^{-}cKit^{+}CD41^{-}CD16/32^{-}CD105^{+}CD150^{+}; CFU-e, Lin^{-}Sca1^{-}cKit^{+}CD41^{-}CD16/32^{-}CD105^{+}CD150^{-}; PreMegE, Lin^{-}Sca1^{-}cKit^{+}CD41^{-}CD16/32^{-}CD105^{-}CD150^{+} and PreGM, Lin^{-}Sca1^{-}cKit^{+}CD41^{-}CD16/32^{-}CD105^{-}CD150^{-}. Multipotent progenitor MPPs were defined as the following: MPP1 (Flk2^{-}CD150^{+}CD48^{-}LSK), MPP2 (Flk2^{-}CD150^{+}CD48^{+}LSK), MPP3 (Flk2^{-}CD150^{-}CD48^{+}LSK) and MPP4 (Flk2^{+}CD150^{-}CD48^{+}LSK). LT-HSC was defined as Flk2^{-}CD34^{-}Lin^{-}Sca1^{+}cKit^{+}CD150^{+}CD48^{-} and ESLAM HSCs as CD45^{+}EPCR^{+}CD150^{+}CD48^{-}. CD45.1 and CD45.2 antibodies were used to distinguish donor, recipient and competitor cell populations in transplants. All antibodies were produced by Biolegend, except for anti-EPCR PE conjugated antibodies (STEMCELL Technologies), and anti-CD45.1 and anti-CD45.2 (BD Bioscience).

FACS isolation of ESLAM HSCs

Single cell suspensions of BMMNCs were first lineage depleted using EasySep™ Mouse Hematopoietic Progenitor Cell Isolation Kit (STEMCELL Technologies). Lineage depleted cells were then stained with; EPCR (CD201) PE (clone RMEPCR1560, STEMCELL Tech), CD150 PE/Cy7 (clone TC15-12F12.2, BioLegend), CD45 FITC (clone 30-F1,1 BD Bioscience) and CD48 APC (clone HM48-1, Biolegend). ESLAM HSCs defined as $CD45^{+}EPCR^{+}CD48^{-}CD150^{+}$ as previously described,[1] were isolated using a Becton Dickinson Influx sorter (BD Biosciences). Cells were sorted directly into culture dishes containing media or into Eppendorfs containing FACS buffer.

Competitive transplantation assays

C57BL/6 (CD45.1) recipients were irradiated with 2 x 550 cGy. For competitive repopulation assays, ESLAM HSCs or GFP^{+} transduced HSCs ($CD45.2^{+}$) were injected with 3-5 x 10^5 competitor bone marrow cells obtained from CD45.1/CD45.2 F1 mice into recipient mice. To evaluate qualitative differences of WT and $STAT5^{-/-}$ HSCs, 30 FACS isolated ESLAM HSCs were mixed with 3 x 10^5 competitor bone marrow cells obtained from CD45.1/CD45.2 F1 mice before being injected into CD45.1 recipient mice. Blood was analyzed every 28 days. At 6 months post transplantation, bone marrow cells from the primary recipient mice were assessed for donor-derived HSC chimerism using flow cytometry by staining bone marrow cells with LT-HSC ($Lin^{-}Sca1^{+}cKit^{+}CD150^{+}CD48^{-}CD34^{-}Flk2^{-}$) as well as CD45.1 and CD45.2. Secondary transplantation was then performed using 5 x 10^6 nucleated bone marrow cells from primary recipients of ESLAM HSCs.

To evaluate the long-term functional capacity of uSTAT5B overexpressing HSCs, WT ESLAM HSCs ($CD45.2^+$) were FACS sorted and transduced with lentivirus containing STAT5B-YF, or EV in SCF/IL-11 cultures[2], and three days later 113 transduced donor cells were transplanted per recipient mouse ($CD45.1^+$) with 3 x 10^5 competitor bone marrow cells obtained from CD45.1/CD45.2 F1 mice. Blood was analyzed every 28 days for 6 months before secondary transplants were set up by injecting 5 x 10^6 primary recipient bone marrow cells into irradiated secondary recipient mice. Blood was analyzed every 28 days for 6 months before harvesting. Donor-derived HSC chimerism was assessed using flow cytometry by staining bone marrow cells with ESLAM HSC panels as described above, as well as antibodies for CD45.1 and CD45.2.

To evaluate the effect of ruxolitinib treatments on HSC function, ESLAM HSCs ($CD45.2^+$) were pre-cultured for 5 days in IL-3/IL-6/SCF culture conditions[3] with vehicle or ruxolitinib before being injected with 3 x 10^5 competitor bone marrow cells obtained from $CD45.1^+$ mice into irradiated recipient mice ($CD45.1^+$/$CD45.2^+$). Blood was analyzed every 28 days for 6 months before harvesting. Donor-derived HSC chimerism was assessed using flow cytometry by staining bone marrow cells with ESLAM HSC panels as described above, as well as antibodies for CD45.1 and CD45.2. As ESLAM HSCs differentiate in 3-day or 5-day cultures but retain cell types with competitive repopulating capacity we refer to them as ‘HSCs’ in the main text after the culture period.

In all cases, peripheral blood was obtained and analyzed by flow cytometry for donor contribution to T cells ($CD3e^+$), B cells ($B220^+$), Monocytes/neutrophils

($Ly6G^+$); Granulocytes/macrophages ($Mac\text{-}1^+$). Cells were co-stained with antibodies for CD45.1 and CD45.2 to distinguish the donor origin of repopulated cells.

ESLAM HSC *ex vivo* cultures

ESLAM HSCs were sorted from $STAT5^{-/-}$ or WT mouse bone marrow cultured as single cells in round-bottom 96-well plates (Corning) or in bulk cultures of >50 cells per well in flat bottom 96-well plates (Corning). Each well was preloaded with StemSpan SFEM (serum-free expansion medium, STEMCELL Technologies). Cell suspensions were then topped up with equal volume of StemSpan SFEM media containing cytokines as indicated below after cell sorting was completed. Cells were cultured in 37°C, 5% CO_2. **IL-11 and SCF cultures:**[2] Equal volume of medium was added to each well to a final concentration of 10% FBS (STEMCELL Technologies), 1% penicillin/streptomycin (Sigma-Aldrich), 1% L-glutamine (Sigma-Aldrich), stem cell factor (SCF; 250 ng/mL; STEMCELL Technologies), IL-11 (20ng/mL; STEMCELL technologies) and 0.1 mM β-mercaptoethanol (Gibco). **IL-3/IL-6 and SCF cultures:**[3] Equal volume of medium was added to each well to a final concentration of 10% FBS (STEMCELL Technologies), 1% penicillin/streptomycin (Sigma-Aldrich), 1% L-glutamine (Sigma-Aldrich), stem cell factor (SCF; 250 ng/mL; STEMCELL Technologies), IL-3 (10 ng/mL; PeproTech), IL-6 (10 ng/mL; PeproTech) and 0.1 mM β-mercaptoethanol (Gibco).

Single cell *in vitro* division kinetic assays

Single $STAT5^{-/-}$ or WT ESLAM HSCs were sorted at single cell into round bottom 96-well plates (Corning). Cells were cultured in 100μL IL-11 and SCF culture

media[2]. Cell counts were scored daily under microscope, and cell cycle kinetics was determined as described (Li et al 2018) for the first, second, third and fourth division based visually scored cell numbers. First division (≥2 cells per well), second division (≥3 cells per well), third division (≥5 cells per well), or fourth division (≥8 cells per well).

Bulk ESLAM HSC *in vitro* expansion and differentiation analysis

50 STAT5$^{-/-}$ or WT ESLAM HSCs were sorted into flat-bottom 96-well plates (Corning). For STAT5-deficient HSC expansion and differentiation analysis at days 4, 5, 6 and 10, cells were cultured in IL-11 and SCF conditions.[2] For ruxolitinib treated HSC differentiation and expansion analysis, cells were placed in IL-3, IL-6 and SCF cultures conditions[3] for 2h and then ruxolitinib was applied in equal volume to final concentrations indicated in figures and cultured for 7 days. Cells were harvested at indicated time points and were then washed with FACS buffer and resuspended in an appropriate volume of FACS buffer for antibody staining. Cells were stained with fluorescently labelled antibodies against: Ly-6G BV785 (Biolgend), CD11b AF700 (Biolegend), Ter119 PE (Biolegend), B220 FITC (Biolegend), CD3e FITC (Biolegend), CD19 FITC (Biolegend), NK1.1 APC (Biolegend), c-Kit APC/Cy7 (Biolegend), Sca1 BV605 (Biolegend). Alternatively cells were stained with Lineage cocktail AF700 (Biolegend), which contained anti Ter119, CD3e, B220 Ly6G and CD11b antibodies. Cells were washed in FACS buffer and then stained with 4’,6-diamidino-2-phenylindole (DAPI, Thermo Fisher) or 7AAD (Biolegend) for dead cell exclusion in FACS buffer. Stained cells were then analyzed on LSRFortessa flow cytometer (BD Biosciences) equipped with FACSDiva Software. Data were analyzed using FlowJo (Tree Star). Accurate cell

counts were acquired by applying a defined number of fluorescent beads (Trucount Control Beads, BD Biosciences) to each well. Each sample was back-calculated to the proportion of the total that were run through the cytometer.

Analysis of STAT5B-YF transduced ESLAM HSC single cell cultures

Transduced (GFP^+) $STAT5^{-/-}$ or WT ESLAM HSCs were sorted 3 days post infection at single cell per well in round-bottom 96-well plates (Corning) and cultured in IL-11 and SCF media[2] for a further 7 days. Cells were harvested and washed with FACS buffer and resuspended in an appropriate volume of FACS buffer for antibody staining and analyzed for lineage positive cell frequency and cell number. Stained cells were then analyzed on LSRFortessa flow cytometer (BD Biosciences) equipped with FACSDiva Software. Data were analyzed using FlowJo (Tree Star). Accurate cell counts were acquired by applying a defined number of fluorescent beads (Trucount Control Beads, BD Biosciences) to each well. Each sample was back-calculated to the proportion of the total that were run through the cytometer.

Lentiviral infection of ESLAM HSCs

1,000-20,000 ESLAM HSCs from $STAT5^{-/-}$ or WT mice were sorted and split across wells of a 96 well plate (Corning). Cells were cultured in 50μL of IL-11 and SCF media[2] for 2h before being supplemented with polybrene (Sigma-Aldrich) and up to 20uL of a lentivirus equipped with the gene of interest (STAT5B-Y699F) driven by MND promoter in a pCCL-c-MNDUS-IRES-EGFP backbone. The following morning cells were replated in 12 well plates in 600ul to dilute the polybrene and virus. Three days after infection, cells were sorted for living ($DAPI^-$, Thermo Fisher)

and green fluorescent protein (GFP^+) and used in *in vitro* assays and transplantation experiments.

Lentivirus production

Lentiviral production was performed with HEK Lenti-X 293T cells (Takara) in low glucose Dulbecco's Modified Eagle's Medium with GlutaMAX and sodium pyruvate (DMEM, Thermo Fisher), supplemented with 10% heat-inactivated FBS (Sigma-Aldrich), 1% penicillin/streptomycin (Sigma-Aldrich). HEK Lenti-X 293T cells were split 24 hours before production protocol began.

HEK Lenti-X 293T cells were plated at 9 million cells in 20mL media in a 15cm dish (Corning) and cultured for 24h. Two hours before transfection with the plasmids, media was replaced with 18mL fresh media. 2ml of DMEM containing 3rd generation lentiviral packaging plasmids (12µg, Takara), Lipofectamine (3% f.c. Thermo Fisher), and plasmid of interest (12µg, pCCL-c-MNDUS-IRES-EGFP containing EV, STAT5B-WT or STAT5B-Y699F, were added to dishes while swirling. Plates were incubated over-night and media was changed the following morning. 48h after transfection, media was collected and filtered through 0.45µm syringe filters (Falcon). Virus was concentrated with Lenti-X concentrator (Takara) according to manufacturer's instruction. 20µl aliquots were snap frozen and then stored at -80°C.

Mouse HSPC serial colony replating assays

For all serial colony replating assays, cells were seeded into M3434 methylcellulose (STEMCELL Tech) without antibiotic in STEMvision 6-well plates

and were allowed to grow for 1 week before imaging the plate on STEMvision (STEMCELL Technologies). The entire well contents of each condition was harvested and a fraction of cells were seeded into M3434 methycellulose and were allowed to grow for 1 week before imaging. This protocol was performed until colonies were no longer seen across all conditions. Colony Forming Unit (CFU) was defined here as the number of colonies observed, multiplied by the dilution factor (which gets greater with each passing week), divided by the starting number of HSC cells.

For uSTAT5B analysis; transduced $STAT5^{-/-}$ or WT ESLAM HSCs ($DAPI^{-}GFP^{+}$) were sorted 3 days post infection and were seeded at 75 cells per well and split over two duplicate wells. For ruxolitinib analysis; 50 WT ESLAM HSCs were placed into flat bottom 96 well plates (Corning) in SCF, IL-3 and IL-6 cultures.[3] After 2 hours, cultures were treated with DMSO or ruxolitinib (250nM and 1000nM) for 7 days. Diluted cultured cells were then plated in methylcellulose.

Cell cycle analysis of ESLAM HSCs

Bone marrow cells from $STAT5^{-/-}$ and WT control mice were lineage depleted using EasySep™ Mouse Hematopoietic Progenitor Cell Isolation Kit (STEMCELL tech). The cells were then stained for 45 minutes with CD45 BV785 (Biolegend), EPCR PE (STEMCELL Technologies), CD150 PE/Cy7 (Biolegend), CD48 BV605 (Biolegend) and Zombi Nir (Biolegend) to eliminate dead cells. The stained cells were washed with FACS buffer (PBS/2% FBS), then fixed and permeabilized using Cytofix/Cytoperm™ Fixation/Permeablization kit (BD Biosciences). The cells were then stained with Ki-67 FITC (Biolegend) on ice for 1 hr and then with DAPI

(Thermo Fisher) for 1 hr at RT. Flow cytometric analysis was carried out at a low flow rate using LSRFortessa flow cytometer (BD Biosciences).

Intracellular flow cytometry analysis of cultured ESLAM HSCs

ESLAM HSCs from STAT5$^{-/-}$ and WT control mice were sorted and placed into StemSpan SFEM media (STEMCELL technologies) for 1h (cytokine starvation). For intracellular flow analysis of STAT proteins, ruxolitinib or DMSO was added to StemSpan SFEM during the 1h starvation period. Cells were then stimulated with IL-11/SCF cultures,[2] cytokines present in M3434, or IL-3/IL-6/SCF cultures[3] for 30minutes. Cells were then fixed and permeabilized using Cytofix buffer and Perm Buffer III (BD Biosciences). The cells were then stained with anti-pY694/Y699 STAT5 PE antibody (Clone 47/Stat5(pY694), BD Bioscience) on ice for 1 hr. In Ruxolitinib experiments, cells were additionally stained with antibodies for pY701 STAT1 AF488 (Clone 4a, BD Bioscience), pY705 STAT3 AF647 (Clone 4/P-STAT3, BD Bioscience), and total STAT5 (Clone D2O6Y, Cell Signalling Technology) followed by staining with a PE anti-rabbit secondary antibody. STAT5-deficient cells were used a negative control for total-STAT5 antibody staining. Flow cytometric analysis was carried out on a LSR Fortessa flow cytometer (BD Biosciences) equipped with FACSDiva Software. Data were analyzed using FlowJo (Tree Star).

Intracellular flow cytometry analysis of cultured HSPCs with STAT5-YF expression.

HSPCs (LSK cells) from WT mice were sorted and placed into IL-11/SCF cultures,[2] for two hours before lentiviral transduction (see above). 3 days later, GFP+ cells were FACS isolated and placed into placed into StemSpan SFEM media

(STEMCELL technologies) for 1h (cytokine starvation). Cells were then stimulated with IL-11/SCF cultures.[2] Cells were then fixed and permeabilized using Cytofix buffer and Perm Buffer III (BD Biosciences). The cells were then stained with anti-pY694/Y699 STAT5 PE antibody (Clone 47/Stat5(pY694), BD Bioscience) or total STAT5 (Clone D2O6Y, Cell Signalling Technology) followed by staining with a PE anti-rabbit secondary antibody on ice. Flow cytometric analysis was carried out on a LSR Fortessa flow cytometer (BD Biosciences) equipped with FACSDiva Software. Data were analyzed using FlowJo (Tree Star).

Intracellular KI67/DAPI flow cytometry analysis of cultured HSPCs with STAT5-YF expression or ruxolitinib treatment.

STAT5-YF: ESLAM HSCs from WT mice were sorted and placed into IL-11/SCF cultures for 2h before lentivirus transduction with EV or STAT5-YF. Cells were allowed to expand for 5 days before $GFP^{+}DAPI^{-}$ cells were sorted. $GFP^{+}DAPI^{-}$ cells were immediately fixed and permeabilized using Cytofix/Cytoperm™ Fixation and Permeablization kit (BD Biosciences).

Ruxolitinib: ESLAM HSCs from WT mice were sorted and placed into IL-3/IL-6/SCF cultures. Cells were harvested 18h or 5-days later and were immediately fixed and permeabilized using Cytofix/Cytoperm™ Fixation and Permeablization kit (BD Biosciences).

Fixed and permeobolised cells were stained with Ki-67 APC (Biolegend) on ice for 1 hr and then with DAPI (Thermo Fisher) for 1 hr at RT. Flow cytometric analysis was carried out at a low flow rate using LSR Fortessa flow cytometer (BD Biosciences).

Apoptosis analysis of lineage depleted bone marrow

Bone marrow cell suspensions were mixed with equal volume of ammonium chloride (StemCell Technologies) and incubated on ice for 10 minutes to lyse red blood cells. Cells were then washed in 40ml of FACS buffer (PBS/2% FBS 5mM EDTA) and then lineage depleted using EasySep™ Mouse Hematopoietic Progenitor Cell Isolation Kit (STEMCELL technologies). Lineage depleted cells were cultured in 1.5mL IL-3, IL-6 and SCF culture in 6 well plates. After 2h in culture, 1.5mL media containing ruxolitinib (at 2x concentration of the indicated doses) was added to wells and cultured over-night. Cells were then washed with FACS buffer and resuspended in an appropriate volume of FACS buffer for antibody staining. Cells were stained with c-Kit APC/CY7 (Biolegend) and Lin cocktail AF700 (Biolegend) for 45 minutes. The stained cells were washed with FACS buffer, then stained with 5ul Annexin V FITC (Biolegend) in 100µL 1x Annexin binding buffer at room temperature in dark for 15 minutes. DAPI (Thermo Fisher) was then added to the samples. Flow cytometric analysis was carried out using LSRFortessa flow cytometer (BD).

Clone survival analysis of single HSC in *in vitro* culture

Single WT HSCs or $GFP^{+}DAPI^{-}$ transduced cells were seeded into round bottom 96 well plates (Corning) in IL-3, IL-6, and SCF conditions[3] for ruxolitinib treatment analysis of clone viability or in IL-11 and SCF conditions[2] for uSTAT5B overexpression analysis of clone viability. Ruxolitinib was applied after a 2h rest period in culture as above. Wells were studied under a microscope after 5 days. The number of wells carrying 1 or more cells were marked as surviving clones. The frequency of surviving clones from the total clones plates was calculated.

qPCR analysis

WT HSCs were cultured in SCF IL-3 and IL-6 conditions[3] with ruxolitinib or DMSO for 2h before being collected and washed. RNA was extracted using PicoPure RNA isolation kit (Thermo Fisher) as per manufacturer's instruction. First strand cDNA synthesis was performed using SuperScript III First-Strand Synthesis System for RT-PCR (Thermo Fisher), using Random Hexamers, as per the manufacturer's guidelines. QPCR was carried out using PowerTrack™ SYBR Green Master Mix (Thermo Fisher) and the primers below. qPCR assays were run and analysed using Stratagene Mx3000P qPCR System. The expression of individual genes relative to a housekeeping gene (Gapdh) was calculated as R = 2-(CT target – CT housekeeping).

Cish:

Forward primer AGGGATCTTGTCCTTTGCTGG
Reverse primer AGGGTCTAGCACCTTCGGTT

Pim1:
Forward primer CAAGATCAACTCCCTGGCCC
Reverse primer TTAATGGCCACCGGCAAGTT

HEL and BaF3 cell culture and western blot

All cells were cultured in 37°C, 5% CO_2. HEL cells carrying a $JAK2^{V617F}$ mutation were cultured in RPMI-1640 media (Thermo Fisher) with 10% FBS (Gibco) and 1% penicillin/streptomycin (Sigma-Aldrich). Ba/F3 cells that overexpress c-MPL and mutant calreticulin (CALR:del) were cultured in RPMI-1640 media (Thermo Fisher) with 10% FBS (Gibco) and 1% penicillin/streptomycin (Sigma-Aldrich) with 25ng/mL of THPO (Thermo Fisher).

1 x 10^6 cells were plated into 6cm dishes. 24h later cells were treated for 2h with ruxolitinib at 1000nM, 250nM or DMSO. Cells were washed with cold PBS (Gibco) and harvested in dishes on ice with RIPA buffer (Thermo Fisher) supplemented with protease and phosphatase inhibitors (Merck). Blots were stained with anti-STAT5 (D2O6Y, Cell Signaling), anti-pY694-STAT5 (ab32364, Abcam) and anti-tubulin (2144, Cell Signalling) in Tris buffered saline (TBS, Thermo Fisher) with 0.1% Tween and 5% BSA. Blots were stained with secondary HRP linked antibodies (Thermo Fisher) in TBS with 0.1% Tween and 3% milk powder (Marvel).

Statistical Analysis

For all comparisons of datasets where there was ≥3 replicate samples tested, an unpaired Student's t-test was tested in Graphpad (Prism) where all bar charts, dot plots and line graphs were plotted. The level of significance was indicated with asterisks. For comparing between conditions across multiple time points (STAT5$^{-/-}$ and WT cohort blood analysis, or STAT5$^{-/-}$ and WT competitive transplants blood repopulation) an ANOVA was performed and the column factor p value was indicated.

Generation of STAT5$^{-/-}$ and WT ESLAM cells Smart-seq2 scRNAseq dataset

Bone marrow cells were harvested from STAT5$^{-/-}$ and WT control mice and single ESLAM HSCs were FACS sorted after lineage depletion, as described above, and RNA libraries were made according to manufacturer's protocol (Illumina) processed by as described for Smart-seq2.

Preprocessing Smart-seq2 data

The reads resulting from the SmartSeq2 experiments were mapped against Ensembl genes (release 81)[4] using GSNAP (version 2015-09-29)[5] and quantified using HTSeq (version 0.6.0).[6] Filtering Quality Control (QC) steps were applied where nuclear genes had to be at least 20% of the mapping reads and cells with less than 50,000 reads mapping to them were rejected. In addition, the maximum allowed fraction of cells mapping to mitochondrial genes was set at 20%. The levels of technical variance were estimated using the ERCC spike-ins as described by Brennecke *et al.* (2013)[7] with highly variable genes (HVGs) being defined as having the squared coefficient of variation exceeding technical noise. The resulting dataset was also transformed by applying the remove batch effect method implemented within the R limma package.[8]

Additional processing and filtering Smart-seq2 data

Further processing of the dataset was performed with the python package Scanpy.[9] The dataset was subsequently log-transformed, scaled and using the HVGs as input a PCA reduction for the top 50 components was computed. A diffusion map embedding was also calculated for the top 15 diffusion components. While considering the geometric mean of Stat5a/Stat5b expression 23 HSCs from STAT5 deficient mice were excluded from further analysis. These excluded cells corresponded to the top 20% *Stat5a* and *Stat5b* expressing HSCs from the STAT5 deficient mice.

Analysis of differential expression genes of the Smart-seq2 dataset

Differential expression on the Smart-seq2 sequencing data was performed using the R package DESeq2.[10] The input expression matrices were filtered to include

only genes with average expression above 1 (resulting in 14,080 genes on the filtered matrix). Differential expression results were selected for a significance level of 0.01. Visualization of results was done via volcano plot generated with the Enhanced Volcano R package.[11]

Gene set enrichment analysis of the Smart-seq2 data

The Wald statistic from DESeq2 result was used to generate pre-ranked gene lists. These gene lists were then used for Gene set enrichment analysis (GSEA) with the GSEA software (v3.0) pre-ranked mode. Enrichment was tested for Hallmark and KEGG curated genesets of the MSigDB database v7.0.[12]

Generation of 10x scRNAseq dataset of STAT5$^{-/-}$ and WT

Bone marrow cells were harvested from STAT5KO and WT control mice and lineage negative, c-Kit^{+} (LK) cells were FACS sorted and processed according to the manufacturer's protocol for the 10x Chromium™ Single Cell 3' kit (v2)(10x Genomics, Pleasanton, CA). There were six samples in total, including 2 replicates for 3 months WT samples, 2 replicates for 3 months Stat5KO samples and 1 sample each for 12 months WT and Stat5KO, respectively. The prepared libraries were sequenced on a Novaseq 6000. The sequenced reads were processed with Cellranger (version 2.1.1) and aligned to the 10X Genomics built mouse mm10 reference (version 1.2.0).

STAT5$^{-/-}$ and WT LK 10X data pre-processing

The Scrublet package was used to estimate doublets and 449 were selected and removed as such. The cells were further filtered based on the percentage of

mitochondrial UMI counts being less than 10% per cell total and cells expressing at least 500 genes. Genes were remained if they were expressed in at least in 3 cells. The filtered dataset was subsequently normalized to the median UMI counts per cell across the two processed libraries and log-transformed.

Downstream analysis of the STAT5$^{-/-}$ and WT LK 10X scRNAseq dataset

After the preprocessing step, there were 30728 cells past quality control (3m WT S1: 5708; 3m WT S2: 4943; 3m Stat5KO S1: 4469; 3m Stat5KO S2: 4596; 12m WT: 5239 and 12m Stat5KO: 5773). The following downstream analysis was performed using the Scanpy package in Python. Cell cycle phases were estimated using the score_genes_cell_cycle function in Scanpy with the default 97 cell cycle genes defined in Tirosh et al, 2015. Highly variable genes were then computed with parameters *min_mean*=0.02, *max_mean*=3, *min_disp*=0.3 within each batch separately and merged. The effect of cell cycle phases, number of genes, number of total counts and percentage of mitochondrial genes were removed using the regress_out function in Scanpy. The dataset was scaled prior to the principle component analysis (PCA) calculation. Uniform Manifold Approximation and Projection (UMAP), a dimension reduction technique was used to visualise the data using the top 50 PCs.

Generation of 10X scRNAseq dataset of STAT5B-YF ESLAM HSC *in vitro* culture

ESLAM HSCs were sorted as CD45^{+}EPCR^{+}CD150^{+}CD48^{-} and transduced with lentivirus containing STAT5B-YF or EV, respectively, in SCF and IL-11 cultures.

Five days after infection, $DAPI^-$ GFP^+ were sorted for 10X scRNAseq. There were 2 replicates for each condition. The cells were processed with a Chromium Controller according to the Chromium NextGEM Single Cell 3' GeneExpression v3.1 protocol. The libraries were pooled and sequenced on a Novaseq 6000.

Pre-processing of the 10X scRNAseq dataset of STAT5B-YF or EV transduced ESLAM HSC *in vitro* culture

The scrublet package in Python was used to estimate doublets and cells estimated to potentially be doublets were removed. In addition, cells with less than 1000 genes, or more than 2e5 UMI counts or more than 5% mitochondrial gene counts were further eliminated to get best quality cells for downstream analysis.

Downstream analysis of the 10X scRNAseq dataset of STAT5B-YF ESLAM HSC *in vitro* culture

After the pre-processing step, there were 21649 cells remaining (629; YF B1: 494; EV B1: 629; YF B2: 3945 and EV B2: 10149). Highly variable genes were then computed with parameters *min_mean*=0.02, *max_mean*=3, *min_disp*=0.5 within each batch separately and merged. The effect of cell cycle phases, number of genes, number of total counts and percentage of mitochondrial genes were removed using the regress_out function in Scanpy. The dataset was scaled prior to the principle component analysis (PCA) calculation. Uniform Manifold Approximation and Projection (UMAP), a dimension reduction technique was used to visualise the data using the top 50 PCs.

Generation of 10x scRNAseq dataset of Ruxolitinib treated ESLAM HSC culture

ESLAM HSCs were sorted and cultured in the medium that contains SCF, IL3 and IL6 to promote STAT5 phosphorylation with DMSO or ruxolitinib (250nM) for 5 days. After that, DAPI^{-} cells were sorted for scRNAseq. There were 2 replicates for each condition. The cells were processed with a Chromium Controller according to the Chromium NextGEM Single Cell 3' GeneExpression v3.1 protocol. The libraries were pooled and sequenced on a Novaseq 6000. The sequenced reads were processed with the cellranger pipeline (version 3.1.0) and aligned to the genome reference 10X Genomics-builqd mouse mm10 (version 3.0.0).

Pre-processing of the ESLAM HSC culture treated with DMSO or Ruxolitinib

The Scanpy (v1.9.6) package in Python (v3.9.18) was used for data pre-processing. The scrublet package (v0.2.3) in Python was applired to estimate doublets and cells estimated to potentially be doublets were removed. In addition, cells with less than 500 genes, or more than 2e5 UMI counts or more than 5% mitochondrial gene counts were further eliminated to get best quality cells for downstream analysis.

Downstream analysis of the 10x scRNAseq dataset of Ruxolitinib treated ESLAM HSC culture

After the pre-processing step, there were 19565 cells remaining (DMSO_rep1: 11428, DMSO_rep2:4972, Rux_rep1:1123, Rux_rep2:2042). The cells were normalized to a total count of 1e4. Highly variable genes were then computed with parameters min_mean=0.02, max_mean=3, min_disp=0.5 within each batch separately and merged. The effect of cell cycle phases, number of genes, number

of total counts and percentage of mitochondrial genes were removed using the regress_out function in Scanpy. Cell cycle phases were estimated using the score_genes_cell_cycle function in Scanpy. After that, the cell cycling effect was further eliminated using the regress_out function. Harmony (v1.2.3360) was then applied to correct potential batch effects between samples. The dataset was scaled prior to the principle component analysis (PCA) calculation. Umap was used to visualise the data using the top 50 PCs. Cell types were annotated using the published Nestorowa [PMID:27365425] and Dahlin [PMID:29588278] landscapes. Leiden clustering with resolution=0.8 defined 15 clusters, of which cluster 10-12 were annotated as non-HPSC cells and hence were removed for further analysis. Differential expression analysis was carried out for each cell type between Rux-treated and DMSO-control using the edgeR package (v4.0.5) in R (v4.3.1). Pre-filter on genes was done using min.count=1, min.total.count=1, large.n=10 and min.prop=0.3. All the plots were created using ggplot2 (v3.4.4) in R.

10X data projection and cell type annotation

For STAT5$^{-/-}$ and STAT5$^{+/+}$ HSPC datasets, uSTAT5B overexpressing HSC culture datasets, and ruxolitinib treated HSC culture datasets; in order to acquire the cell type information for each cell, the cells from all the samples (done individually for each batch of each experiment) were projected onto 2 different pre-annotated reference landscapes: 1) Dahlin landscape consists of both LSK and LK populations[13] and 2) Nestorowa landscape with mainly hematopoietic stem and progenitor populations.[14] Highly variable genes from the reference landscape were used for the PCA calculation. New data was projected onto the reference landscapes using PCA projection. The 15 nearest neighbor cells from the

reference landscape were calculated for each cell of the new data based on Euclidean distance and the cell type annotation was assigned as the most frequent cell type out of the 15 matched nearest neighbours. As the Nestorowa landscape has a more detailed annotation on immature populations whereas Dahlin landscape has a more detailed annotation on more mature populations, for the final cell type assignment, the Dahlin landscape was used as the key reference, from which the cells that were annotated as HSC or immature populations were replaced by the annotations from the Nestorowa landscape.

Visualizations

All violin plots were done using the ggplot2 package in R. Mean difference between groups were tested using t test.

Human samples from healthy donors

Non mobilized peripheral blood samples were collected from leukocyte cones (a by-product of platelet apheresis) with informed consent by NHS Blood and Transfusion (NHSBT) Centres and sourced by the Cambridge Blood and Stem Cell Biobank. All experimental work was performed in accordance with regulated procedures approved under the 07/MRE05/44 and 18/EE/0199 REC research studies.

Human samples from myelofibrosis patients

Non mobilized peripheral blood samples were collected from the peripheral blood of myelofibrosis (MF) patients, all with informed consent by NHS Blood and Transfusion (NHSBT) Centres and sourced by the Cambridge Blood and Stem Cell

Biobank. All experimental work was performed in accordance with regulated procedures approved under the 18/EE/0199 REC research studies. All patients (n=4) had white blood cell counts greater than $10x10^9$/L and had not received ruxolitinib or interferon as part of their medical treatment prior to sample collection. 3 donors were diagnosed with JAK2V617F mutant MF and one patient was diagnosed with CALR-Del mutant MF.

Human serial colony replating

Non mobilized peripheral blood HSC pool cells ($CD34^+CD38^-CD45RA^-$) were isolated from healthy donors or MF patients and seeded at 400 cells per well in 96 well plates and cultured in conditioned media termed "MEM" media.[15] MEM media: StemPro base media supplemented with Nutrients (0.035%), Pen/Strep (1%), L-Glu (1%), human LDL (50ng/ml), SCF (100ng/ml), Flt-3L (20ng/ml), TPO (100ng/ml), EPO (3 units/ml), IL-6 (50ng/ml), IL-3 (10ng/ml), GM-CSF (20ng/ml), IL-11 (50ng/ml), IL-2 (10ng/ml) and IL-7 (40ng/ml). All cytokines from Miltenyi Biotech except EPO (Janssen). Two hours , RUX (5nM, 10nM, 50nM or 500nM, Selleckchem) or DMSO (Sigma) was added to the media. After 5 days of culture, cells were harvested and washed with FACS buffer (PBS + 3% FBS). Cells from each condition were split into two dilutions: either containing 20% or 80% of cells. These two dilutions were placed into H4034 Methocult medium (Stemcell Technologies) supplemented with 10ng/mL Flt3-L (PeproTech), 10ng/mL IL-6 (PeproTech) and 10U/mL Penicillin-Streptomycin (Thermo Fisher Scientific) and split into duplicate wells (2 x 10% cell suspension and 2 x 40% cell suspension) of a 6 well plate. After 14 days the number of colonies in each condition was counted using the Stemvision analyser (Stemcell Technologies) (primary plating analysis).

After counting, the duplicate well contents were harvested and washed with FACS buffer (3% FBS in PBS). The harvested cells were again split into two dilutions (either containing 20% or 80% of cells), and placed into H4034 Methocult (Stemcell Technologies) with added cytokines and Penicillin-Streptomycin, and were then split into duplicate wells. After a further 14 days the colonies were imaged and counted (secondary plating analysis). In the text we refer to the initial sorted population as ‘HSCs’, but as the immunophenotype of their progeny is not determined after 7-day cultures before replating assays, and as cell types with colony forming capacity are retained, we refer to the *ex vivo* cultured HSCs as HSPCs.

Supplemental Figures

Supplemental Figure 1: STAT5 loss results in defective HSC function

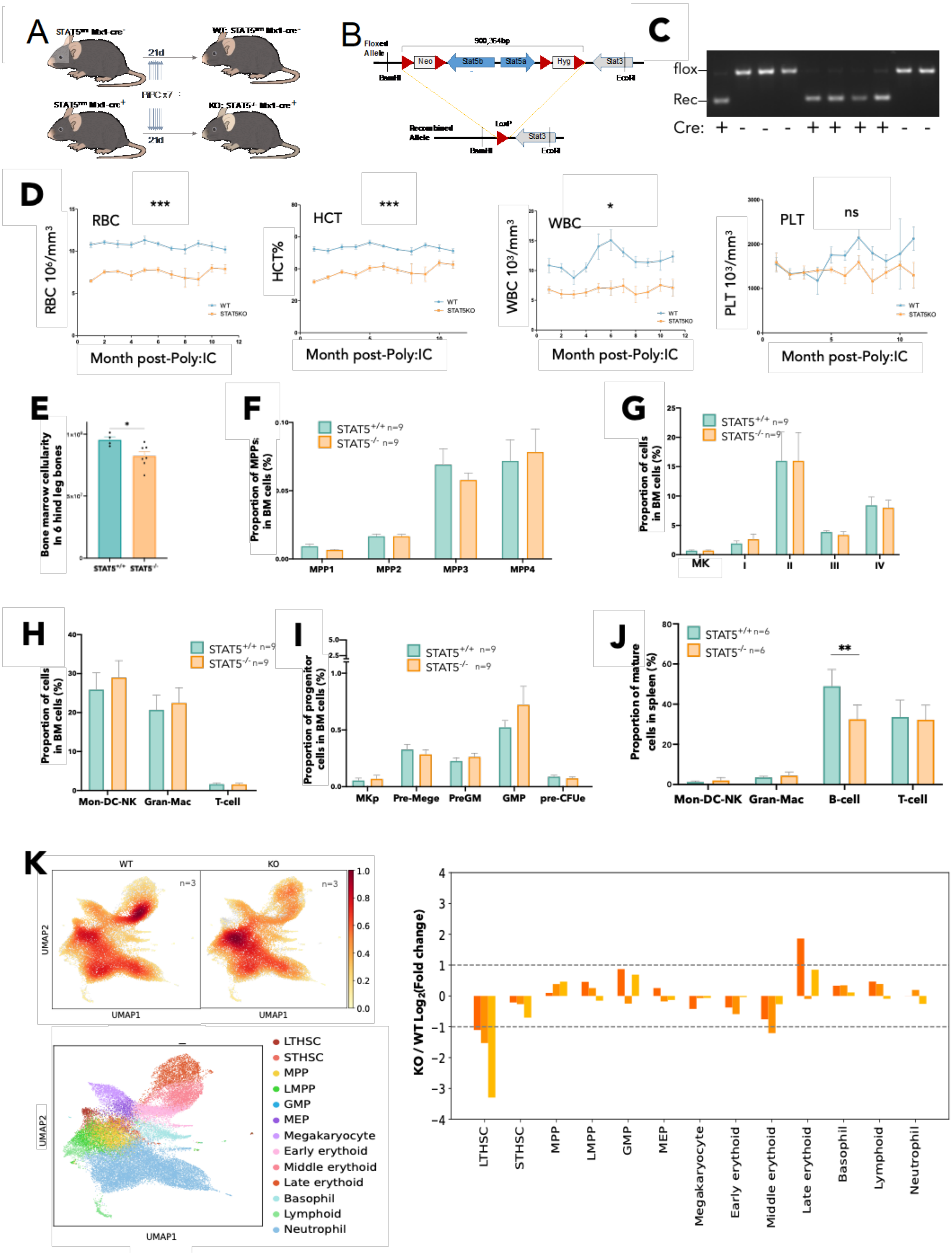

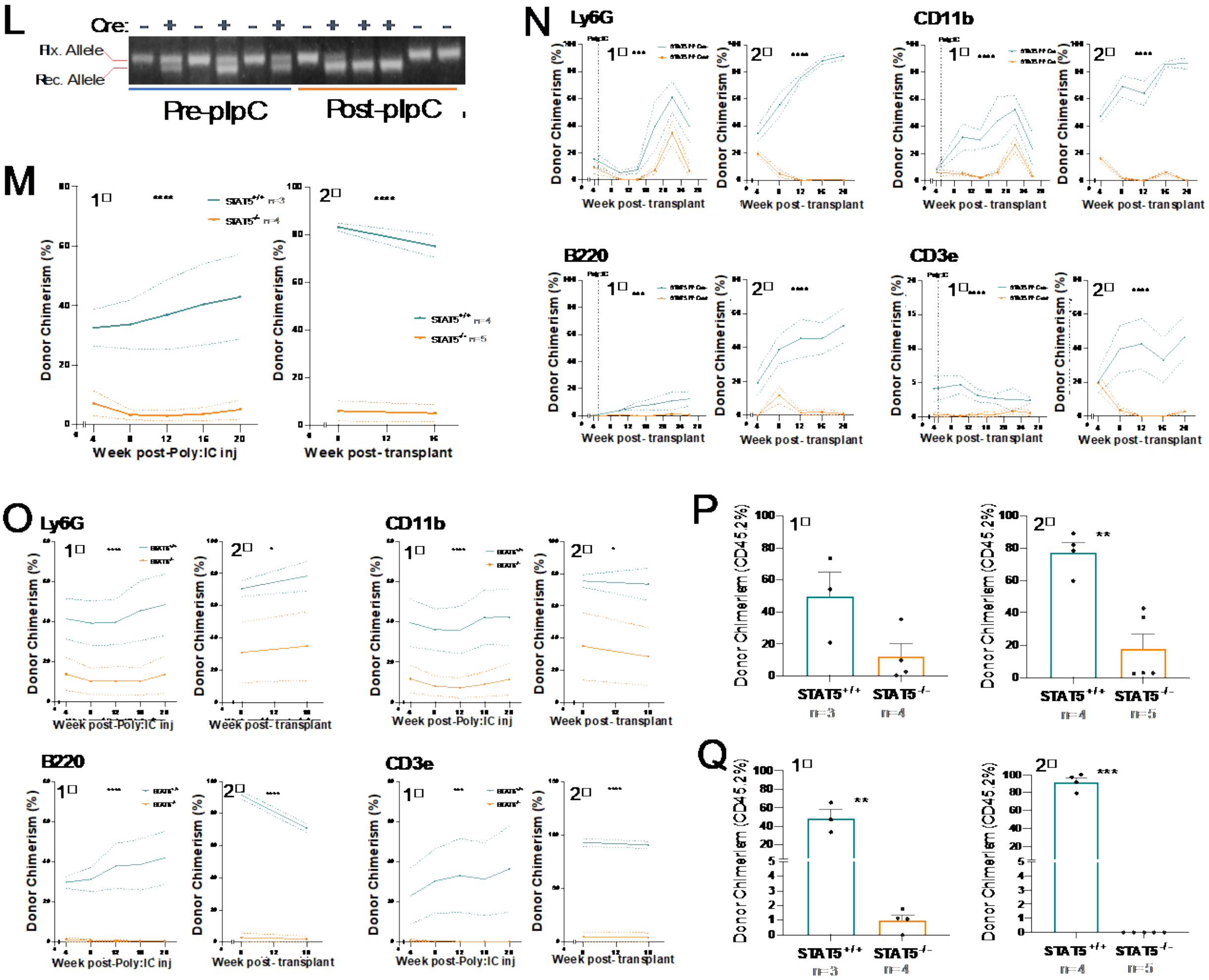

Supplemental Figure 1: STAT5 loss results in defective HSC function

(A) Schematic diagram showing induction of STAT5 deletion by repeated Poly:IC treatments. Both STAT5$^{f/f}$Cre^{+} and STAT5$^{f/f}$Cre^{-} mice were treated with Poly:IC over 21 days to induce deletion of *Stat5* locus in Mx1Cre^{+} mice. Further analysis was conducted at least 4 weeks post final injection. (B) Diagram depicting floxed allele with LoxP sites indicated and the recombined allele after Cre mediated deletion of the *Stat5* locus. (C) PCR gel-electrophoresis picture showing recombined band only present in Cre^{+} mice. Comparison of PCR band intensity for floxed and recombined alleles was used to estimate recombination rate. (D) line graph showing RBC, HCT, WBC and PLT counts in peripheral blood from WT and STAT5-deficient mice. Asterisks indicate significant differences by ANOVA column

factor (***, P<0.001; *, P<0.001). E) Bar plot showing the number of BMMNCs per 6 hind-leg bones in WT and STAT5-deficient mice (mean ± SEM). (F) Bar plots showing the frequency of MPP1 ($Flk2^{-}CD150^{+}CD48^{-}LSK$), MPP2 ($Flk2^{-}CD150^{+}CD48^{+}LSK$), MPP3 ($Flk2^{-}CD150^{-}CD48^{+}LSK$) and MPP4 ($Flk2^{+}CD150^{-}CD48^{+}LSK$) cells in BMMNCs from WT and STAT5-deficient mice (mean ± SEM). (G) Bar plots showing the frequency of megakaryocyte ($CD41^{+}CD42d^{+}$) and erythroid precursor cells (I, $CD71^{hi}Ter119^{mid}$; II, $CD71^{hi}Ter119^{hi}$; III, $CD71^{mid}Ter119^{hi}$; IV, $CD71^{low}Ter119^{hi}$) in BMMNCs (mean ± SEM). (H) Bar plot showing the frequency of monocytes, dendritic and natural-killer cells ($Ly6G^{+}$), granulocytes and macrophages ($CD11b^{+}$), and T-cells ($CD3e^{+}$) in BMMNCs (mean ± SEM). (I) Bar plots showing the frequency of MKp ($Lin^{-}Sca1^{-}cKit^{+}CD150^{+}CD41^{+}$), pre-Meg/E ($Lin^{-}Sca1^{-}cKit^{+}CD41^{-}CD16/32^{-}CD105^{-}CD150^{+}$), pre-GM ($Lin^{-}Sca1^{-}cKit^{+}CD41^{-}CD16/32^{-}CD105^{-}CD150^{-}$), GMP ($Lin^{-}Sca1^{-}cKit^{+}CD41^{-}CD16/32^{+}CD150^{-}$), and pre-CFUe ($Lin^{-}Sca1^{-}cKit^{+}CD41^{-}CD16/32^{-}CD105^{+}CD150^{+}$) cells in BMMNCs (mean ± SEM). (J) Bar plot showing the frequency of monocytes, dendritic and natural-killer cells granulocytes and macrophages, B cells ($B220^{+}$) and T-cells in spleen mono-nuclear cells (mean ± SEM). Asterisks indicate significant differences by Student's t test (*, P<0.05; **, P<0.01; ***, P<0.001), unless otherwise specified. (K) Left; UMAPs showing the density of cells in WT and STAT5-deficent HSPCs from scRNAseq data of FACS sorted LK ($Lin^{-}c\text{-}Kit^{+}$) cells from WT (n=3) and STAT5-deficient (n=3) BM. Cells were projected onto a combined landscape of over 40,000 HSPCs from Nestorowa *et al.* (2016) and Dahlin *et al.* (2018), and nearest neighbours' analysis was performed to ascribe cell identity and cell type annotation. Cell-type key is shown on bottom left UMAP. Right; bar graph showing the relative abundance of HSPC

cell types. (L) Representative PCR gel showing floxed and recombined bands in peripheral bloods of recipients pre- and post- poly:IC. PCR shows partial recombination of the STAT5 locus occurred in Cre^+ donors in recipients before Poly:IC injections, but near complete recombination is seen after Poly:IC injections. (M) Connected line graphs showing donor chimerism in peripheral blood mono-nuclear cells at each time point in primary and secondary recipients (mean ± SEM). Asterisks indicate significant differences by ANOVA column factor (****, P<0.0001). Results were from an independent replicate competitive transplant experiment (Experiment II). (N) Connected line graphs showing donor chimerism in $Ly6G^+$, $CD11b^+$, $B220^+$ and $CD3e^+$ peripheral blood cells in primary and secondary recipients (mean ± SEM). Asterisks indicate significant differences by ANOVA column factor (****, P<0.0001; ***, P<0.001). Results from experiment I. (O) Connected line graphs showing donor chimerism in $Ly6G^+$, $CD11b^+$, $B220^+$ and $CD3e^+$ peripheral blood cells in primary and secondary recipients (mean ± SEM). Asterisks indicate significant differences by ANOVA column factor (****, P<0.0001; *, P<0.05). Results from experiment II. (P) Bar plots showing total BMMNC donor chimerism in primary and secondary recipients (mean ± SEM) from experiment II. (Q) Bar plots showing LT-HSC donor chimerism in primary and secondary recipients (mean ± SEM) from experiment II. Asterisks indicate significant differences by Student's t test (***, p<0.001; **, p<0.01).

Supplemental Figure 2: **STAT5-deficient HSCs display reduced cell cycle entry, increased differentiation, and reduced retention of lineage-negative progeny.**

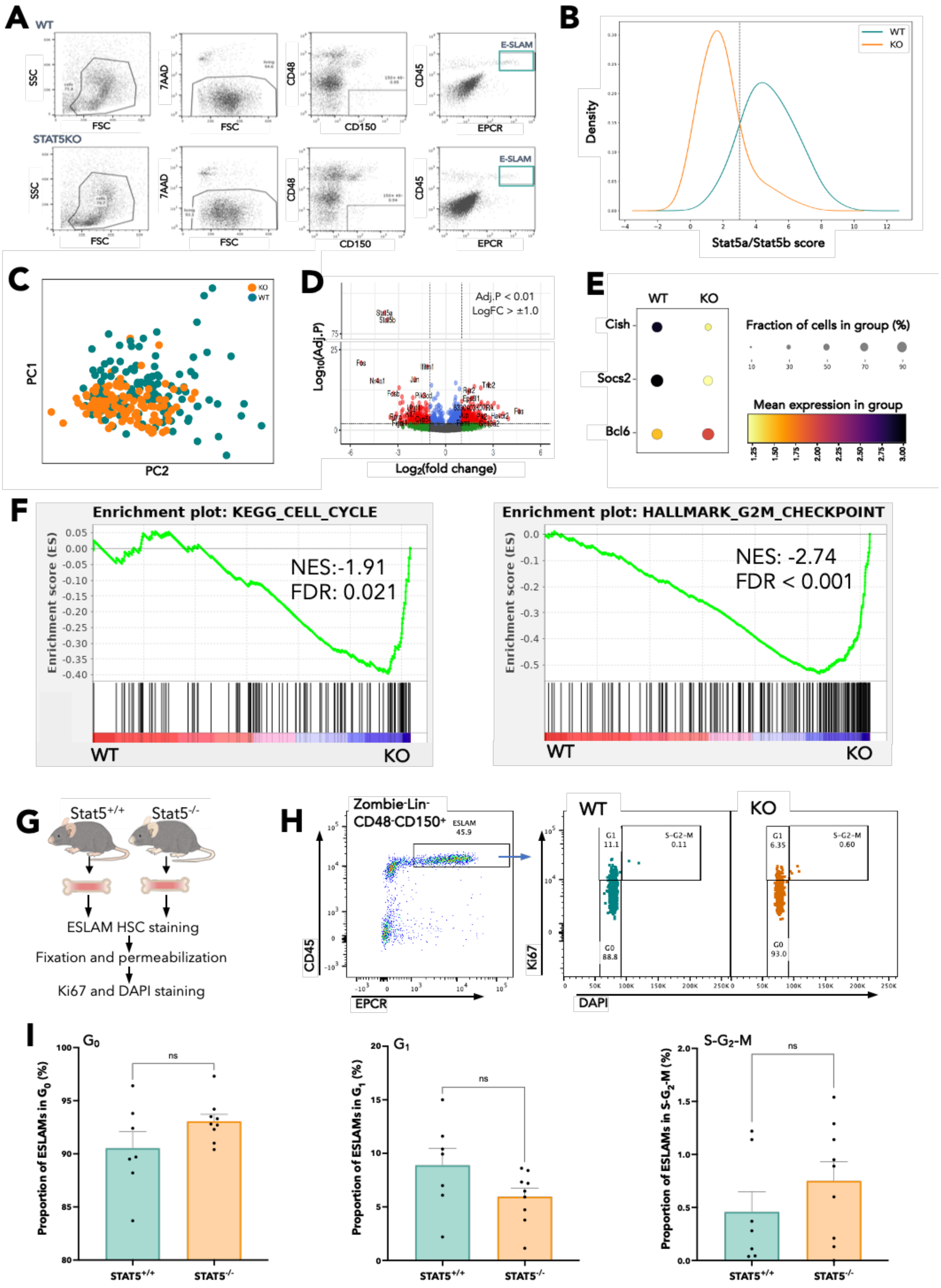

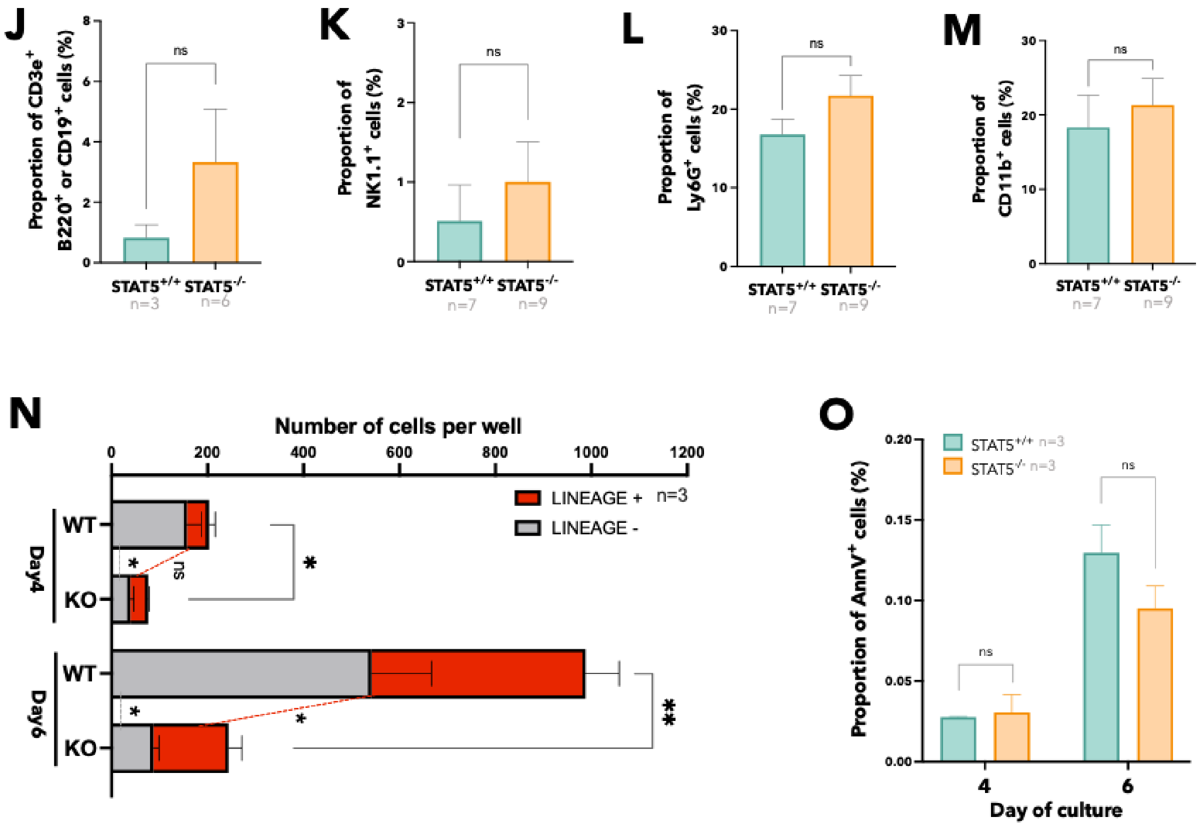

Supplemental Figure 2: ***STAT5-deficient HSCs display reduced cell cycle entry, increased differentiation, and reduced retention of lineage-negative progeny.***

(A) Gating strategy used to isolate ESLAM HSCs from STAT5$^{-/-}$ and WT mice for ScRNAseq. Bone marrow cells were collected from 6 hind-leg bones and lineage depleted before antibody staining for ESLAM markers (CD45^{+}CD48^{-}CD150^{+}ECPR^{+}) and FACS sorting. (B) Line graph showing the kernel density estimates of HSCs from STAT5 deficient mice and WT mice showing the geometric mean score of *Stat5a* and *Stat5b* expression. The dotted vertical line indicates where HSCs from WT and STAT5 deficient mice overlap in Stat5a/b transcripts. All HSCs from STAT5 deficient mice above the dotted line were removed before further analysis (n=23 HSCs removed). (C) PCA analysis showing the location of STAT5$^{-/-}$ and WT HSCs based on principal component analysis. (D) Volcano plot showing differentially expressed genes (red dots) in STAT5$^{-/-}$ HSCs with a Log$_2$

Fold Change >1.0 and Benjamini-Hochberg corrected p-value <0.01. (E) Dot plot showing differentially expressed canonical STAT5 target genes. (F) Gene set enrichment analysis (GSEA) plots showing depleted cell cycle related signatures in STAT5-deficient ESLAM HSCs. Normalised enrichment scores (NES) and false discovery rate (FDR) are indicated. (G) Schematic diagram showing cell cycle analysis of HSCs using intracellular flow cytometry after staining with Ki-67 and DAPI. BM from $STAT5^{-/-}$ and WT mice was lineage depleted, stained with ESLAM HSCs panel. Cells were then fixed and permeabilised and stained for Ki67 and DAPI for cell cycle analysis. (H) Example of gating of fixed and permeabilised BM lineage negative cells for ESLAM HSC cell cycle analysis with different stages of cell cycle indicated. (I) Bar plot showing the proportion of ESLAM HSCs in G_0, G_1 and S-G_2-M phases of the cell cycle based on Ki-67 and DAPI staining. Each dot represents the mean of ESLAMs in an indicated phase from one WT or STAT5-deficient mouse. (J) Bar plot showing the frequency of lymphocyte lineage marker ($CD3e^+$/$CD19^+$/$B220^+$) expressing cells at day 5 in *ex vivo* IL-11/SCF culture (mean ± SEM). (K) Bar plot showing the frequency of natural killer lineage marker ($NK1.1^+$) expressing cells (mean ± SEM) produced by WT or STAT5-defcient HSCs at day 5 in *ex vivo* IL-11/SCF culture. (L) Bar plot showing the frequency of monocytes and neutrophils lineage marker ($Ly6G^+$) expressing cells (mean ± SEM) produced by WT or STAT5-defcient HSCs at day 5 in *ex vivo* IL-11/SCF culture. (M) Bar plot showing the frequency of granulocytes and macrophages lineage marker ($CD11b^+$) expressing cells (mean ± SEM) produced by WT or STAT5-defcient HSCs at day 5 in *ex vivo* IL-11/SCF culture. (N) Bar plots showing the number of cells per well at day 4 and 6 produced by 50 WT or STAT5-deficient ESLAM HSCs in *ex vivo* IL-11/SCF culture. The number of cells expressing lineage

markers ($Ter119^+$ $Ly6g^+$ $CD11b^+$ $B220^+$ or $CD3e^+$) are in red; the number of lineage negative cells are in grey (mean ± SEM). (O) Bar plot showing the proportion of AnnexinV+ cells in cultures at days 4 and 6 in *ex vivo* IL-11/SCF culture (mean ± SEM). Asterisks indicate significant differences by Student's t test (*, $p<0.05$; **, $p<0.01$).

Supplemental Figure 3: **Unphosphorylated STAT5 constrains HSC differentiation and upregulates transcriptional programs associated with HSC maintenance.**

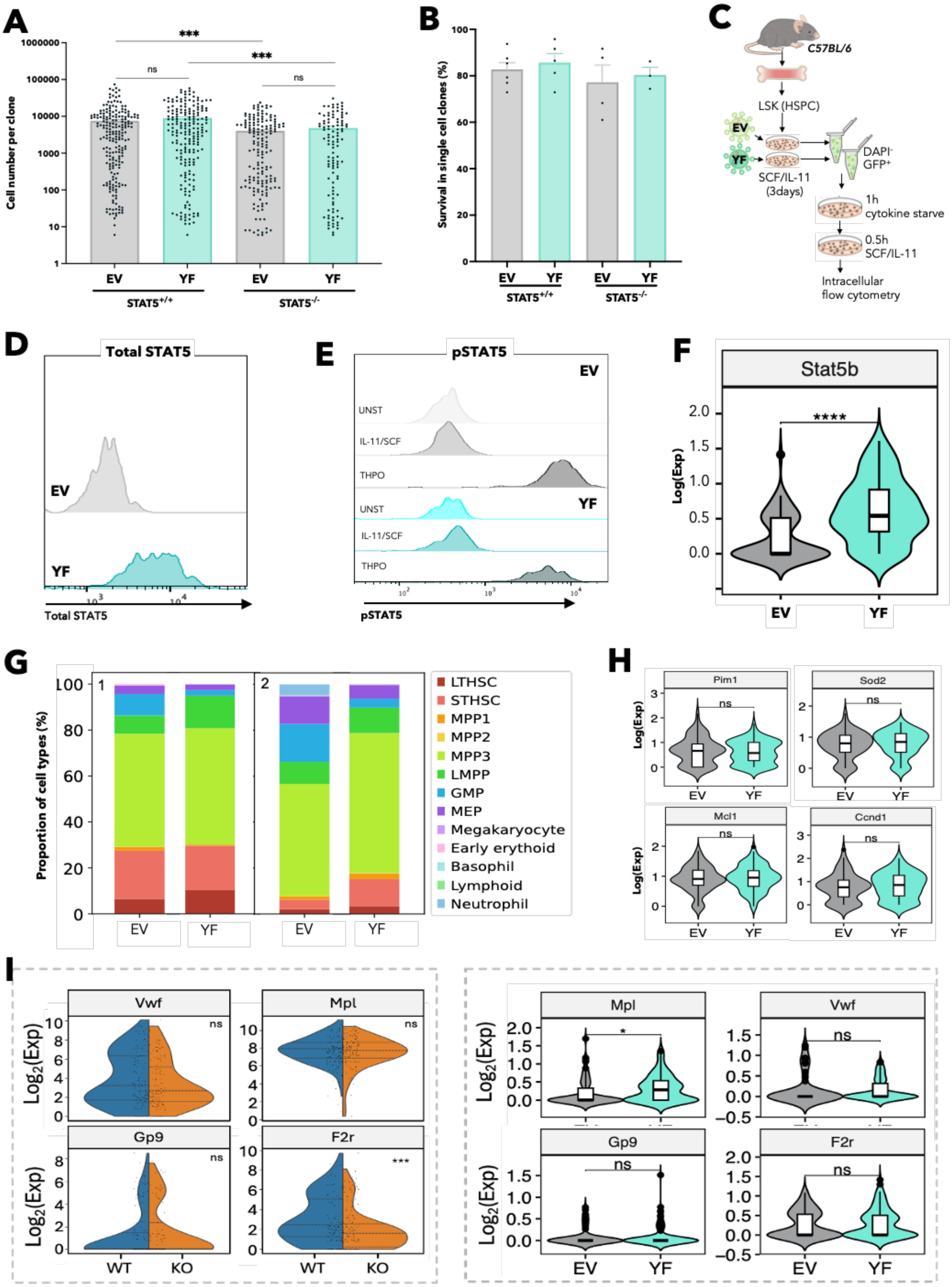

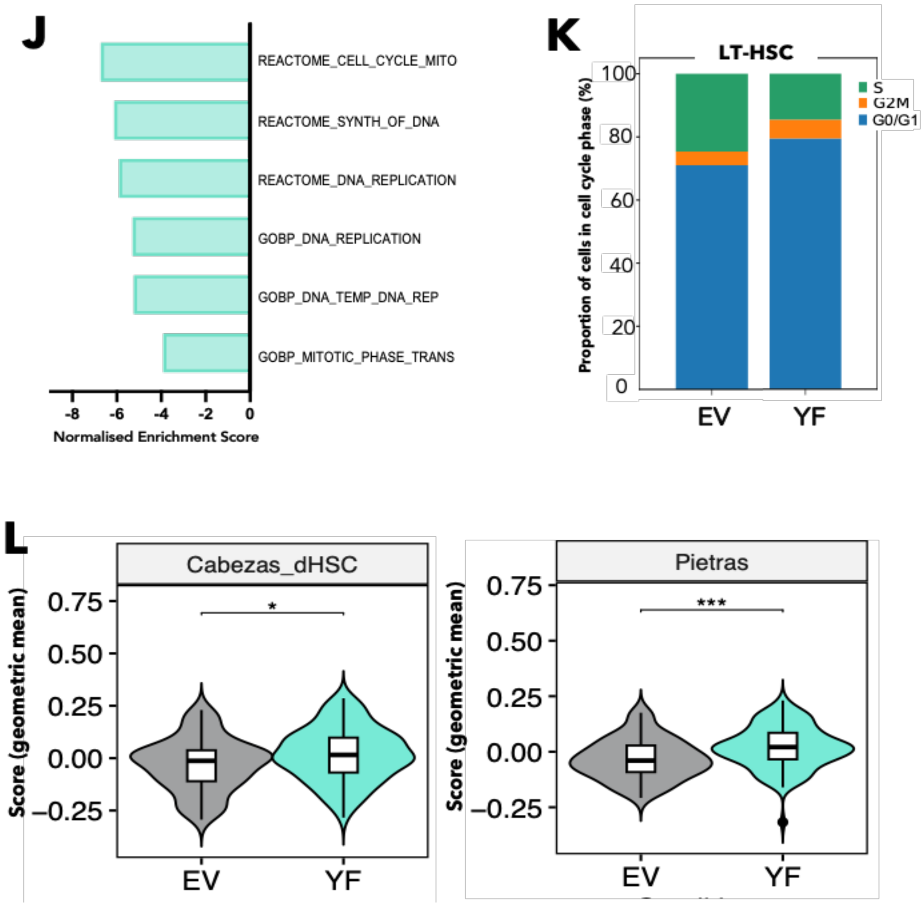

Supplemental Figure 3: **Unphosphorylated STAT5 constrains HSC differentiation and upregulates transcriptional programs associated with HSC maintenance.**

(A) Bar plot showing the number of cells per well at day 7 after single cells were sorted per well into SCF/IL-11 maintenance conditions.[2] Each dot represents a single clone and bars represent mean cell number (± SEM); results were from 4 independent experiments. (B) Bar plots showing clone survival rate after 5 days in culture. Clone survival rate was shown as the proportion of wells that contained cells at day 5. Each dot represents the frequency of surviving clones from each biological replicate; bars show the mean ± SEM survival from all experiments. (C) Schematic diagram showing intracellular flow cytometry analysis of pSTAT5 and total STAT5 protein levels. Lineage$^-$Sca1$^+$cKit$^+$ (LSK) were FACS sorted from WT bone marrow and transduced with lentivirus containing STAT5B-Y699F(YF), or EV

in SCF/IL-11 maintenance cultures.[2] After three days, GFP^{+} living cells were sorted and starved in cytokine free media for 1h, followed by a 30-minute stimulation with IL-11/SCF maintenance cultures. After stimulation cells were fixed and permeabilized and stained with anti-STAT5 antibody or anti-pSTAT5 antibody. (D) Example of histograms for total-STAT5 antibody staining of LSK cells transduced with STAT5B-YF or EV lentivirus. GFP+ living cells were sorted 3-days post transduction and starved in cytokine free media for 1h before being placed into either starved media or, IL-11/SCF culture, a media with cytokine cocktail containing high doses of THPO (100ng/ml) was included for positive control. (E) Example of histograms for pSTAT5 antibody staining of LSK cells transduced with STAT5B-YF or EV lentivirus. GFP+ living cells were sorted 3-days post transduction and starved in cytokine free media for 1h before being placed into either starved media or, IL-11/SCF culture, or media containing a cytokine rich cocktail containing high doses of THPO (100ng/ml) was included for positive control. (F) Violin plot showing the normalised expression of *Stat5b*. Asterisks indicate significant differences by Benjamini-Hochberg corrected p-value (****, adj. p<0.001). (G) Bar plots showing the frequency of annotated cell types in each independent experiment after datasets were projected onto a combined landscape of over 40,000 HSPCs from Nestorowa *et al.* (2016) and Dahlin *et al.* (2018). After projection, nearest neighbours analysis was performed to annotate cell types within datasets. (H) Violin plots showing the expression level and frequency of cells expressing canonical pSTAT5 target genes; *Pim1,*[16] *Ccnd1,*[17] *Mcl1*,[18] and *Sod2*[19,20] in STAT5B-YF or EV infected LT-HSCs. (I) Violin plots showing the expression level and frequency of cells expressing uSTAT5 target megakaryocytic genes; *Mpl, Vwf, Gp9* and *F2r* in STAT5$^{-/-}$ and STAT5$^{+/+}$ ESLAM HSCs (left) and STAT5B-YF or EV

infected LT-HSCs (right). (J) Horizontal bar plots showing the normalized enrichment scores (NES) for significantly depleted (false discovery rate <0.001) cell cycle genes sets in STAT5-YF infected LT-HSCs compared to EV LT-HSCs. (K) Stacked bar plot showing the frequency of transcriptionally defined, EV or STAT5-YF-infected LT-HSCs in indicated cell cycle phases (G_0/G_1 phases, reflecting cells that scored below 0 for S-phase or G_2M cell cycle scores; S or G_2M phases) based on transcriptional cell cycle scores. (L) Violin plots showing the geometric mean score of STAT5B-YF or EV infected LT-HSCs for two published HSC signatures: dHSC (dormant HSCs), which was derived from comparing dormant to active HSCs from Cabezas-Wallscheid *et al.* (2017)[21] as dormant HSCs retain greatest HSC repopulating capacity; and a primitive HSC score comparing Flt3$^-$SLAM LT-HSCs with multipotent progenitors from Pietras *et al.* (2014).[22] Asterisks indicate significant differences by Student's t test (*, $p<0.05$; ***, $p<0.001$; ****, $p<0.0001$).

Supplemental Figure 4: **Unphosphorylated STAT5 enhances HSPC clonogenicity *in vitro* and HSC maintenance *in vivo*.**

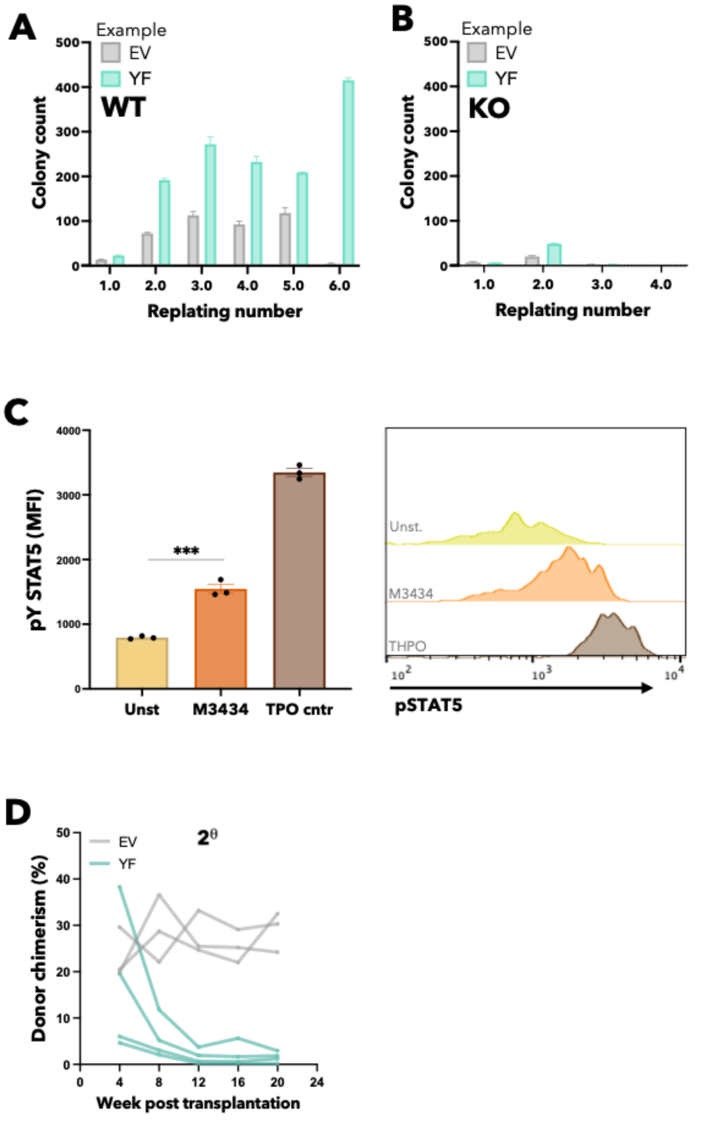

Supplemental Figure 4: **Unphosphorylated STAT5 enhances HSPC clonogenicity *in vitro* and HSC maintenance *in vivo*.**

(A) Bar graph showing an example of the number of colonies at each replating week for one biological replicate of WT HSCs transduced with STAT5B-YF, or EV (n=2 technical replicates). (B) Bar graph showing number of colonies at each replating week for STAT5-deficient HSCs transduced with STAT5B-YF, or EV (n=2 technical replicates). Data was collected at the same time as A. (C) Left; Bar plots showing the mean fluorescent intensity of pSTAT5 antibody

staining by intracellular flow-cytometry analysis in ESLAM HSCs unstimulated, semi-solid methylcellulose condition used in replating assays (M3434), and THPO (200ng/ml) positive control conditions (mean ± SEM). Right; example of histograms showing mean fluorescent intensity of pSTAT5 staining of ESLAM HSCs stimulated with indicated culture conditions. Each bar plot shows the mean ±SEM. (D) Line graph showing the peripheral blood donor chimerism of HSCs transduced with EV (n=3), or STAT5B-Y699F (n=4) for each recipient mouse in secondary competitive transplants. Asterisks indicate significant differences by Student's t test (***, $p<0.001$).

Supplemental Figure 5 **Ruxolitinib enhances HSPC clonogenicity and maintains transplantable HSCs.**

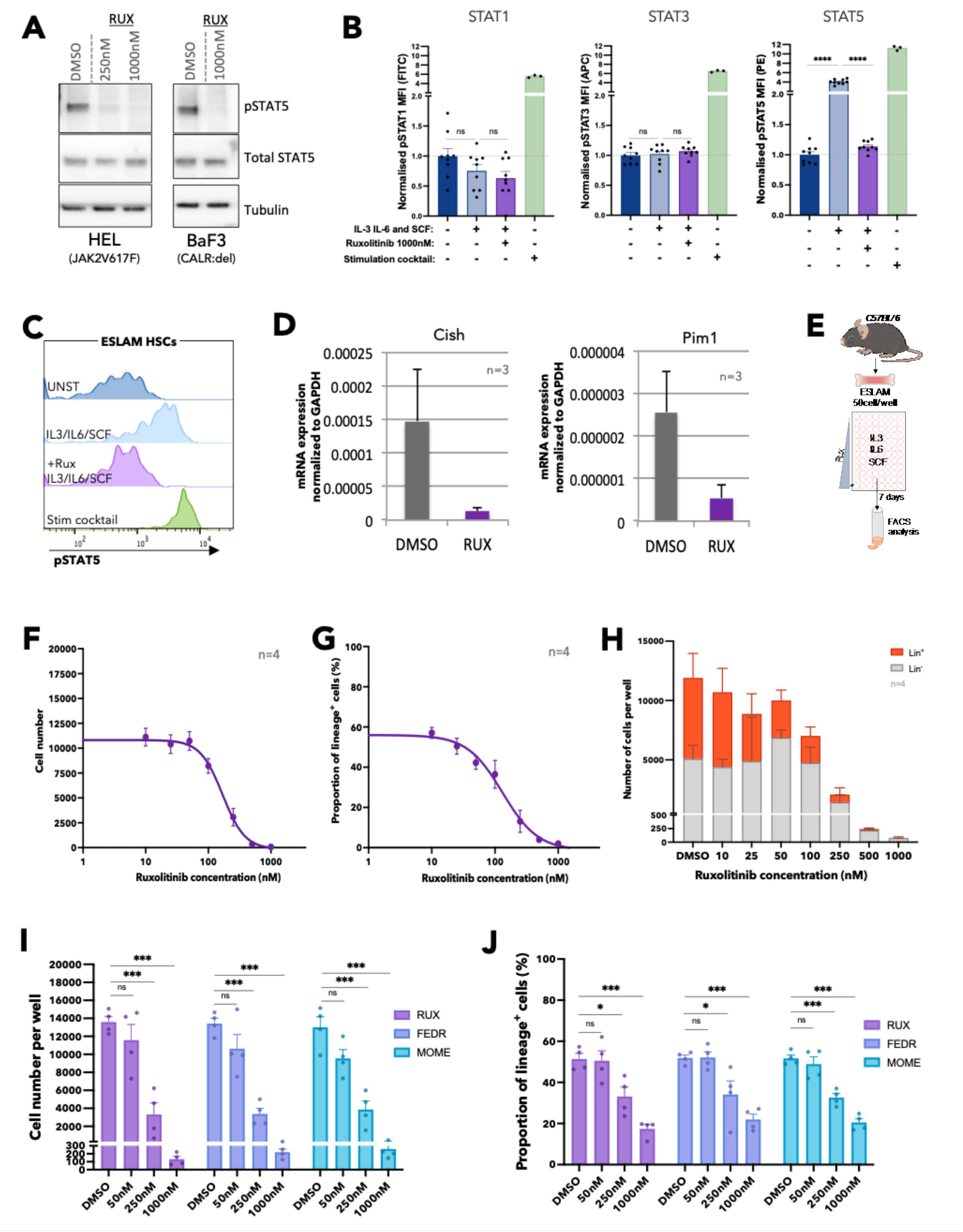

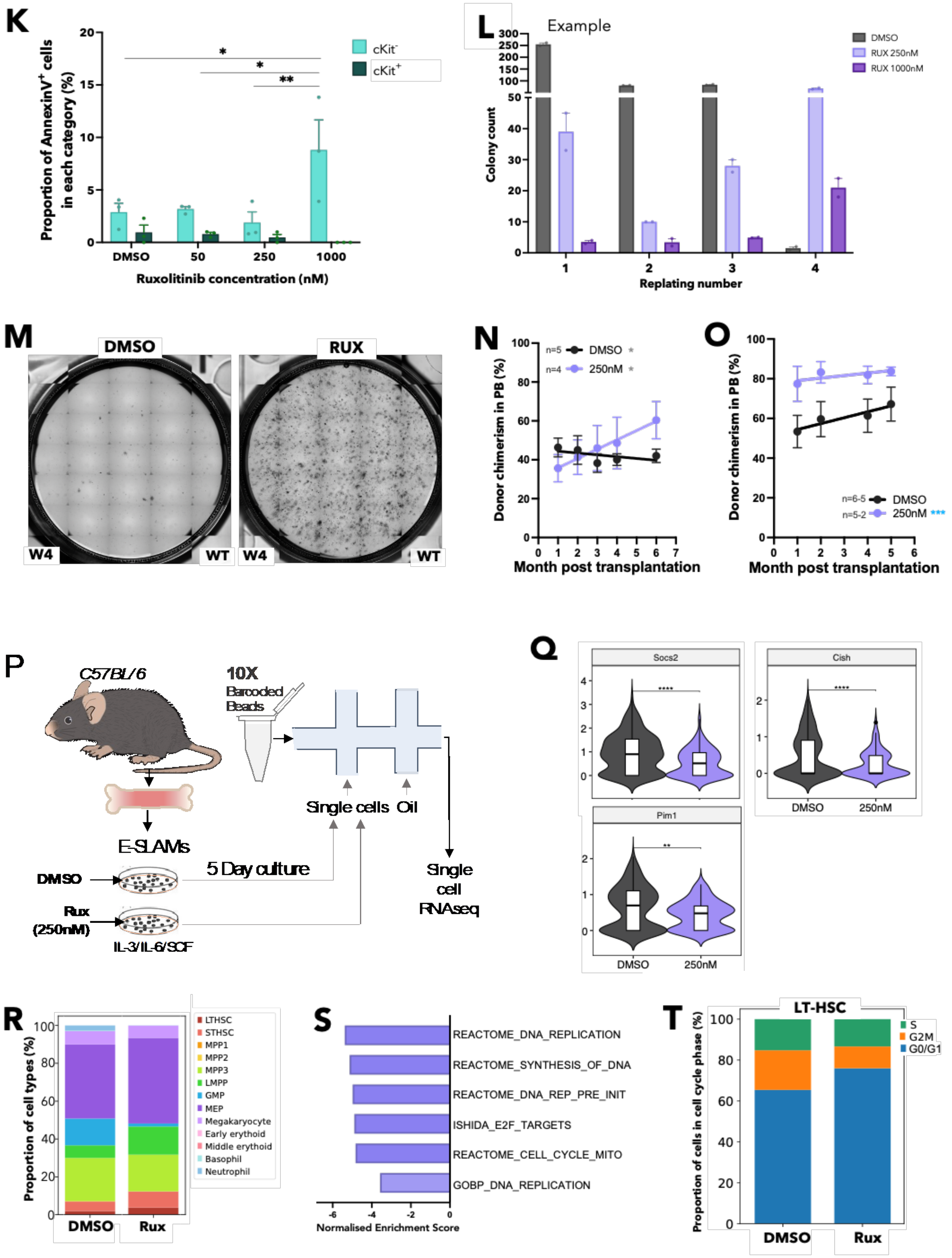
K
Proportion of AnnexinV+ cells in each category (%)
cKit-
cKit+
Ruxolitinib concentration (nM)
DMSO
50
250
1000
L
Example
DMSO
RUX 250nM
RUX 1000nM
Colony count
Replating number
M
DMSO
RUX
W4
WT
N
n=5 DMSO
n=4 250nM
Donor chimerism in PB (%)
Month post transplantation
O
n=6-5 DMSO
n=5-2 250nM
Donor chimerism in PB (%)
Month post transplantation
P
C57BL/6
10X
Barcoded
Beads
Single cells
Oil
E-SLAMs
5 Day culture
DMSO
Rux
(250nM)
IL-3/IL-6/SCF
Single
cell
RNAseq
Q
Socs2
Cish
Pim1
DMSO
250nM
R
Proportion of cell types (%)
LTHSC
STHSC
MPP1
MPP2
MPP3
LMPP
GMP
MEP
Megakaryocyte
Early erythoid
Middle erythroid
Basophil
Neutrophil
DMSO
Rux
S
REACTOME_DNA_REPLICATION
REACTOME_SYNTHESIS_OF_DNA
REACTOME_DNA_REP_PRE_INIT
ISHIDA_E2F_TARGETS
REACTOME_CELL_CYCLE_MITO
GOBP_DNA_REPLICATION
Normalised Enrichment Score
T
LT-HSC
S
G2M
G0/G1
Proportion of cells in cell cycle phase (%)
DMSO
Rux

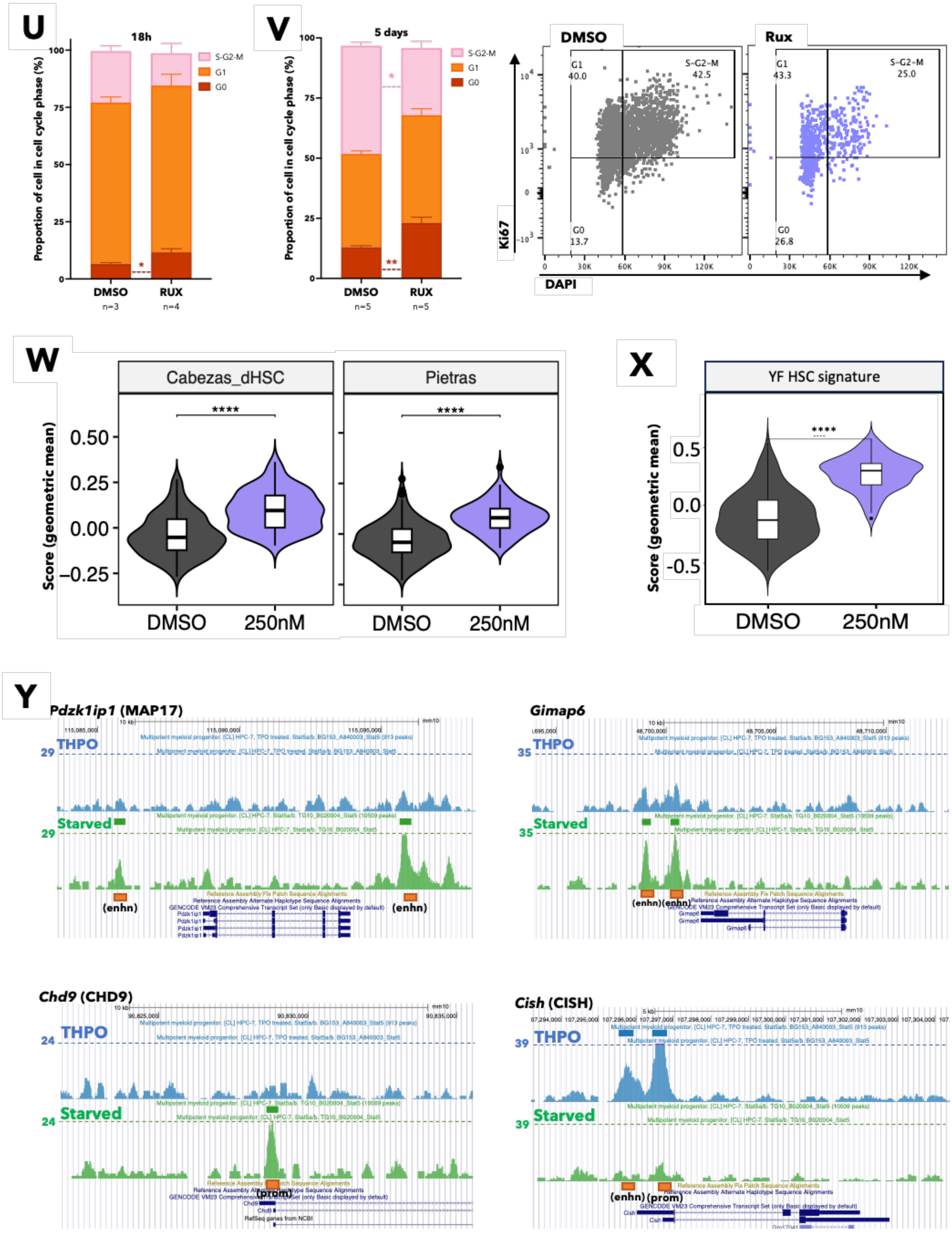
U
18h
Proportion of cell in cell cycle phase (%)
S-G2-M
G1
G0
DMSO
n=3
RUX
n=4
V
5 days
DMSO
n=5
RUX
n=5
DMSO
Rux
G1
40.0
S-G2-M
42.5
G0
13.7
G1
43.3
S-G2-M
25.0
G0
26.8
Ki67
DAPI
W
Cabezas_dHSC
Pietras
Score (geometric mean)
DMSO
250nM
X
YF HSC signature
Y
Pdzk1ip1 (MAP17)
Gimap6
Chd9 (CHD9)
Cish (CISH)
THPO
Starved
(enhn)
(prom)

***Supplemental Figure 5:* Ruxolitinib enhances HSPC clonogenicity and maintains transplantable HSCs.**

(A) Western blot showing the staining of anti-pSTAT5A/B and anti-total STAT5 antibodies with and without ruxolitinib in HEL cells and Ba/F3 cells expressing both MPL and mutant CALR (52bp deletion, del). (B) Bar plots showing the mean fluorescent intensity of staining of anti-pSTAT1, anti-pSTAT3, and anti-pSTAT5 antibodies by intracellular-flow cytometry in ESLAM HSCs cultured in cytokine free conditions or in cultures stimulated with IL-3/IL-6/SCF[3] cultures ± ruxolitinib, or a media with stimulation cocktail (THPO, Flt3-L and IFN-α). Fluorescent pSTAT staining was normalized to the unstimulated condition for each graph (mean ± SEM). Each dot represents the mean fluorescent intensity of ESLAMs from a single mouse. (C) Example of histograms of pSTAT5 fluorescent staining of ESLAM HSCs cultured in the given culture settings. (D) Bar plots showing the normalised mRNA expression of canonical STAT5 target genes in ESLAM HSCs after 2hrs of exposure to vehicle or ruxolitinib in the presence of SCF/IL-3/IL-6[3] (mean ±SEM). (E) Schematic diagram showing *in vitro* assays of WT HSCs treated with Rux. 50 WT ESLAM HSCs were sorted per well into complete SCF/IL-3/IL-6 media[3] with scaled doses of ruxolitinib or DMSO vehicle and were analysed by flow-cytometry 7 days later. (F) Inhibitor response curves showing the number of cells per well at each dose of ruxolitinib after 7 days in culture (mean ± SEM). Experiment described in 'E'. (G) Inhibitor-response curves showing the proportion of cells per well that express mature lineage markers (Ter119$^+$/Ly6g$^+$/CD11b$^+$/B220$^+$/CD3e$^+$) after 7 days in culture at different ruxolitinib concentrations (mean ± SEM). Experiment described in 'E'. (H) Bar plot showing the number of cells that express lineage markers (Lin$^+$) and number of lineage-marker negative (Lin$^-$) cells per well

at day 7 (mean ± SEM). (I) Bar plots showing the cell number per well of HSC derived cultures after 7 days in IL-3/IL-6/SCF cultures in the presence of varying doses of ruxolitinib, Fedratinib or Momelotinib, starting from 50 ESLAM HSCs/well (mean ± SEM). (J) Bar plots showing the proportion (%) of lineage positive cells ($Ter119^+$/$Ly6g^+$/$CD11b^+$/$B220^+$/ $CD3e^+$) per well of HSC derived cultures after 7 days in IL-3/IL-6/SCF cultures in the presence of varying doses of ruxolitinib, Fedratinib or Momelotinib, starting from 50 ESLAM HSCs/well (mean ± SEM). (K) Bar plot showing the proportion of apoptotic $cKit^+$ or $cKit^-$ cells (Annexin V^+) in cultures derived from lineage depleted BM after 16h with ruxolitinib or vehicle (mean ± SEM). (L) Bar graph showing an example of colony numbers recorded at each replating week for WT HSCs from 1 mouse, treated for 7-days with vehicle or ruxolitinib at 250nM or 1000nM (n=2 technical replicates). (M) Example images of colonies at the final week (4th week) of serial colony plating for WT HSCs treated for 7-days with vehicle or ruxolitinib at 250nM. (N) Scatter dot plot with linear regression line of best fit showing the peripheral blood donor chimerism in primary competitive transplants of 5-day *ex vivo* cultured HSCs with ruxolitinib or vehicle. 50 ESLAMs from WT mice were seeded per well in IL-3/IL-6/SCF culture conditions and given DMSO or 250nM of ruxolitinib for 5 days before the wells were harvested and pooled for each condition and an equivalent of 50 starting ESLAMs was transplanted per recipient with $3x10^5$ competitor bone marrow cells. Each dot indicates mean donor chimerism (mean ± SEM). Grey asterisks indicate significant differences in the slopes of linear regressions modelling ruxolitinib treated donor cell chimerism compared to DMSO treated donor cell chimerism in transplant (*, $p<0.05$). (O) Scatter dot plot with linear regression line of best fit showing the peripheral blood donor chimerism in secondary transplants of 5-day *ex vivo*

cultured HSCs with ruxolitinib or vehicle. Each dot indicates mean donor chimerism (mean ± SEM). Blue asterisks indicate significant differences in y-intercepts of linear regressions modelling ruxolitinib treated donor cell chimerism compared to DMSO treated donor cell chimerism in transplants (***, p<0.001). (P) Schematic diagram showing experimental outline for scRNAseq of WT ESLAM HSCs treated with RUX or DMSO. WT ESLAM HSCs were FACS sorted and cultured in IL-3/IL-6/SCF complete media[3] with vehicle or 250nM of ruxolitinib and were allowed to expand for 5 days. Living cells were then sorted for 10X Genomics ScRNAseq. (Q) Violin plots showing the mean expression and frequency of LT-HSCs expressing canonical pSTAT5 target genes; Cish, Pim1, and Socs2. LTHSCs were transcriptionally defined within scRNAseq dataset of the day-5 HSC derived cultures treated with DMSO or ruxolitinib. (R) Bar plots showing the frequency of annotated cell types after datasets were projected onto a combined landscape from Nestorowa *et al.* (2015) and Dahlin *et al.* (2018). Nearest neighbours analysis was performed after projection to assign cell types. (S) Horizontal bar plot showing the normalised enrichment scores of significantly depleted (false discovery rate <0.001) cell cycle gene sets in ruxolitinib treated LT-HSCs compared to DMSO treated LT-HSCs. Results from gene set enrichment analysis (GSEA). (T) Stacked bar plot showing the frequency of transcriptionally defined, DMSO or ruxolitinib-treated LT-HSCs in indicated cell cycle phases (G_0/G_1 phases, reflecting cells that scored below 0 for S-phase or G_2M-phase cell cycle scores) based on transcriptional cell cycle scores. (U) Bar plots showing the cell cycle phase frequency of ESLAM HSCs treated with DMSO (n=3) or ruxolitinib (n=4) after 18h in IL-3/IL-6/SCF[3] cultures. Cell cycle status was derived from on Ki67/DAPI staining. (V) Left; bar plots showing the cell cycle phase frequency of ESLAM HSC

derived cultures treated with DMSO (n=5) or ruxolitinib (n=5) after 5 days in IL-3/IL-6/SCF[3] cultures. Cell cycle status was derived from on Ki67/DAPI staining (Right). G_0 represents quiescent cells that are $Ki67^{low}DAPI^{low}$; G_1 represents cells in early growth phase, which are $Ki67^{high}DAPI^{low}$; S-G2-M represents cells in DNA synthesis, late growth, and mitosis stages of active cell cycling and are $Ki67^{high}DAPI^{high}$. (W) Violin plots showing the distribution of geometric mean scores (mean of transcript counts across genes within a given dataset) of DMSO or Ruxolitinib treated LT-HSCs for two previously published HSC signatures: dHSC (dormant HSCs), which was derived from comparing dormant to active HSCs from Cabezas-Wallscheid *et al.* (2017)[21] as dormant HSCs retain greatest HSC repopulating capacity; and a primitive HSC score comparing $Flt3^{-}$SLAM LT-HSCs with multipotent progenitors from Pietras *et al.* (2014).[22] (X) Violin plots showing the geometric mean scores (mean of transcript counts across genes within dataset) of DMSO or Ruxolitinib treated LT-HSCs against the signature of STAT5-YF LT-HSCs from Figure 3. This signature was derived by taking the top 100 significantly upregulated genes (FDR <0.01) in STAT5-YF expressing LT-HSCs compared to control EV transduced LT-HSCs from Figure 3. (Y) Representative histogram plots showing the presence of STAT5 binding near example genes known to promote HSC maintenance; these include Pdzk1ip1 (MAP17), Chd9, and Gimap6 in HPC7 cells in cytokine starved condition (i.e. uSTAT5 peak in green), whereas no STAT5 binding is detected in cells cultured with 100ng/ml of THPO (pSTAT5 peak in blue).[23] Cish is provided as an example of a pSTAT5 target gene, i.e. STAT5 binding is detected in THPO stimulated conditions, and is absent in cytokine starved conditions. Asterisks indicate significant differences by Student's t test (****, p<0.0001; ***, p<0.001; **, p<0.01; *, p<0.05) unless otherwise indicated.

Supplementary Figure 6: Ruxolitinib maintains murine and human myeloproliferative neoplasm HSPCs.

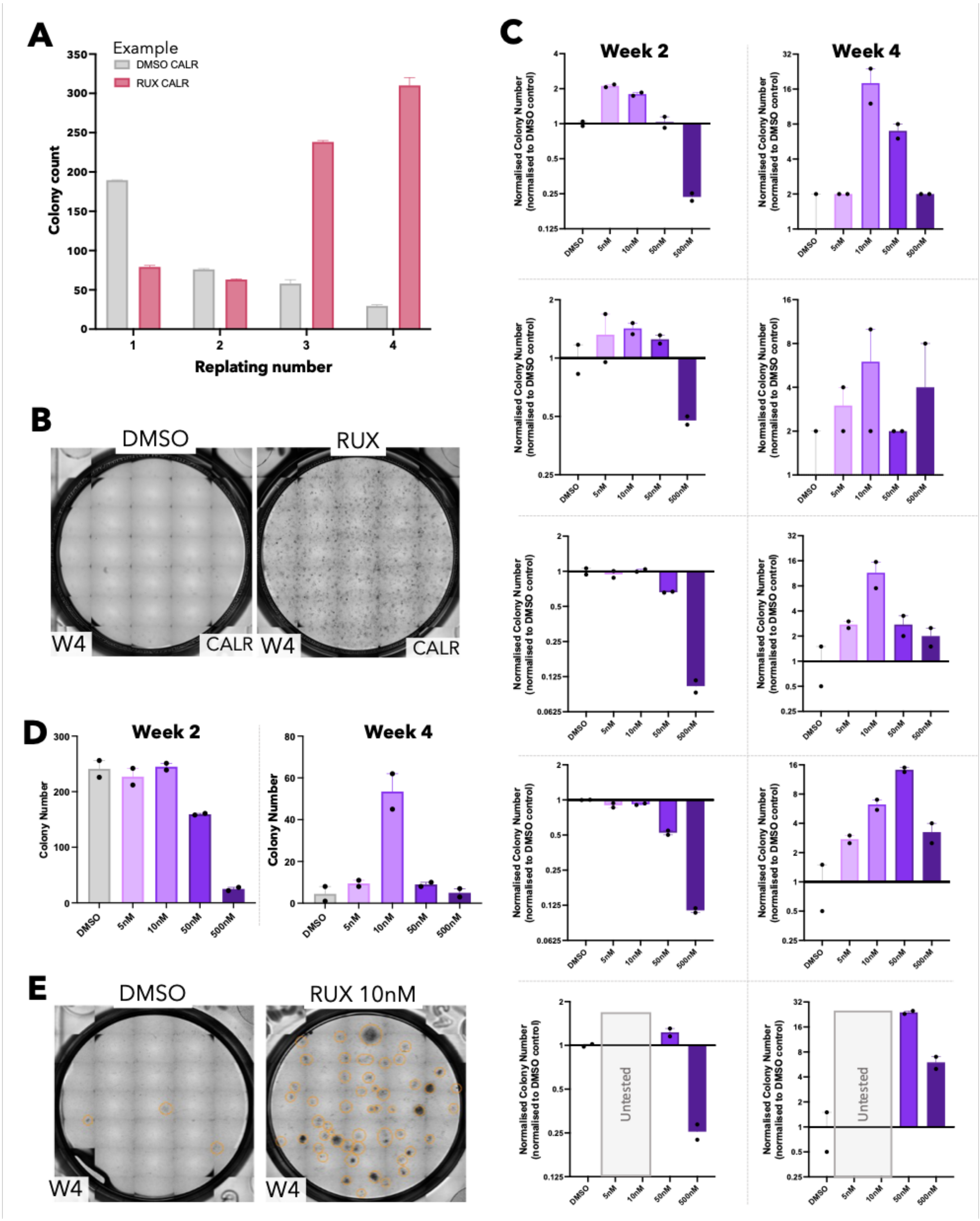

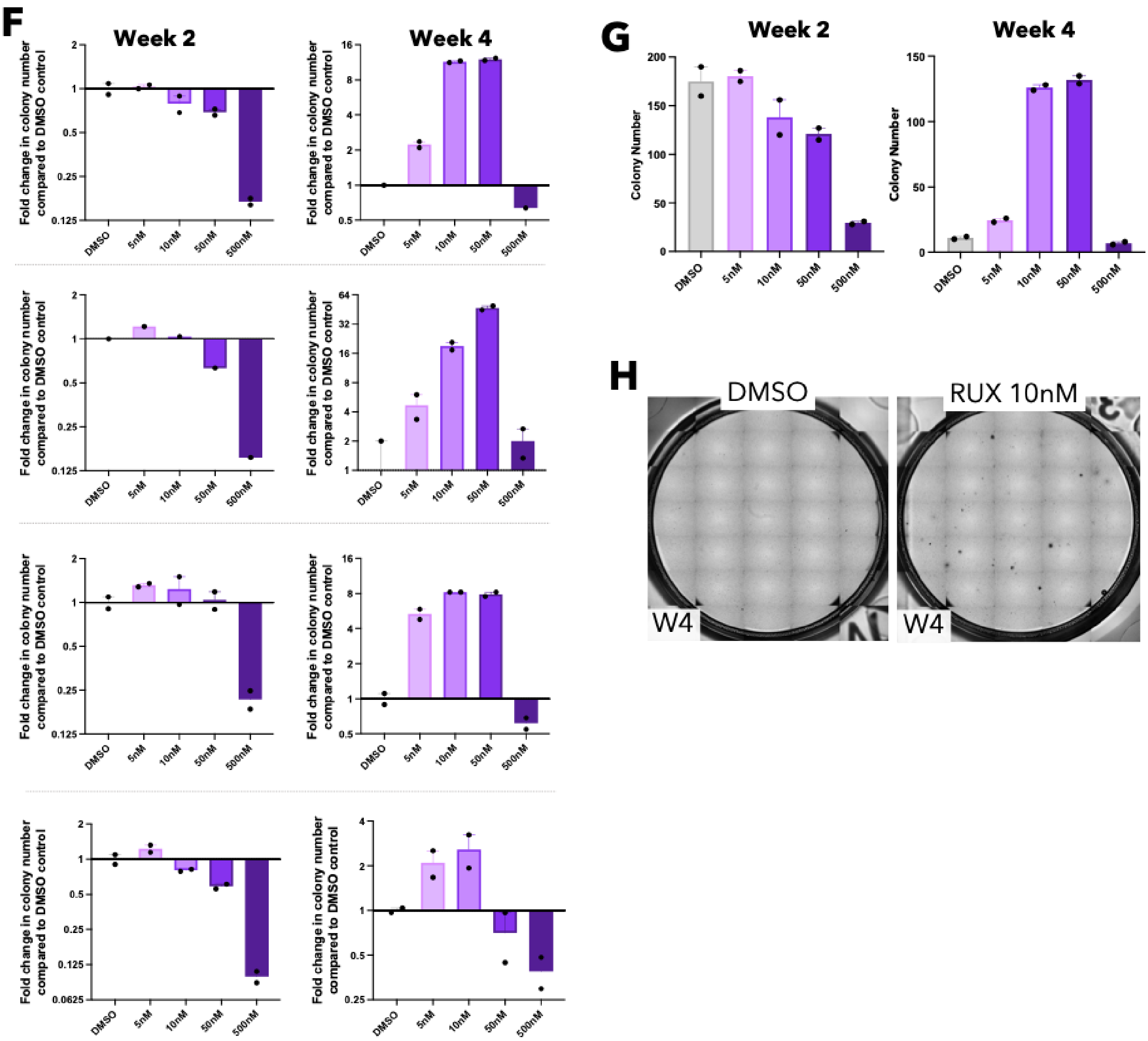

Supplementary Figure 6: Ruxolitinib maintains murine and human myeloproliferative neoplasm HSPCs.

(A) Bar graph showing colony numbers recorded at each replating week for CALR homozygous mutant HSCs from CALRdel/del mice. ESLAM HSCs were treated for 7-days with vehicle or ruxolitinib at 250nM (n=2 technical replicates). (B) Example of colony images showing the number and size difference of the colonies at the final (fourth) week of replating by CALR homozygous mutant HSCs treated

with DMSO or ruxolitinib for 7 days in IL-3/IL-6/SCF cultures. (C) Bar plots showing the number of colonies produced by human HSCs ($CD34^+CD38^-CD45RA^-$) cultured for 7 days in the presence of ruxolitinib, normalised to the number of colonies produced by HSCs cultured for 7 days with DMSO (fold change). Each row of the bar graphs represents data from an individual donor. Left bar graph showing the fold-change in colony numbers in the first round of colony formation (2 weeks in methylcellulose). Right bar graph showing fold-change colony numbers in the second round of colony formation (4 weeks in methylcellulose). HSCs from 4 individual healthy donor were treated with DMSO, 5nM, 10nM, 50nM, or 500nM of ruxolitinib, which correspond to Figure 6G in the main text. A 5th healthy donor was included, which was from a preliminary test where fewer doses of ruxolitinib were tested (50nM and 500nM; bottom row). (D) Bar plots showing the colony numbers (mean ± SEM; n=2 technical replicates shown as dots) at the first plating (left) and the second plating (right), corresponding to normalised data in *Supl. 6C*. (E) Example images showing number and size of colonies produced by healthy human donor HSCs cultured with DMSO or ruxolitinib for 7 days, at the final plating (4-weeks). (F) Bar plots showing the number of colonies produced by HSCs ($CD34^+CD38^-CD45RA^-$) that were sorted from human myelofibrosis patient and cultured for 7 days in the presence of ruxolitinib, data were normalised to the number of colonies produced by HSCs cultured for 7 days with DMSO. Each row represents data from an individual donor. Left graph showing normalised colony numbers (n=2 technical replicates) in the first round of colony formation (2 weeks in methylcellulose). Right graph showing normalised colony numbers in the second round of colony formation (4 weeks in methylcellulose). (G) Bar plots showing the colony numbers (mean ± SEM; n=2 technical replicates) at the first plating (left)

and the second plating (right), corresponding to normalised data in F. (H) Example images showing size and number of colonies produced by myelofibrosis patient HSCs cultured with DMSO or ruxolitinib for 7 days, at the final plating (4-weeks).

Table legends

Table 1: Differentially expressed genes in STAT5-deficient HSCs. ESLAM HSCs from WT and STAT5-deficient ESLAM mice were FACS isolated from hind leg bones before plate based scRNA sequencing by Smartseq2 platforms. The transcriptomes of WT and $STAT5^{-/-}$ ESLAMs were compared by differential gene expression (DEx) analysis. All detected differentially expressed genes are listed.

Table 2: Significantly enriched and depleted pathways in STAT5 deficient HSCs. Significantly enriched or depleted gene sets in STAT5-deficient ESLAM HSCs from Hallmarks and KEGG databases as determined by gene set enrichment analysis (GSEA) from ranked differentially expressed gene lists. Gene sets with an FDR q-value of less than 0.25 were considered significantly enriched in STAT5 deficient HSCs.

Table 3: Differentially expressed genes in uSTAT5B overexpressing transcriptionally defined LT-HSCs. WT ELSAM HSCs were transduced with virus and cultured for 5 days in IL-11/SCF conditions[2] before 10X scRNA sequencing. The transcriptomes of transcriptionally isolated LT-HSCs infected with lentivirus containing STAT5B-YF were compared to the transcriptomes of EV

transduced LT-HSCs. Differential gene expression (DEx) analysis was performed. All detected differentially expressed genes are listed.

Table 4: Differentially expressed genes in ruxolitinib treated transcriptionally defined LT-HSCs. WT ESLAM HSCs were cultured in IL3/IL-6/SCF conditions for 5 days with DMSO or ruxolitinib (250nM) before 10X scRNA sequencing. The transcriptomes of transcriptionally isolated LT-HSCs treated with ruxolitinib were compared to the transcriptomes of LT-HSCs treated with DMSO. Differential gene expression (DEx) analysis was performed. All detected differentially expressed genes are listed.

Table 5: Colony counts from serial replating of human healthy donor HSCs cultured with ruxolitinib or DMSO. Healthy human platelet apheresis donor HSCs were harvest from apheresis filters and placed into high cytokine cultures (EXPER[15]) for 7 days with ruxolitinib (5nM, 10nM, 50nM or 500nM) at 400 cells per well. Cells were harvested and placed into serial colony replating assays. After 2 weeks, colonies were counted and well contents were harvested and diluted for further colony formation; colonies were counted after 2 weeks.

Table 6: Colony counts from serial replating of myelofibrosis donor HSCs cultured with ruxolitinib or DMSO. Circulating HSCs were harvest from the peripheral blood of myelopoiesis patients and placed into high cytokine cultures (EXPER[15]) for 7 days with ruxolitinib (5nM, 10nM, 50nM or 500nM) at 400 cells per well. Cells were harvested and placed into serial colony replating assays. After 2

weeks, colonies were counted and well contents were harvested and diluted for further colony formation; colonies were counted after 2 weeks.

References

1. Balazs AB, Fabian AJ, Esmon CT, Mulligan RC. Endothelial protein C receptor (CD201) explicitly identifies hematopoietic stem cells in murine bone marrow. *Blood*. 2006;107(6):2317–2321.
2. Kent DG, Dykstra BJ, Cheyne J, Ma E, Eaves CJ. Steel factor coordinately regulates the molecular signature and biologic function of hematopoietic stem cells. *Blood*. 2008;112(3):560–567.
3. Daniel P, Jung PH, Sam W, et al. The stem/progenitor landscape is reshaped in a mouse model of essential thrombocythemia and causes excess megakaryocyte production. *Sci Adv*. 2022;6(48):eabd3139.
4. Zerbino DR, Achuthan P, Akanni W, et al. Ensembl 2018. *Nucleic Acids Res*. 2018;46(D1):D754–D761.
5. Wu TD, Nacu S. Fast and SNP-tolerant detection of complex variants and splicing in short reads. *Bioinformatics*. 2010;26(7):873–881.
6. Anders S, Pyl PT, Huber W. HTSeq—a Python framework to work with high-throughput sequencing data. *bioinformatics*. 2015;31(2):166–169.
7. Brennecke P, Anders S, Kim JK, et al. Accounting for technical noise in single-cell RNA-seq experiments. *Nat Methods*. 2013;10(11):1093–1095.
8. Ritchie ME, Phipson B, Wu DI, et al. limma powers differential expression analyses for RNA-sequencing and microarray studies. *Nucleic Acids Res*. 2015;43(7):e47–e47.
9. Wolf FA, Angerer P, Theis FJ. SCANPY: large-scale single-cell gene expression data analysis. *Genome Biol*. 2018;19(1):1–5.
10. Love MI, Huber W, Anders S. Moderated estimation of fold change and dispersion for RNA-seq data with DESeq2. *Genome Biol*. 2014;15(12):1–21.
11. Cumaraswamy AA, Lewis AM, Geletu M, et al. Nanomolar-Potency Small Molecule Inhibitor of STAT5 Protein. *ACS Med Chem Lett*. 2014;5(11):1202–1206.
12. Subramanian A, Tamayo P, Mootha VK, et al. Gene set enrichment analysis: a knowledge-based approach for interpreting genome-wide expression profiles. *Proceedings of the National Academy of Sciences*. 2005;102(43):15545–15550.
13. Dahlin JS, Hamey FK, Pijuan-Sala B, et al. A single cell hematopoietic landscape resolves eight lineage trajectories and defects in Kit mutant mice. *Blood*. 2018;blood-2017-12-821413.
14. Nestorowa S, Hamey FK, Pijuan Sala B, et al. A single-cell resolution map of mouse hematopoietic stem and progenitor cell differentiation. *Blood*. 2016;128(8):e20–e31.
15. Belluschi S, Calderbank EF, Ciaurro V, et al. Myelo-lymphoid lineage restriction occurs in the human haematopoietic stem cell compartment before lymphoid-primed multipotent progenitors. *Nat Commun*. 2018;9(1):4100.
16. Nosaka T, Kawashima T, Misawa K, et al. STAT5 as a molecular regulator of proliferation, differentiation and apoptosis in hematopoietic cells. *EMBO J*. 1999;18(17):4754-4765–4765.
17. Matsumura I, Kitamura T, Wakao H, et al. Transcriptional regulation of the cyclin D1 promoter by STAT5: its involvement in cytokine-dependent growth of hematopoietic cells. *EMBO J*. 1999;18(5):1367-1377–1377.
18. Aichberger KJ, Mayerhofer M, Krauth M-T, et al. Identification of mcl-1 as a BCR/ABL-dependent target in chronic myeloid leukemia (CML): evidence for cooperative antileukemic effects of imatinib and mcl-1 antisense oligonucleotides. *Blood*. 2005;105(8):3303–3311.
19. Schepers H, Wierenga ATJ, Vellenga E, Schuringa JJ. STAT5-mediated self-renewal of normal hematopoietic and leukemic stem cells. *JAKSTAT*. 2012;1(1):13–25.
20. Fatrai S, Wierenga ATJ, Daenen SMGJ, Vellenga E, Schuringa JJ. Identification of HIF2α as an important STAT5 target gene in human hematopoietic stem cells. *Blood*. 2011;117(12):3320–3330.
21. Cabezas-Wallscheid N, Buettner F, Sommerkamp P, et al. Vitamin A-Retinoic Acid Signaling Regulates Hematopoietic Stem Cell Dormancy. *Cell*. 2017;169(5):807-823.e19.

22. Pietras EM, Lakshminarasimhan R, Techner J-M, et al. Re-entry into quiescence protects hematopoietic stem cells from the killing effect of chronic exposure to type I interferons. *Journal of Experimental Medicine*. 2014;211(2):245–262.
23. Park HJ, Li J, Hannah R, et al. Cytokine-induced megakaryocytic differentiation is regulated by genome-wide loss of a uSTAT transcriptional program. *EMBO J*. 2016;
